# A regulatory phosphorylation site on Mec1 controls chromatin occupancy of RNA polymerases during replication stress

**DOI:** 10.15252/embj.2021108439

**Published:** 2021-09-27

**Authors:** Verena Hurst, Kiran Challa, Felix Jonas, Romain Forey, Ragna Sack, Jan Seebacher, Christoph D Schmid, Naama Barkai, Kenji Shimada, Susan M Gasser, Jérôme Poli

**Affiliations:** ^1^ Friedrich Miescher Institute for Biomedical Research Basel Switzerland; ^2^ Faculty of Natural Sciences University of Basel Basel Switzerland; ^3^ Departments of Molecular Genetics and Physics of Complex Systems Weizmann Institute of Science Rehovot Israel; ^4^ Institut de Génétique Humaine CNRS Université de Montpellier Equipe labélisée Ligue contre le Cancer Montpellier France

**Keywords:** Mec1, nuclear pore, replication checkpoint, replication interference, replication stress, transcription, Chromatin, Transcription & Genomics, DNA Replication, Recombination & Repair, Post-translational Modifications & Proteolysis

## Abstract

Upon replication stress, budding yeast checkpoint kinase Mec1^ATR^ triggers the downregulation of transcription, thereby reducing the level of RNA polymerase (RNAP) on chromatin to facilitate replication fork progression. Here, we identify a hydroxyurea‐induced phosphorylation site on Mec1, Mec1‐S1991, that contributes to the eviction of RNAPII and RNAPIII during replication stress. The expression of the non‐phosphorylatable *mec1‐S1991A* mutant reduces replication fork progression genome‐wide and compromises survival on hydroxyurea. This defect can be suppressed by destabilizing chromatin‐bound RNAPII through a TAP fusion to its Rpb3 subunit, suggesting that lethality in *mec1‐S1991A* mutants arises from replication–transcription conflicts. Coincident with a failure to repress gene expression on hydroxyurea in *mec1‐S1991A* cells, highly transcribed genes such as *GAL1* remain bound at nuclear pores. Consistently, we find that nuclear pore proteins and factors controlling RNAPII and RNAPIII are phosphorylated in a Mec1‐dependent manner on hydroxyurea. Moreover, we show that Mec1 kinase also contributes to reduced RNAPII occupancy on chromatin during an unperturbed S phase by promoting degradation of the Rpb1 subunit.

## Graphical abstract

Hydroxyurea directly triggers a checkpoint kinase Mec1/ATR function for limiting replication‐transcription conflicts in budding yeast.

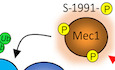

## Introduction

DNA replication puts genome stability at risk, largely because of impediments to replication fork progression. These result from both chemical modifications of the template and the tight binding of enzymatic complexes to DNA (García‐Muse & Aguilera, 2016). Among these, the collision of the replication and transcription machineries is the most pronounced. The major response to replication fork stalling is activation of the ATR‐ATRIP checkpoint kinase (Mec1‐Ddc2 in *Saccharomyces cerevisiae*), which is recruited to single‐stranded DNA (ssDNA) at stalled forks. RPA, together with the 9‐1‐1 ds‐ssDNA junction binding complex (*Sc* Rad17, Mec3, and Ddc1) and TOPBP1 (*Sc* Dpb11) activate the replication checkpoint kinase Mec1^ATR^, initiating a cascade of events controlled by downstream checkpoint kinases (Hustedt *et al*, 2013; Hamperl & Cimprich, 2016).

The Mec1^ATR^ target Rad53 (CHK1 in mammals) is the main effector kinase in the DNA replication checkpoint (DRC) cascade, yet there are many fork‐related events that depend directly on phosphorylation mediated by Mec1, and not on Rad53 (BastosdeOliveira *et al*, 2015; Hustedt *et al*, 2015; Lanz *et al*, 2018). Indeed, the loss of Mec1 leads to much higher levels of spontaneous gross chromosomal rearrangements (GCRs) than the loss of Rad53 (Myung *et al*, 2001). Phosphoproteomic studies have identified Mec1‐specific targets in S phase cells both in the absence and in the presence of hydroxyurea (HU) (BastosdeOliveira *et al*, 2015; Hustedt *et al*, 2015). Accordingly, a combination of genetics and phosphoproteomics showed that many of Mec1's pro‐replicative and anti‐GCR functions are independent of downstream checkpoint kinases (Hustedt *et al*, 2015; Lanz *et al*, 2018). Whereas Rad53 induces cell cycle arrest, increases dNTP biosynthesis, downregulates late origins, and promotes stalled fork recovery, Mec1 ensures replisome stability (Cobb *et al*, 2003, 2005) and controls RNA polymerase occupancy in the presence of HU (Poli *et al*, 2016).

Transcription is a widespread source of obstacles encountered by the moving replisome, including RNA:DNA hybrids, positive DNA supercoiling, and RNA polymerases themselves (Gómez‐González & Aguilera, 2019). Genome organization minimizes the negative impact of transcription on DNA replication by separating the two processes in space and time (Meryet‐Figuiere *et al*, 2014), yet transcription–replication conflicts inevitably occur. These are strongly enhanced by oncogenic transformation, which leads to promiscuous and untimely origin firing (Kotsantis *et al*, 2016; Macheret & Halazonetis, 2018). The pausing of the replication fork due to transcription can occur both when the machineries move in the same direction (codirectional conflicts) and when they move toward each other (head‐on collision) (García‐Muse & Aguilera, 2016; Hamperl *et al*, 2017). In both bacteria and eukaryotes, head‐on conflicts cause a greater proportion of replication fork damage and induce higher rates of genome instability (Prado & Aguilera, 2005; Boubakri *et al*, 2010; Hamperl *et al*, 2017; Lang & Merrikh, 2021).

Multiple mechanisms help cells avoid or resolve transcription–replication conflicts. Both prokaryotic and eukaryotic cells express specific DNA helicases that remove proteins and/or RNA:DNA hybrids that hinder replisome progression (Boubakri *et al*, 2010; Andrs *et al*, 2020). Among these are the yeast Pif1 and Rrm3 helicases, which facilitate replication through tRNA genes and other stable DNA–protein complexes (Ivessa *et al*, 2003; Osmundson *et al*, 2017; Tran *et al*, 2017), and Sen1/Senataxin, which dissolves the RNA:DNA hybrids found at highly transcribed genes (Alzu *et al*, 2012; Brambati *et al*, 2018). Interestingly, mutations in RNA polymerase II (RNAPII) itself compromise the resolution of replication–transcription conflicts (Felipe‐Abrio *et al*, 2015), and at tRNA genes, a transient repression of RNAPIII helps ensure replication fork passage during replication stress (Nguyen *et al*, 2010; Bhalla *et al*, 2019). Other stress‐induced mechanisms also reduce or resolve transcription–replication conflicts. For example, upon a sudden increase in transcription, multiple kinases act on the replication fork factor Mrc1 to slow fork progression and limit transcription‐associated recombination events (Duch *et al*, 2013, 2018). On the other hand, yeast Mec1 works together with the chromatin remodeler INO80 and PAF1, a transcription elongation complex, to reduce RNAPII occupancy in the presence of HU (Lafon *et al*, 2015; Poli *et al*, 2016). Similarly, human ATR triggered the degradation of the histone chaperone ASF1a, to reduce transcription in the vicinity of stalled replication forks (Im *et al*, 2014).

In budding yeast, a double point mutant that alters two residues in the spacer region between kinase and FAT (TPR) repeat domains of Mec1 (called *mec1‐100*) renders cells sensitive to HU, but not to ultraviolet light (UV) nor methyl methanesulfonate (MMS) (Paciotti *et al*, 2001; Cobb *et al*, 2005). The *mec1‐100* mutant accentuates replication fork collapse even though the downstream Rad53 kinase can be activated to trigger a G_2_/M arrest in response to DNA damage (Hustedt *et al*, 2015). Replication fork rates are reduced in *mec1‐100* cells, coincident with a less open chromatin structure (Rodriguez & Tsukiyama, 2013), and the ablation of multiple regulators of RNAPII transcription, including the PAF1 complex and seven nucleosome remodelers, shows conditional synthetic lethality with *mec1‐100* on HU (Poli *et al*, 2016). Intriguingly, Mec1 itself is phosphorylated on S1991 in a *mec1‐100* sensitive manner upon exposure to HU (Hustedt *et al*, 2015). We note that the mammalian ATR kinase harbors an autophosphorylation site at a nearby residue, T1989, that activates this conserved kinase (Liu *et al*, 2011; Nam *et al*, 2011).

Here, we studied the role of the yeast Mec1 kinase in the dynamics of RNA polymerases II and III during S phase, in particular under conditions of HU‐induced replication stress. In the Mec1 protein, we replaced the S phase‐specific S1991 phosphoacceptor site by alanine, which cannot be phosphorylated, or by aspartic acid, which in some cases mimics phosphorylation. Importantly, the *mec1‐S1991A* mutant is sensitive to HU and shows strong negative genetic interactions with Rrm3 and Sgs1, two DNA helicases assisting replisome progression through obstacles, as well as with the INO80 and PAF1 complexes, which help evict RNAPII from chromatin during replication stress. The *mec1‐S1991A* mutant shows replication fork progression defects genome‐wide on HU and selectively alters phosphorylation of targets involved in transcription control. Importantly, *mec1‐S1991A* sensitivity to HU can be suppressed by reducing RNAPII occupancy on chromatin. We propose that phosphorylation of Mec1‐S1991 promotes DNA replication under stress conditions by limiting the conflicts between either RNAPII or RNAPIII and the replication fork. Interestingly, we also find that the catalytic RNAPII subunit Rpb1 is partially degraded in S phase and is restored in G_2_, in the absence of exogenous inducers of replication stress.

## Results

### Hydroxyurea‐induced replication stress reduces chromatin‐associated transcription complexes

**Figure 1:**
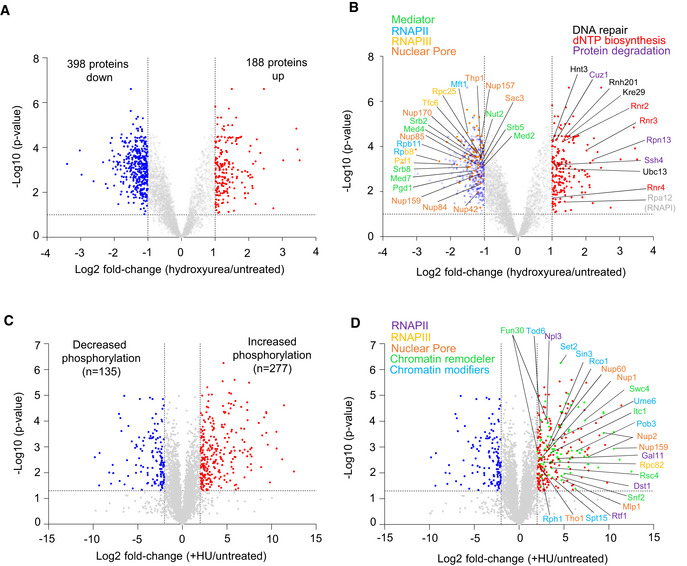
Genome‐wide proteomic response to HU‐induced replication stress A, BVolcano plots showing hydroxyurea‐induced changes in chromatin bound proteins in *sml1Δ* cells (log_2_ ratio [HU‐treated/untreated]). (A) Colored dots are factors with significantly different chromatin binding scores HU‐treated/untreated (adj. *P* < 0.1 and fold change < 0.5 [blue] or > 0.5 [red]). (B) Factors involved in transcription with reduced occupancy upon HU treatment are named (left), as well as factors involved in replication stress response with increased occupancy (right).C, DPhosphopeptide abundances (log_2_ ratio WT [HU/untreated]). Colored dots are factors with significantly different phosphopeptide scores in WT (HU/untreated) < 4 (blue) or > 4 (red) and *P*‐value < 0.05 (Student's paired *t*‐test, biological replicates *n* = 3; significant phosphopeptides *n* = 135 and *n* = 277, respectively). (D) Among the phosphopeptides induced on HU, factors involved in transcription are highlighted (green dots, *n* = 87). Full list in Dataset EV4.

We have shown that RNAPII abundance on chromatin is reduced at sites of DNA replication during HU‐induced replication stress (Poli *et al*, 2016). A study of changes in locus‐specific chromatin‐bound factors in the presence and absence of HU has confirmed these results, and identified other RNAPII transcriptional cofactors, for which the abundance decreased upon HU stress, at a single locus (Korthout *et al*, 2018). To understand how the entire chromatin proteome (chromatome) responds to replication stress, we examined the global complement of chromatin‐bound proteins in S‐phase yeast cells in the presence and absence of HU. The chromatome identified 586 proteins that fulfilled a combined criterion for enrichment or depletion (Log_2_ FC > |1|, FDR < 0.1) when comparing HU vs. untreated conditions. Among these, 398 showed reduced occupancy, whereas 188 showed increased binding to chromatin (Fig 1A). Among proteins with increased chromatin occupancy on HU, we detected one subunit of RNAPI (Rpa12), which regulates the termination of rRNA synthesis (Fig 1B), and several subunits of the dNTP biosynthesis enzyme ribonucleotide reductase (Rnr2, Rnr3, and Rnr4), along with factors involved in proteasome‐mediated protein degradation and DNA repair (Fig 1B and Appendix Fig S1A, Dataset EV1). The increase in repair and proteasome components parallels the recently reported yeast chromatome changes observed after exposure to oxidative damage (Challa *et al*, 2021). An analysis of the proteins showing reduced occupancy on HU revealed a strong drop in abundance of factors involved in the transcription process, including RNAPII, RNAPIII, mediator, and the nuclear pore complex (Fig 1B, Datasets EV2 and EV3). This was confirmed by a survey of Gene Ontology (GO) terms, which ranked the regulators and machinery of RNA synthesis as mostly significantly depleted (Appendix Fig S1B).

In parallel, we performed a quantitative phosphoproteomic study that monitored differential phosphorylation between wild‐type cells growing exponentially and those undergoing HU‐induced stress (Fig 1C and D). In this analysis, we identified 412 peptides that were differentially phosphorylated on HU, including 277 that show increased phosphorylation (Fig 1C, Dataset EV4). Importantly, enriched among HU‐induced phosphotargets were components of the same transcription‐regulatory complexes showing depletion in the HU‐treated chromatome, including subunits of RNAPII, RNAPIII, the nuclear pore complex, and several chromatin remodelers/modifiers (Fig 1D, Dataset EV5). Thus, HU‐induced replication stress provoked a general drop in mediator and other RNA polymerase cofactors on the level of the chromatome (Fig 1A and B) and led to the phosphorylation of a subset of transcription regulators (Fig 1C and D).

### Mec1 phosphorylation on Ser 1991 promotes survival during HU‐induced replicative stress

**Figure 2:**
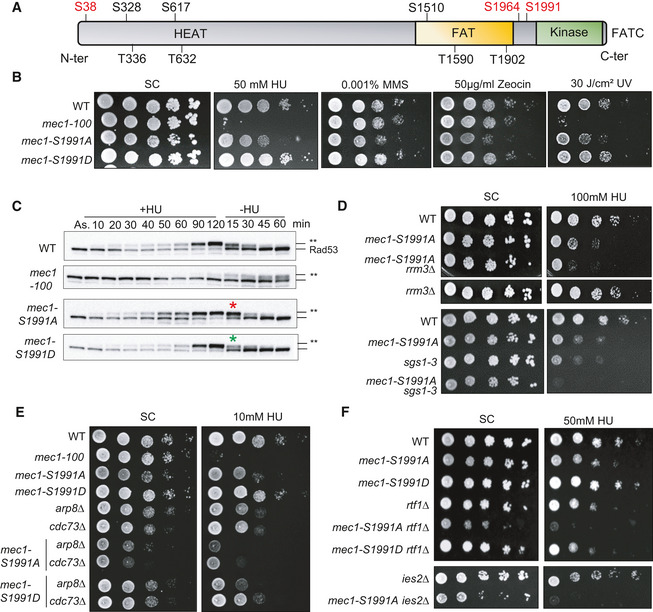
Mec1 phosphorylation on Serine 1991 helps cells cope with replication stress AThe domain structure of Mec1, which is typical for PI3K‐related protein kinases, including the N‐terminal HEAT repeat domain, FAT domain, kinase domain, and the C‐terminal FAT domain. The Mec1 consensus SQ/TQ sites identified *in silico* are marked in black, while *in vivo* validated phosphorylation sites induced by genotoxic agents are in red.BA 10‐fold dilution series of cells from exponential SC cultures of the indicated strains were spotted on SC +/− the indicated dose of genotoxic agents. Imaged after 3 days growth.CAsynchronous exponentially growing cells were treated with 0.2 M HU (+HU); then, HU was removed from the medium (−HU). SDS–PAGE of total protein extracts taken at the indicated times were used to detect the kinetic of Rad53 phosphorylation upshift (Rad53‐P = **). Red and green asterisks indicate the time where *mec1‐S1991* phosphomutants show altered Rad53 recovery.D–FDilution series of cells from exponential SC cultures of the indicated strains were spotted on SC +/− the indicated dose of HU. Panels D and F are 5‐fold dilution series, while panel F is 10‐fold. The SC control for the top four strains is the same as Panel B control. Panel D is imaged at day 2. Source data are available online for this figure.

The *mec1‐100* mutant (F1179S, N1700S) shows selective hypersensitivity to HU (Paciotti *et al*, 2001; Cobb *et al*, 2005). Among the many potential phosphoacceptor sites on Mec1 itself (Fig 2A (Memisoglu *et al*, 2019)), only two stress‐induced phosphorylation sites on the Mec1 kinase itself (S38 and S1991) fail to be phosphorylated in the *mec1‐100* mutant (Hustedt *et al*, 2015), and of these, only phospho‐S1991 contributes to the survival of cells facing replication stress (Fig 2A and B). This is consistent with the observation that the S1991 phosphorylation is S phase specific and is upregulated on HU (Hustedt *et al*, 2015). We therefore examined the role of Mec1‐S1991 modification in control of transcription‐replication conflicts (TRC).

We introduced phosphomimetic and non‐phosphorylable mutants of Mec1‐S1991 (hereafter *mec1‐S1991D* and *mec1‐S1991A,* respectively) at the endogenous *MEC1* locus (Fig 2A). Although the non‐phosphorylatable mutant *mec1‐S1991A* was hypersensitive only to Zeocin on rich YPAD media (Hustedt *et al*, 2015), we observed partially impaired growth in the presence of HU or MMS, and after UV irradiation, on synthetic medium containing glucose (Fig 2B). Growth impairment was not as pronounced as for *mec1‐100*, but given that the *S1991A* mutant, but not *S1991D*, was conditionally impaired, we pursued the analysis of its phenotypes.

We first checked whether *mec1‐S1991A* compromises Rad53 checkpoint activation during HU‐induced stress or its deactivation after removal of HU, by monitoring HU‐induced changes in Rad53 mobility by Western blot. Contrary to *mec1‐100*, which impairs Rad53 activation in S phase but allows its activation as cells with damage accumulate at G_2_/M (Fig 2C), we see that both the *mec1‐S1991A* and *mec1*‐*S1991D* alleles are fully proficient for activating Rad53 on HU (Fig 2C, +HU). We note that in *mec1‐S1991A* cells Rad53 dephosphorylation kinetics may be slightly slower, and in *mec1‐S1991D* slightly more rapid, than in the isogenic wild‐type strain (Fig 2C, −HU at 15 min, red/green asterisks). This may reflect the kinetics of Pph3‐Psy2 phosphatase recruitment (O'Neill *et al*, 2007). Nonetheless, we conclude that the phospho‐resistant *mec1‐S1991A* allele compromises survival of chronic replication stress, despite being fully competent for Rad53 activation.

If the *mec1‐S1991A* allele affected downstream effector kinases of the DRC, one might expect it to be epistatic in combination with loss of Mrc1, a key regulator of Rad53 activation, or Dun1, a downstream kinase of the Mec1‐Rad53 checkpoint cascade (Pardo *et al*, 2017). However, double mutants of *mec1‐S1991A* with either *mrc1Δ* or *dun1Δ* led to enhanced lethality on HU, consistent with Mec1‐S1991p acting in parallel to Rad53 (Appendix Fig S2A). Similarly, the combination of *mec1‐S1991A* with mutants that block recombination‐mediated fork restart (*rad52Δ* and *mre11Δ;* (Yeeles *et al*, 2013)) showed additive sensitivity to HU (Appendix Fig S2B), making it unlikely that Mec1‐S1991p acts exclusively on these factors.

Given the striking change in the transcriptional machinery on HU (Fig 1), we next asked whether the loss of Mec1‐S1991 phosphorylation aggravates phenotypes arising from conflicts between the transcription and replication machineries. Two helicases, the RecQ helicase Sgs1 and the Pif1 family helicase Rrm3 (Muellner & Schmidt, 2020), contribute to replication fork maintenance on HU (Cobb *et al*, 2003; Ivessa *et al*, 2003). Rrm3 displaces nonhistone protein–DNA complexes ahead of the fork to enable replisome progression (Muellner & Schmidt, 2020), whereas Sgs1 contributes to the removal of RNA:DNA hybrids (Chang *et al*, 2017). Sgs1 is also implicated in the reversal of DNA fold‐back structures that lead to stalled replication fork collapse (Cobb *et al*, 2003, 2005) and in the end processing necessary for fork restart (Sanford *et al*, 2021). Consistent with earlier results showing that both helicase mutants are synthetic lethality with *mec1‐100* on HU (Cobb *et al*, 2005; Hustedt *et al*, 2015), the absence of Sgs1 or Rrm3 aggravated the already impaired growth of *mec1‐S1991A* on HU (Fig 2D, Appendix Fig S2C).

These synthetic effects suggested that Mec1‐S1991 phosphorylation might affect pathways dealing directly with the transcriptional machinery. Previous studies have implicated the PAF1 elongation complex and the INO80 chromatin remodeler (INO80C) in resolving TRC by removing RNAPII in front of the replication fork (Lafon *et al*, 2015; Poli *et al*, 2016). We examined the double mutants of *mec1‐S1991A* with PAF1 components *cdc73Δ* or *rtf1Δ,* as well as *arp8Δ* or *ies2Δ,* which compromise INO80C. Interestingly, compromising either complex was strongly synergistic with *mec1‐S1991A* both on HU and in its absence (Fig 2E and F, Appendix Fig S2D), while *mec1‐S1991D* had no combinatorial effects either with or without HU (Fig 2E and F). Thus, the *mec1‐S1991A* mutation recapitulates the synthetic lethality observed previously between *mec1‐100* and loss of PAF1 or INO80C subunits, which control RNAPII removal on HU (Poli *et al*, 2016).

### Mec1‐S1991 phosphorylation promotes replication under HU‐induced stress

**Figure 3:**
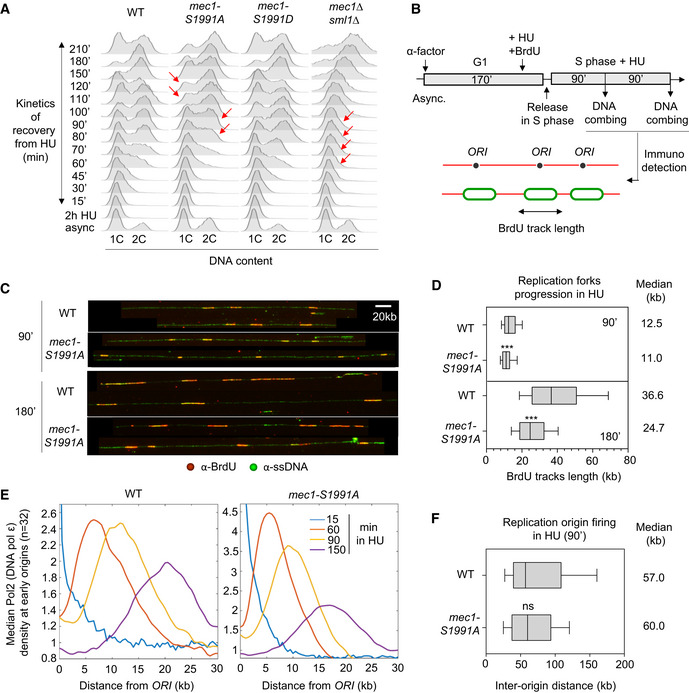
Mec1‐S1991 phosphorylation promotes replication on HU AFlow‐cytometry analysis of DNA content. Asynchronous cells from the indicated strains were treated for 2 h with 0.2 M HU. After HU removal, recovery from replication stress was monitored by FACS (time in min). Red arrows highlight detected delays.B–DAnalysis of replication fork progression at the single‐molecule level by DNA combing. (B) Scheme for the experimental procedure: exponentially growing cells were synchronized in G_1_ with α‐factor and released in S phase in the presence of 0.2 M HU. Newly replicated DNA was labeled with BrdU for 90 and 180 min. (C) Representative images of DNA fibers. Green: ssDNA, red: BrdU. Scale bar corresponds to 20 kb. (D) Graph depicts the distribution of BrdU track length. Box, 25–75 percentile range. Whiskers, 10–90 percentiles range. Median is indicated in kb. ***P‐value < 10^−3^, by Mann–Whitney rank‐sum test. WT 90 min (*n* = 427) and 180 min (*n* = 483), *mec1‐S1991A* 90 min (*n* = 309) and 180 min (*n* = 477).EAnalysis of DNA polymerase ϵ progression by ChIP‐seq in S phase + HU 0.2 M. Median Pol2 (DNA polymerase ϵ) signal at early origins (*n* = 32) is plotted over a 30kb distance. The rate of progression is inferred from the peak at given times.FGraph depicting the distribution of inter‐origin distances (IOD) determined by DNA combing after 90 min in S phase + 0.2 M HU. Box, whiskers, and median as in D. ns (*P*‐value > 0.01), by Mann–Whitney rank‐sum test. WT (*n* = 173) and *mec1‐S1991A* (*n* = 174). The average IOD values were 57.3 kb and 59.8 kb, which were rounded down and up respectively. Source data are available online for this figure.

The synthetic sickness of *mec1‐S1991A* with impaired function of INO80C and PAF1 complexes suggested that the mutant may be unable to deal with impediments to replication fork progression. To see whether the *mec1‐S1991A* mutant itself shows reduced fork progression on HU, we first monitored the resumption of replication by FACS, after an acute 2 h exposure to 0.2 M HU. Although wild‐type and *mec1‐S1991D* cells resumed replication and fully duplicated their genomes by 150 min, *mec1‐S1991A* cells showed a slight delay in recovery after HU removal (red arrows, Fig 3A). This phenotype, however, was mild when compared to the full *mec1* deletion (*mec1Δ sml1Δ;* Fig 3A). Neither *mec1‐S1991A* nor *mec1*‐*S1991D* alleles showed alterations in the G_1_/S phase transition or in progression through an unperturbed S phase as monitored by FACS (Appendix Fig S3A).

To quantify fork progression defects, we monitored DNA synthesis at the level of individual replication forks using DNA combing (Fig 3B and C). Consistent with the FACS analysis, we found that replication forks synthesize shorter stretches in *mec1‐S1991A* as compared with wild‐type cells, in both early (90 min in HU; 11 vs 12.5 kb; Fig 3C and D) and mid‐S phase (180 min in HU; 24.7 vs 36.6 kb; Fig 3C and D). We then compared DNA polymerase ϵ (Polϵ) progression from early firing origins on HU, measuring DNA polymerase position precisely by Pol2‐6HA chromatin immunoprecipitation and deep sequencing. This analysis revealed a 25% decrease in the rate of replisome progression, which is readily seen at 90‐ and 150‐min timepoints on HU (Fig 3E), consistent with the DNA combing results. This is not due to an altered use or timing of origin firing on HU (Poli *et al*, 2012), as inter‐origin distances scored on individual DNA fibers in early S phase, showed no significant difference between average inter‐origin distances in *mec1‐S1991A* and wild‐type cells (Fig 3F).

To monitor the activity of individual origins, we determined the relative degree of genome duplication in early S phase after 60 min in HU, by monitoring copy number. Wild‐type, *mec1‐S1991A,* and *mec1‐S1991D* alleles showed nearly identical patterns of replication (Appendix Fig S3B), suggesting that replication origins were activated at their dedicated wild‐type time of replication (*T*_rep_) (Yabuki *et al*, 2002) in the mutants (Appendix Fig S3C). Quantifying the number of activated origins in *mec1‐S1991* mutants showed that late replication origins are repressed as in wild‐type cells (Appendix Fig S3D), consistent with the fact that the Rad53 checkpoint is functional (Fig 2C) (Crabbé *et al*, 2010). Taken together, these results argue that Mec1‐S1991 phosphorylation has an important role in promoting replication fork progression under conditions of replication stress, but does not affect origin firing nor the downstream checkpoint response.

### Mec1‐S1991 phosphorylation downregulates RNAPIII transcription during HU‐induced replicative stress

**Figure 4:**
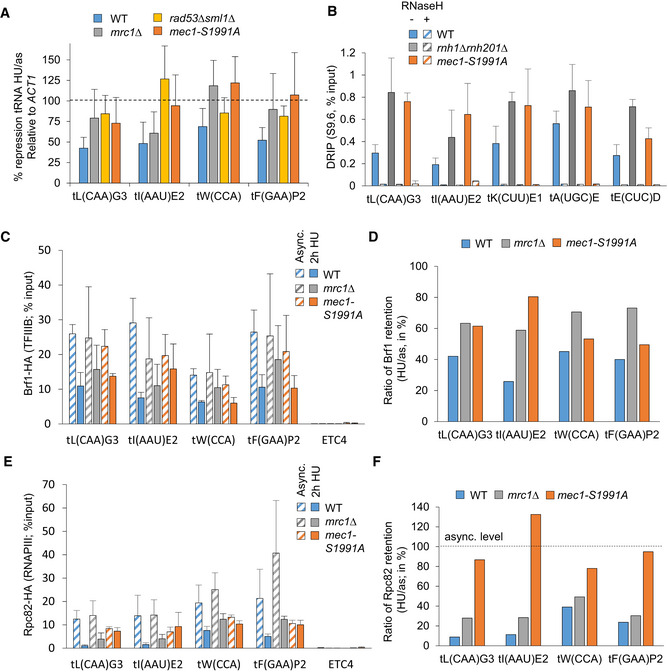
Mec1‐S1991 phosphorylation limits RNAPIII transcription on HU AtRNA level measured by RT–qPCR in asynchronous (async.) culture and after 90 min on HU in the indicated strains. Expression is normalized to *ACT1*. The repression is expressed as a ratio of HU‐treated/asynchronous cells in percent. SEM (*n* = 3 biological replicates) is indicated.BDNA–RNA hybrid level measured by DRIP‐qPCR at several tRNA loci in asynchronous cells of the indicated genotype. As a technical control, samples were treated with RNaseH (+RNaseH, striped columns). SEM (*n* = 3 biological replicates) is indicated.C–FTFIIIB (Brf1‐3HA) and RNAPIII (Rpc82‐3HA) ChIP‐qPCR was performed in asynchronous (async.) and after 2 h in S phase + 0.2 M HU. Enrichment was quantified at several tRNA loci. Data are expressed as percentage of input. ETC4 serves as a control locus which does not recruit RNAPIII. SEM (*n* = 3 biological replicates) is indicated. (D, F) Graph depicts the mean percentage of Brf1 or Rpc82 kept on chromatin after HU treatment, calculated as a ratio HU/async in the indicated strain. Occupancy levels are derived from ChIP‐qPCR values.

**Figure EV1:**
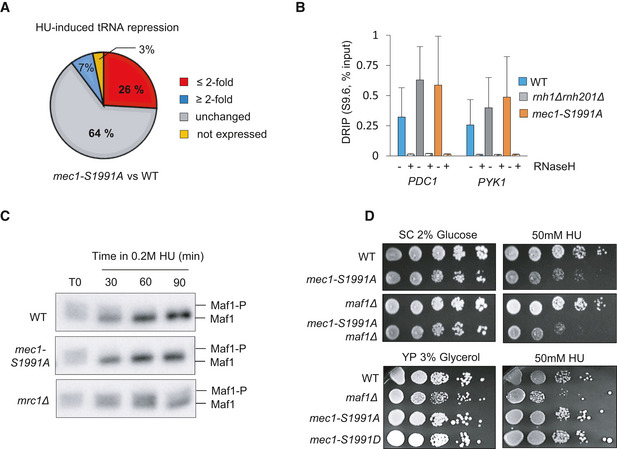
Mec1‐S1991 phosphorylation limits RNAPIII transcription during HU‐induced replication stress ARNA‐seq signals obtained from asynchronous culture and after 90 min of HU treatment allowed to determine the fold change of HU‐induced tRNA repression (HU/as) in wild‐type and *mec1‐S1991A* cells. Pie charts illustrate the proportion of tRNA genes that are either not expressed (yellow), unchanged in steady‐state level +/− HU treatment (unchanged, gray) and differentially repressed upon HU treatment in the *mec1‐S1991A* mutant compared with wild‐type cells (decreased repression in the mutant over wild‐type ≤ 2‐fold in red, increased ≥ 2‐fold in blue).BDNA–RNA hybrid level measured by DRIP‐qPCR at *PDC1* and *PYK1* in asynchronous cells of the indicated genotype. Where indicated, samples were treated with RNase H as a technical control. SEM (*n* = 3 biological replicates) is indicated.CAsynchronous cells of the indicated genotype (+Maf1‐3PK) were treated with 0.2 M HU for 30, 60, and 90 min. Total protein extracts were subjected to SDS–PAGE with a low cross‐linked gel for sufficient separation. Differentially phosphorylated forms of Maf1 were detected with a PK antibody.DDrop assay showing a 10‐fold dilution series of cells from exponential SC cultures of the indicated strains that were spotted on SC supplemented either with 2% glucose or 3% glycerol +/− 50 mM HU. Source data are available online for this figure.

The major impediment to replication fork movement is interference created by the transcription machinery, or by RNA:DNA hybrids (Gómez‐González & Aguilera, 2019). The fact that RNAPIII initiation factors were displaced on HU (Fig 1B), and at least one RNAPIII subunit was phosphorylated (Rpc82), suggested that RNAPIII machinery might be targeted by Mec1. Consistent with this possibility, RT–qPCR performed after 90 min on HU showed that in the *mec1‐S1991A* mutant tRNA levels failed to drop as they did in wild‐type strains (Fig 4A, blue vs orange bars). As previously described (Nguyen *et al*, 2010), we also scored this for *mrc1* and *rad53* deletion strains. To determine the proportion of tRNA transcripts controlled by Mec‐S1991 phosphorylation, we performed strand‐specific RNA sequencing in cells with or without 0.2 M HU treatment. We found that although 64% of tRNA genes are properly repressed on HU in *mec1‐S1991A,* roughly a third relied on Mec1‐S1991 phosphorylation (Fig EV1A), and for the majority of these (26% of tRNA genes) repression in the *mec1‐S1991A* mutant was less than 2‐fold that measured in wild‐type cells on HU. This confirms that Mec1‐S1991 phosphorylation contributes to the downregulation of at least a fraction of tRNA genes on HU.

tRNA transcription is known to generate RNA:DNA hybrids (R‐loops) which impede replication fork progression (El Hage *et al*, 2014; Wahba *et al*, 2016). To see whether R‐loops might be contributing to the *mec1‐S1991A*‐dependent replication defects, we immunoprecipitated DNA–RNA hybrids (DRIP) using the S9.6 antibody and monitored specific tRNA‐encoding loci by qPCR. R‐loops were 2‐ to 3‐fold more abundant in *mec1‐S1991A* vs wild‐type cells at five loci (Fig 4B). All signals were sensitive to degradation by RNaseH, confirming that the DRIP assay was specific (Fig 4B). Not surprisingly, we also found RNA:DNA hybrids accumulating as well at two highly transcribed RNAPII loci in *mec1‐S1991A* cells (Fig EV1B). Taken together, our data suggest that Mec1‐S1991 phosphorylation contributes the transcriptional repression of both tRNA loci and highly transcribed RNAPII genes during replication stress, limiting RNA:DNA hybrid accumulation.

Mec1 has been implicated in the removal of the RNAPIII transcription machinery from tRNA genes on HU (Nguyen *et al*, 2010). To test whether this is the pathway is sensitive to Mec1‐S1991 phosphorylation, we performed ChIP‐qPCR of the TFIIIB subunit Brf1 and the RNAPIII subunit Rpc82. The *mec1‐S1991A* mutant was partially compromised for removal of RNAPIII from chromatin upon exposure to HU, and both Brf1 and Rpc82 levels remained higher in the mutant than in wild‐type cells (Fig 4C–F). For Brf1, the effect was equally dependent on *mrc1Δ*, although not for Rpc82. Because the Mec1‐Rad53 checkpoint pathway activates Maf1, a repressor of RNAPIII loading at tRNA genes, it was proposed that Mec1 controls RNAPIII levels on HU through Maf1 (Nguyen *et al*, 2010). However, the *mec1‐S1991A* allele did not abrogate HU‐induced Maf1 activation, unlike *mrc1Δ* (Fig EV1C). Moreover, whereas *maf1*Δ only shows HU sensitivity on glycerol media, *mec1‐S1991A* is sensitive to HU on glucose, and not glycerol (Fig EV1D). Thus, Mec1‐S1991 phosphorylation appears to control RNAPIII eviction on HU through a pathway distinct from that of Mrc1‐Rad53 and Maf1.

### Mec1‐S1991 phosphorylation controls RNAPII transcription on HU

**Figure 5:**
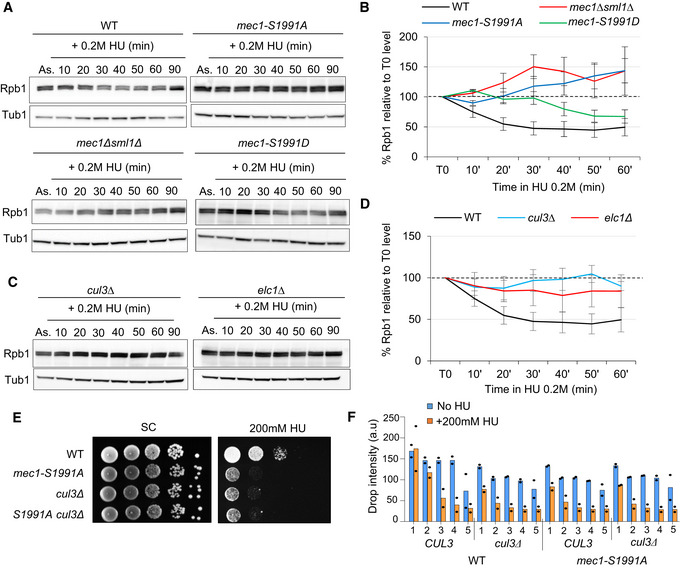
Mec1‐S1991 phosphorylation participates to RNAPII degradation on HU A–DExponentially growing cells were treated with 0.2 M HU, and total protein extracts were collected at the indicated time points (in min), samples were subjected to SDS–PAGE followed by immunoblotting with Rpb1 and Tubulin antibodies. (B, D) Quantitation of total Rpb1 over time was done by normalizing Rpb1 levels to tubulin. The value at the starting point was set to 100% (black dashed line). SEM (at least *n* = 2 biological replicates) is indicated.E, FDrop assay on HU showing a 10‐fold dilution series of cells from exponential SC cultures of the indicated strains that were spotted on SC +/− 200 mM HU. (F) Histogram presents quantification of two independent HU sensitivity assays with mean and individual data point values indicated for each yeast dilution. Source data are available online for this figure.

**Figure EV2:**
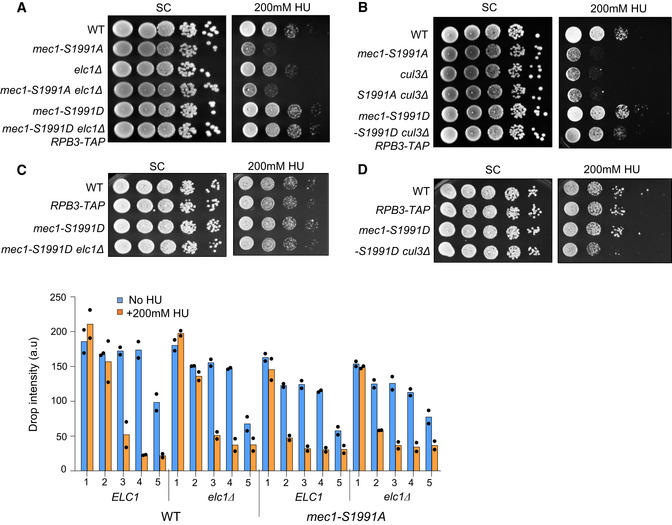
Comparison of *mec1‐S1991A* and *elc1Δ‐cul3Δ* growth defects on HU A–DDrop assays showing a 10‐fold dilution series of cells from exponential SC cultures of the indicated strains that were spotted on SC +/− 200 mM HU. Histogram presents quantification of two independent HU sensitivity assays with mean and individual data point values indicated for each yeast dilution.

We next checked whether Mec1‐S1991 phosphorylation contributes to RNAPII degradation on HU. To do so, we monitored the total level of the RNAPII catalytic subunit Rpb1 during HU‐induced replication stress by Western blot with an Rpb1‐CTD antibody. We detected a rapid decrease in Rpb1 levels to ~50% after the addition of HU in wild‐type cells (Fig 5A and B) while *mec1‐S1991A* cells exhibited impaired Rpb1 degradation in HU, reminiscent of the *mec1Δ* strain (Fig 5A and B). There was a striking 20‐min delay for Rpb1 degradation in the *mec1‐S1991D* mutant, suggesting that the phospho‐mimic does not fully compensate for phospho‐S1991 (Fig 5A and B). Rpb1 degradation during HU‐induced replication stress is proteasome‐dependent (Lafon *et al*, 2015; Poli *et al*, 2016), and it was shown that UV‐induced Rpb1 degradation is mediated by the Cul3‐Elc1 E3 ubiquitin ligase complex (Ribar *et al*, 2007). To test whether Rpb1 degradation on HU also requires Cul3‐Elc1, we monitored Rpb1 levels by Western blot following HU treatment in cells lacking Cul3 or Elc1. As for *mec1‐S1991A*, we observed a failure to degrade Rpb1 in cells lacking either Cul3 or Elc1 (Fig 5C and D). Moreover, the sensitivity of the *mec1‐S1991A* allele for growth on HU was similar to that of *cul3Δ*, and combining the *mec1‐S1991A* allele with either *cul3Δ* or *elc1Δ* mutation showed no additive effects, arguing that the *mec1‐S1991A* defect is likely epistatic to the ubiquitin ligase (Figs 5E and F, and EV2A and B). We note that the *mec1‐S1991D* allele slightly enhanced *cul3Δ* growth on HU, although not that of *elc1Δ* (Fig EV2C and D). Taken together, these results suggest that Mec1‐S1991 phosphorylation acts through the Elc1‐Cul3 ubiquitin ligase complex to promote Rpb1 degradation in the presence of HU.

### Reducing the level of chromatin‐bound RNAPII rescues *mec1‐S1991A* replication defects

**Figure 6:**
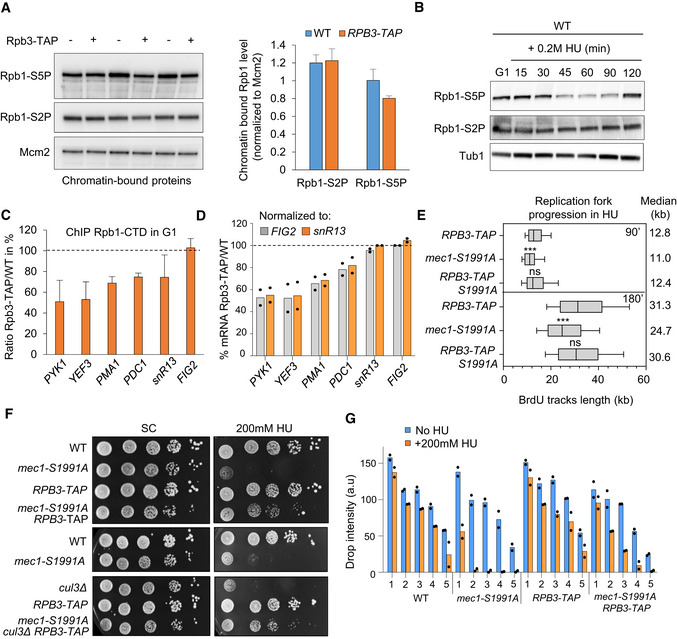
Decreased chromatin‐bound RNAPII levels triggered by *RBP3‐TAP* rescue *mec1‐S1991A* defects on HU APreparations of chromatin‐bound proteins were prepared in triplicate and subjected to SDS–PAGE and immunoblotting with the indicated antibodies in a strain that either does or does not express the Rpb3‐TAP protein from its endogenous locus (Table EV1). Quantification of chromatin‐bound Rpb1‐S2P and Rpb1‐S5P is shown on the right. Mcm2 is used as a loading control. SEM is indicated (*n* = 3 biological replicates).BG_1_ synchronized cells were released into S phase with 0.2 M HU, and total protein extracts were collected at the indicated time points (in min); samples were subjected to SDS–PAGE followed by immunoblotting with antibodies against Rpb1‐S2P, Rpb1‐S5P, or tubulin.CEnrichment of the RNAPII subunit Rpb1 on chromatin in G_1_ synchronized cells was assessed by ChIP‐qPCR at indicated genes. Rpb1 ChIP signal from Rpb3‐TAP expressing cells was normalized to the corresponding untagged strain. The dashed line indicates the amount of Rpb1 in the untagged strain. SEM (*n* = 3 biological replicates) is indicated. *FIG2* is a mating pheromone‐induced gene used as a control to compare inducible gene and constitutively expressed genes (*PYK1*, *YEF3*, *PMA1*, *PDC1,* and *snR13*).DmRNA levels measured by RT–qPCR in G_1_ synchronized cells in the indicated strains. Expression is normalized to either *snR13* or *FIG2*. Data are expressed as a ratio of Rpb3‐TAP/WT in percentage. Individual data points are indicated (*n* = 2 biological replicates).EAnalysis of replication fork progression (BrdU track lengths) at the single‐molecule level by DNA combing in the indicated strains. Box, whiskers, and median as in Fig 3D. ***P‐value < 10^−3^; ns = *P*‐value > 0.05, by Mann–Whitney rank‐sum test using *RPB3‐TAP* as a reference. *RPB3‐TAP* 90 min (*n* = 367) and 180 min (*n* = 490), *mec1‐S1991A* 90 min (*n* = 309) and 180 min (*n* = 477), *mec1‐S1991A RPB3‐TAP* 90 min (*n* = 490) and 180 min (*n* = 691).F, GA 10‐fold dilution series of cells from exponential SC cultures of the indicated strains were spotted on SC +/− 200 mM of HU. A high level of HU was used to be able to demonstrate robust suppression of the *mec1‐S1991A* phenotype. (G) Histogram presents quantification of two independent HU sensitivity assays with mean and individual data point values indicated for each yeast dilution. Source data are available online for this figure.

If Mec1‐S1991 phosphorylation contributes to RNAPII removal from chromatin during HU‐induced replicative stress to minimize impediments to fork progression, destabilization of RNAPII should reduce the sensitivity of *mec1‐S1991A* cells to HU. Indeed, earlier work suggests that inhibiting transcription can alleviate replication stress and facilitate DNA replication (Herrera‐Moyano *et al*, 2014; Chang *et al*, 2019). To reduce the amount of engaged Rpb1, we used a C‐terminal TAP fusion to Rpb3, a subunit of the holoenzyme that is known to stabilize RNAPII on DNA. The *RPB3‐TAP* allele does not alter the global chromatin‐bound level of elongating RNA polymerase (Ser2P) but reduces the level of promoter proximal bound RNA polymerase by about 20% (Ser5P; Fig 6A). We note that the Ser5P Rpb1 is indeed the form of RNAPII that is degraded during HU stress (Fig 6B). Consistently, the *RPB3‐TAP* allele reduces the amount of chromatin‐bound Rpb1 to ~50 to 75% of wild‐type levels in the promoter region of several highly transcribed loci, including *PYK1*, *YEF3*, *PMA1, snR13,* and *PDC1,* albeit not at *FIG2,* a control for a gene which is induced by pheromone synchronization (Fig 6C). Rpb1 reduction at those loci correlates with a decrease in the corresponding mRNA steady‐state levels in the *RPB3‐TAP* background (Fig 6D).

**Figure EV3:**
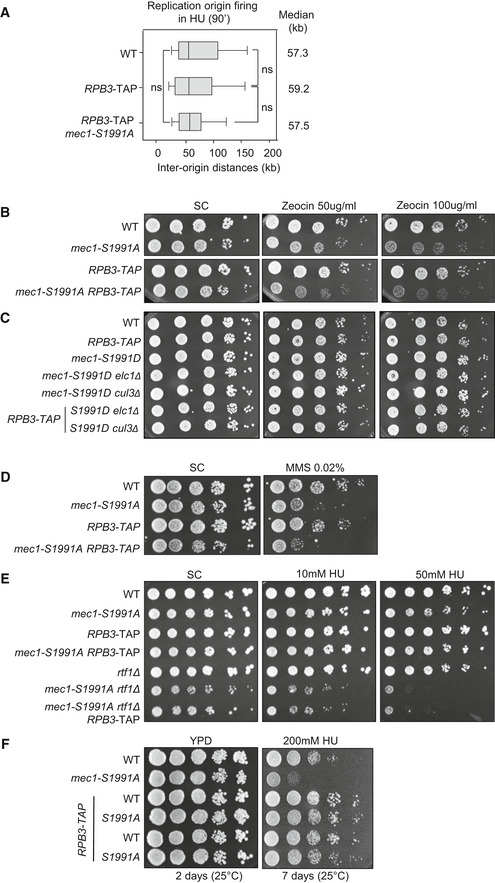
Rescue of *mec1‐S1991A* growth defect on HU by RBP3‐TAP requires the PAF1 complex subunit Rtf1 AAnalysis of inter‐origin distances at the single‐molecule level by DNA combing in the indicated strains. Graph depicting the distribution of inter‐origin distances determined by DNA combing after 90 min in S phase + 0.2 M HU. Box, 25–75 percentile range. Whiskers, 10–90 percentiles range. Median is indicated in kb. ns (*P*‐value > 0.01), by Mann–Whitney rank‐sum test. WT (*n* = 173), *RPB3‐TAP* (*n* = 89), and *mec1‐S1991A RPB3‐TAP* (*n* = 118). The WT data set is from Fig 3 and is included for comparison.B–FDrop assay showing a 10‐fold dilution series of cells from exponential SC cultures of the indicated strains that were spotted on SC +/− the indicated drug. *RTF1* encodes an essential subunit of the PAF1 complex. (E) In addition to rescuing *mec1‐S1991A* phenotype, expression of Rpb3‐TAP slightly improves WT resistance to high doses of HU on YPD. Source data are available online for this figure.

To see whether the decrease in chromatin‐bound RNAPII found in the *RPB3‐TAP* strain rescues the DNA replication defect of *mec1‐S1991A* on HU, we performed DNA combing in a *mec1‐S1991A RPB3‐TAP* strain. We found that replication forks progressed at the same rate in *mec1‐S1991A RPB3‐TAP* and in *RPB3‐TAP* cells in both early (90 min in HU; 12.4 vs 12.8 kb; Fig 6E) and mid‐S phase (180 min in HU; 30.6 vs 31.3 kb; Fig 6E), rescuing the slow fork phenotype observed earlier in the *mec1‐1991A* mutant (11.0 and 24.7 kb, respectively; Figs 3D and 6E). The presence of *RPB3‐TAP* did not affect origin firing in either wild‐type or *mec1‐S1991A* backgrounds (inter‐origin distances remain 59.2 and 57.5 kb, respectively, vs 57.3 kb in wild‐type; Fig EV3A). This excludes extra‐origin firing as a rescue mechanism. Strikingly, *RPB3‐TAP* also rescued the *mec1‐S1991A* sensitivity to chronic HU exposure on plates (Fig 6F and G) but did not impact growth when cells are exposed to Zeocin (Fig EV3B and C) or to MMS (Fig EV3D), while the *RPB3‐TAP* strain in a *MEC1* ^+^ background grew exactly like wild‐type both with and without DNA damage (Figs 6F and G, and EV3B–E).

The destabilization of RNAPII by *RPB3‐TAP* did not require a functional Cul3 ubiquitin ligase as *RPB3‐TAP* was able to suppress the HU sensitivity of the *mec1‐S1991A cul3Δ* double mutant (Fig 6F). On the other hand, the loss of PAF1 complex function through the deletion of *rtf1* attenuated the suppression by *RBP3‐TAP*, suggesting that the PAF1 complex functions in the same pathway (Fig EV3E). This is not surprising given that Paf1 itself interacts with RNAPII (Mueller & Jaehning, 2002) and possibly with RNAPIII (Bhalla *et al*, 2019). Interestingly, during prolonged growth on high levels of HU (0.2 M), we also see *mec1‐S1991A* sensitivity to HU on rich media (YPAD) and its suppression by *RPB3‐TAP* (Fig EV3F), ruling out media conditions as a factor in this suppression pathway. We conclude that the destabilization of RNAPII through modification of Rbp3 by a C‐terminal tag reduces steady‐state RNAPII engagement on chromatin, and this alone is sufficient to compensate for the HU sensitivity of the *mec1‐S1991A* mutation.

### The *mec1‐S1991A* allele alters the kinome response to HU‐induced replication stress

**Figure EV4:**
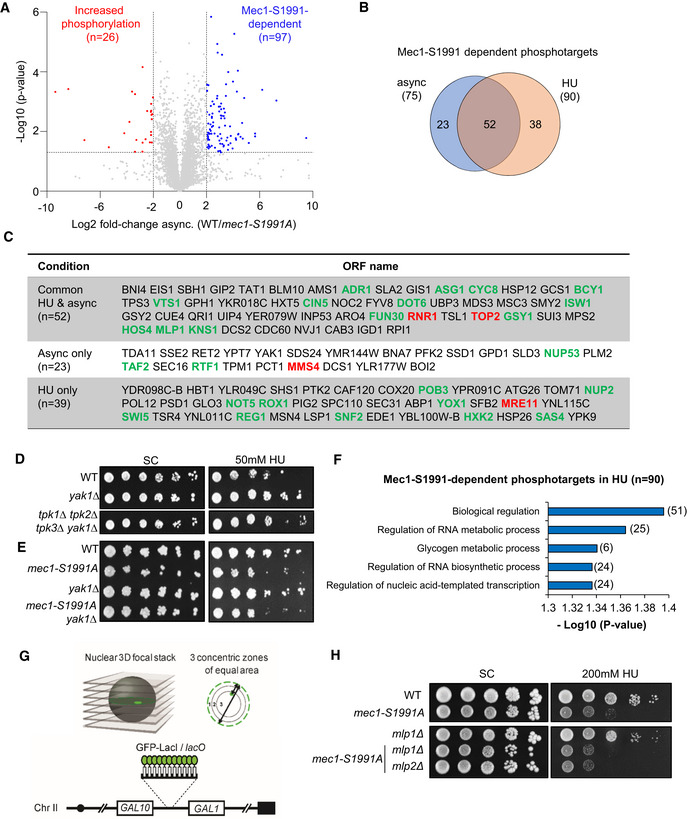
The *mec1‐S1991A* allele alters the kinome response to HU‐induced replication stress APhosphopeptide abundances (log_2_ ratio [WT/*mec1‐S1991A*]) in asynchronous cells. Colored dots are factors with significantly different phosphopeptide scores (WT/*mec1‐S1991A*) > 4 (blue) or < 4 (red) and *P*‐value < 0.05 (Student's paired *t*‐test, biological replicates *n* = 3; significant phosphopeptides *n* = 97 and *n* = 26, respectively). Full list in Dataset EV4.BVenn diagram showing the overlap of *mec1‐S1991A*‐dependent phosphotargets between asynchronous (*n* = 75) and HU‐treated (*n* = 90) cells.CMec1‐S1991‐dependent phosphotargets in asynchronous, HU‐treated cells or common between both conditions.D, EDrop assay showing a 10‐fold dilution series of cells from exponential SC cultures of the indicated strains that were spotted on SC +/− 50 mM HU.F
*mec1‐S1991A*‐dependent phosphotargets (*n* = 90) Gene Ontology on HU‐treated cells.GLacO repeats were inserted upstream of *GAL1* in order to visualize the locus in the presence of LacI‐GFP. Three zones of equal volume were defined based on erosion from the nuclear pore‐tagged ring. Zone 1 corresponds to the nuclear periphery and zone 3 to the center of the nucleus.HDrop assay showing a 10‐fold dilution series of cells from exponential SC cultures of the indicated strains that were spotted on SC +/− 0.2 M HU. *MLP1* and *MLP2* encode subunits of the inner nuclear pore basket that are implicated in the binding of highly transcribed genes. Source data are available online for this figure.

**Figure 7:**
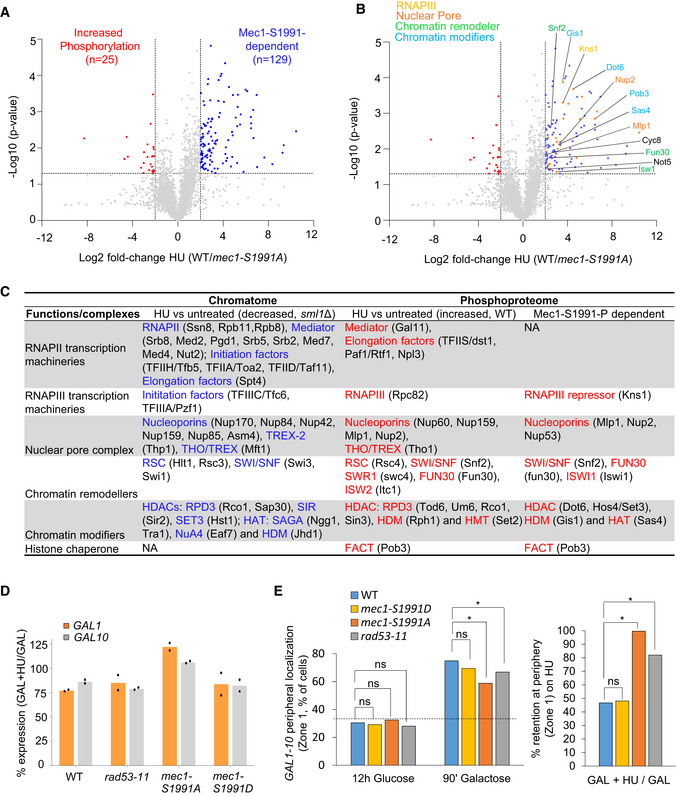
Mec1‐S1991 phosphorylation controls Mec1 kinase activity on HU APhosphopeptide abundances (log_2_ ratio [WT/*mec1‐S1991A*]) in S phase cells treated with 0.2 M HU for 60 min. Colored dots are factors with a significantly different phosphopeptide fulfilling a combined criteria (WT/*mec1‐S1991A*) > 4 (blue) or < 4 (red) and *P*‐value < 0.05 (Student's paired *t*‐test, biological replicates *n* = 3) in HU‐treated cells (significant phosphopeptides, *n* = 25 and *n* = 129, respectively).BAmong the Mec1‐S1991‐dependent phosphopeptides on HU, factors involved in transcription are highlighted. Full list (*n* = 25) in Dataset EV4.CPhosphotargets and subunits with decreased abundance on chromatin identified under HU‐induced replicative stress from the chromatome (Fig 1A and B) and the phosphoproteome analyses (Figs 1C and D, and 7A and B). Common complexes are indicated in blue or red.D
*GAL1* and *GAL10* mRNA levels measured by RT–qPCR in asynchronous culture in 2% galactose and after 90 min on HU in the indicated strains. Expression is normalized to *ACT1*. Repression is expressed as a ratio of HU‐treated/asynchronous cells in percentage. Mean and individual data point (*n* = 2 biological replicates) are indicated.EPosition relative to the nuclear envelope (Zone 1, see Fig EV4F) of the *lacO‐*tagged *GAL1‐GAL10* locus in the indicated strains grown either 12 h on glucose, or 90‐min galactose or galactose + 0.2 M HU. The third panel shows the relative retention at the nuclear periphery (Zone 1) on galactose + HU. The number of cells is > 300 for each condition. *P‐value < 10^−3^, by two‐sided Fisher's exact test. ns, not significant (*P* > 0.01).

To identify targets of Mec1 phosphorylation that are altered by the S1991A mutation, we performed quantitative phosphoproteomic mass spectroscopy to find peptides that are differentially phosphorylated in the *mec1‐S1991A* mutant, either during asynchronous exponential growth or in response to HU‐induced replication stress. Although the coverage was deep, identifying 5,250 phosphopeptides in total, only 97 showed differential phosphorylation in *mec1‐S1991A* vs wild‐type during asynchronous growth (Fig EV4A) and 129 differed in the presence of HU (Fig 7A, Dataset EV4). Comparing Mec1‐S1991 phosphorylation‐dependent targets from unchallenged and HU‐treated cells, 52 modified proteins were common to the two conditions (Fig EV4B and C). Among the Mec1‐S1991 phosphorylation‐dependent targets were factors involved in DNA replication such as the ribonucleotide reductase subunit Rnr1 or the replication initiation factor Sld3 as well as subunits of complexes promoting tolerance to replication stress and/or DNA damage repair complexes as Top2 and Mre11 (Fig EV4C, labeled in red). In addition, we found two members of the serine–threonine protein kinase A (PKA) pathway (Bcy1 and Yak1). Since the PKA pathway is involved in the control of the G_1_/S transition, we checked whether a defective PKA pathway sensitizes cells to either chronic or acute HU exposure. This was not the case (Fig EV4D and E), nor did the loss of the PKA pathway sensitize or suppress the sensitivity of *mec1‐S1991A* to HU.

A GO analysis on the phosphorylated proteins sensitive to *mec1‐S1991A* showed no significant enrichment for a biological process in exponentially growing cells, but once again we found a significant enrichment for factors involved in DNA transcription control and RNA synthesis in the HU‐treated cells (Fig EV4F and Dataset EV6). Among the factors controlling transcription were chromatin remodeler subunits such as Isw1, Swi5, Snf2, and Fun30, regulators of RNAPII and RNAPIII transcription such as Dot6 and Cyc8, and two subunits of the nuclear pore complex Nup2 and Mlp1 (Figs 7B and EV4C marked in green, Dataset EV6). As summarized in Fig 7C, these showed strong overlap with the factors or complexes that were evicted from chromatin upon HU stress, suggesting that Mec1‐S1991 may regulate transcription through multiple parallel pathways, during replication stress. Particularly striking was the number of nuclear pore complex components, which we had previously shown to be conditionally lethal with *mec1‐100* on HU (Hustedt *et al*, 2015). These, together with remodeler subunits (INO80, SWI/SNF) and other components of the basal transcription machineries (FACT, mediator, and subunits of RNAPII and RNAPIII), are of particular interest (Fig 7C; (Hustedt *et al*, 2015).

### Mec1‐S1991 phosphorylation promotes gene release from nuclear pores on HU

Other studies have proposed that RNAPII is released from highly transcribed loci at the nuclear pore by the Rad53 kinase in order to reduce replication stress (Bermejo *et al*, 2011). We hypothesized that if the *mec1‐S1991A* mutant also fails to decrease RNAPII occupancy on highly transcribed genes in response to HU, we should find elevated levels of RNAPII on inducible genes such as the divergently transcribed *GAL1‐GAL10* locus in the *mec1* mutant. We scored mRNA levels during *GAL* gene transcription‐inducing conditions before and after HU treatment, and noticed a ~20% reduction of *GAL1*–*GAL10* mRNA levels in wild‐type cells after HU treatment (Fig 7D). The drop in expression was also detected in a *rad53* mutant, and in the *mec1‐S1991D* background, but we robustly detected elevated levels of *GAL1* and *GAL10* mRNAs during HU‐induced replication stress in the *mec1‐S1991A* mutant (Fig 7D). It is well established that the *GAL1‐GAL10* locus relocates to nuclear pore complexes (NPC) when transcription is induced (Casolari *et al*, 2004; Cabal *et al*, 2006; Taddei *et al*, 2006). Gene tethering at the NPC requires the transcription machinery as well as TREX2/SAGA transcription factors (Dieppois & Stutz, 2010), and the release active genes from the NPC on HU were reportedly triggered by Rad53 (Bermejo *et al*, 2011). Given that the *mec1‐S1991A* allele alters the phosphorylation of nuclear pore subunits Nup2 and Mlp1, which are implicated in gene tethering, and fails to remove RNAPII in response to HU, we examined whether *GAL1‐GAL10* release from NPC was impaired in HU‐stressed *mec1‐S1991A* cells.

Using LacO‐tagged *GAL1‐GAL10* locus that can be visualized by the binding of LacI‐GFP, we scored the proximity of the locus to GFP‐tagged pores (Nup49‐GFP; Fig EV4G). As expected, in wild‐type cells the induction of transcription by galactose led to a strong relocation of the *GAL1‐GAL10* locus to the NPC (75% zone 1; Fig 7E) which was partially compromised in *rad53‐11* and *mec1‐S1991A* mutants. The addition of HU led to the release of the activated *GAL* locus from the NPC (54% release) in wild‐type and *mec1‐S1991D* cells (Fig 7E). However, *rad53‐11* and *mec1‐S1991A* alleles both impaired release of the *GAL* locus from the NPC on HU (Fig 7E). In the case of Rad53, this is thought to reflect a modification of pore proteins Mlp1 or Mlp2 by Rad53, since the deletion of these genes suppressed phenotypes associated with the *rad53* kinase‐deficient mutant *rad53‐K227A* (Bermejo *et al*, 2011). The HU sensitivity of the *mec1‐S1991A* allele, however, was unaffected by *mlp1* or *mlp2* deletion (Fig EV4H). Moreover, whereas HU led to a reduction of transcription in the *rad53‐11* mutant, the *mec1‐S1991A* mutant retained high RNAPII transcription levels. Thus, once again, a failure to shutdown RNAPII transcription in the *mec1‐S1991A* mutant accounts for retention of the *GAL* genes at the NPC on HU. This is consistent with extensive data showing that transcriptional activity/transcript processing factors (SAGA/Tho‐Trex2) mediate NPC tethering (Dieppois *et al*, 2006; Raices & D'Angelo, 2017).

### DNA replication reduces RNAPII occupancy in a Mec1‐dependent manner

**Figure 8:**
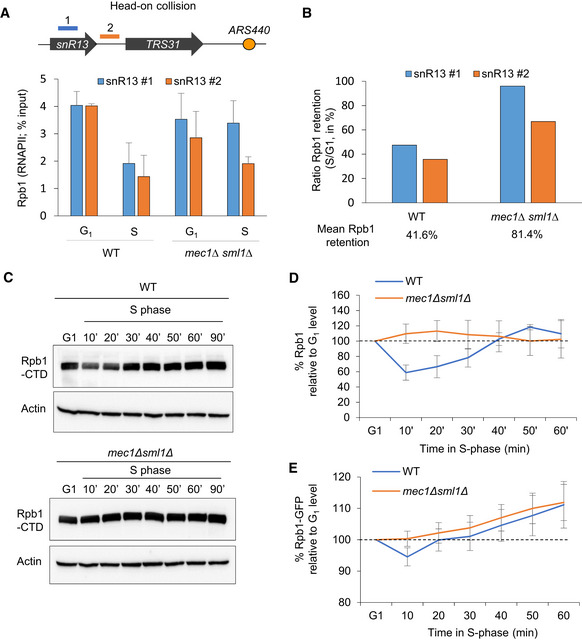
Mec1‐dependent RNAPII degradation occurs during an unchallenged S phase A, BRNAPII occupancy on chromatin in S phase was assessed by ChIP‐qPCR. (A) The level of Rpb1 was measured at *snR13‐TRS31* in both G_1_ and S phases (70 min at 16°C). Data are expressed as a percentage of input. SEM (*n* = 3 biological replicates) is indicated. (B) Quantification of the data from (A) depicting the mean retention of Rpb1 in S phase over G_1_.C, DTotal Rpb1 levels of exponentially growing cells that were synchronized in G1 with α‐factor and released into S phase at 25°C. Actin was used as a loading control. (D) Quantitation of blots in C. Rpb1 levels in S phase is expressed as a percentage of the starting level in G_1_ (100%, black dashed line). SEM for biological replicates (*n* = 4 for WT, *n* = 2 for *mec1Δsml1Δ*) is indicated.EQuantitation of Rpb1‐GFP fluorescent intensity expressed as percentage over the G_1_ level. SEM is indicated (*n* = 106 for WT and *n* = 102 for *mec1Δsml1Δ*). Source data are available online for this figure.

**Figure EV5:**
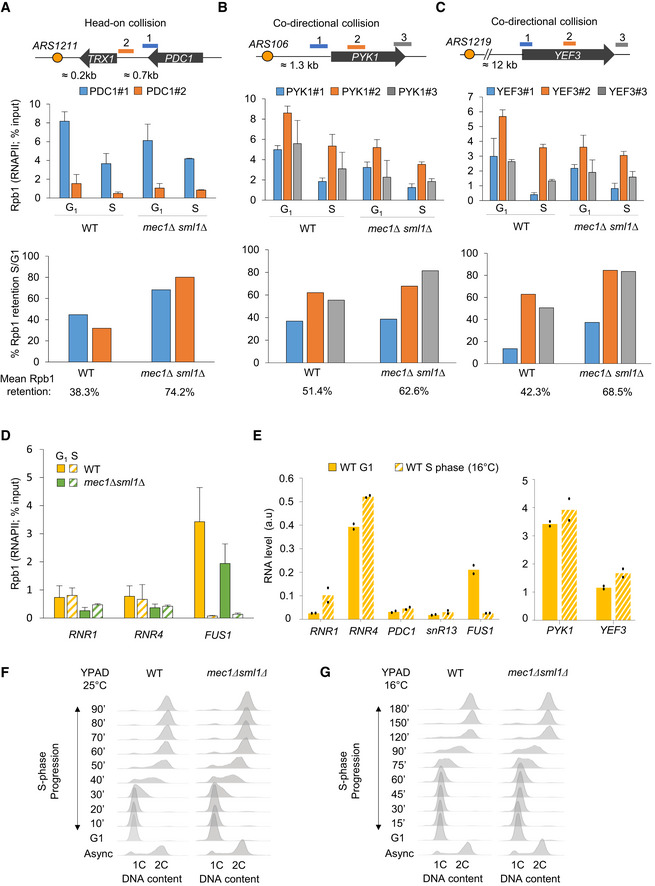
Unchallenged DNA replication transiently reduces RNAPII chromatin binding in a Mec1‐dependent manner A–CRNAPII retention on chromatin in normal S phase was assessed by ChIP‐qPCR by measuring the level of Rpb1 both in G_1_ and after 70 min in S phase at 16°C in wild‐type and *mec1∆sml1∆* cells. Rpb1 enrichment was quantified at loci known to generate transcription‐replication conflicts: *PDC1* (A), *PYK1* (B), and *YEF3* (C). The mean percentage of Rpb1 kept on chromatin in S phase is indicated as a ratio of the S phase/G_1_ level. Data are expressed as percentage of input. SEM (*n* = 3 biological replicates) is indicated.DRNAPII level on chromatin was assessed by ChIP‐qPCR by measuring the level of Rpb1 both in G_1_ and after 70 min in S phase at 16°C in wild‐type and *mec1∆sml1∆* cells. Data are expressed as percentage of input. SEM (*n* = 3 biological replicates) is indicated.EmRNA level measured by RT–qPCR in G_1_ phase and after 70 min in S phase for several loci in wild‐type cells. Expression levels are normalized to *ACT1*. Mean and individual data point values (*n* = 2 biological replicates) are indicated.F, GFlow‐cytometry analysis of DNA content. Asynchronous cells were synchronized in G_1_ with α‐factor and released into S phase at 25°C (F) or 16°C (G) in YPAD. The kinetics of G_1_/S transition as well as progression through S phase are shown over time.

The drop in chromatin‐bound transcription machinery and other transcription‐related factors during HU‐induced replication stress is thought to prevent replication fork stalling on the DNA template. However, interference between the two machineries can also occur during a regular S phase. It was recently proposed that chromatin‐bound RNAPII levels rapidly drop on the early replicated genes as soon as cells enter into S phase, in order to maintain a constant level of mRNA despite the duplication of the template (Bar‐Ziv *et al*, 2020). To test whether RNAPII occupancy or stability changes in an unperturbed S phase, we monitored the RNAPII largest subunit Rpb1 on chromatin by ChIP‐qPCR in wild‐type cells synchronized in G_1_ and released into S phase at 16°C. At this temperature, the kinetics of DNA replication is similar to replication at 25°C in the presence of 0.2 M HU (Cobb *et al*, 2003; Tittel‐Elmer *et al*, 2012). At a number of origin‐proximal genes, we detect a 50 to 60% drop in Rpb1 occupancy, during the unperturbed entry into S phase (Figs 8A and B, and EV5A–C). This is true for genes oriented such that transcription and replication machineries might collide head‐on as well as those co‐directionally transcribed (compare *snR13* and *PDC1* with *PYK1* and *YEF3* (Figs 8A and B, and EV5A–C).

During HU‐induced replication stress, Rpb1 removal from chromatin depends on the proteasome and reflects a Cul3‐mediated degradation of Rbp1 (Poli *et al*, 2016) (Fig 5C and D). To see whether Rbp1 is also degraded during an unchallenged S phase, and not simply displaced from chromatin, we monitored total Rpb1 protein level in a time‐course experiment using wild‐type cells synchronized in G_1_ and released into S phase. Total level of Rpb1 decreased to ~70% of the initial G_1_ level amount and was restored as cells finished replication by 40–50 min (Fig 8C and D and Appendix Fig S3A). To a lesser extent, we could confirm the reduction of Rpb1 level very early in S phase by using live microscopy to measure Rpb1‐GFP in a similar time‐course experiment (Fig 8E).

To see whether the replication machinery has a direct effect on RNAPII binding, we first analyzed Rpb1 enrichment at the *RNR4* and *RNR1* genes, which are located far away from replication forks. These transcribed loci maintained constant Rbp1 levels in G_1_ and S phases, while mRNA levels increased (Fig EV5D and E). The overall abundance of moderate level transcripts (*PDC1, snR13*) and high level transcripts (*PYK1, YEF3*) showed no particular change between G_1_ and S phases, despite experiencing a drop in RNAPII occupancy (Fig EV5E). This is not an artifact of pheromone arrest and release, as we can measure the reduction of mRNA steady‐state levels at the α‐factor‐induced gene *FUS1* after release into S phase (Fig EV5D and E). We conclude that there is a drop in RNAPII levels during DNA replication, not only at origin‐proximal loci after HU‐arrest, but also during an unchallenged S phase.

The DNA replication checkpoint is known to be activated during replication stress, yet Mec1 is active and has unique sets of targets in early S phase, even in the absence of exogenous stress (BastosdeOliveira *et al*, 2015; Lanz *et al*, 2018; Forey *et al*, 2020). Given that the Mec1^ATR^ kinase limits TRC by reducing chromatin‐bound RNAPII on HU (Im *et al*, 2014; Lafon *et al*, 2015; Poli *et al*, 2016), we examined its role in this phenomenon in an unchallenged S phase. We scored G_1_ and early S phase Rpb1 levels by ChIP‐qPCR in a *mec1Δsml1Δ* strain and compared it to wild‐type levels. Although the loss of Mec1 did not impact cell cycle progression (Fig EV5F and G), we found that Rpb1 levels are lower at some genes in G_1_ phase *mec1Δsml1Δ* cells_,_ than in wild‐type cells (Fig EV5A–C). When normalized to G_1_ levels, we found a proportionately higher rate of Rpb1 retention in the gene bodies of loci prone to transcription‐replication conflicts (*PYK1*, *YEF3*, *PDC1,* and *snR13‐TRS31*), in the *mec1Δsml1Δ* mutant (Figs 8A and B, and EV5A–C). We then checked to see whether Mec1 is needed for Rpb1 degradation in an unchallenged S phase by monitoring total Rpb1 levels with either an Rpb1‐CTD antibody or a Rpb1‐GFP fusion protein in *mec1Δsml1Δ* cells. The mutant cells maintained Rpb1 levels through S phase at its initial G_1_ level, as monitored either by Western blot or by live microscopy (Fig 8C–E). Thus, we find a Mec1‐dependent decrease in both the total and the chromatin‐bound fractions of the large subunit of RNAPII during an unchallenged S phase, a phenomenon enhanced by checkpoint kinase hyperactivation on HU.

## Discussion

The replication and transcription machineries use the same DNA template in S phase, necessitating mechanisms that coordinate their activities and limit interference between the two processes. Under various forms of stress, unscheduled transcription and/or excessive origin firing can lead to a loss of coordination between RNA and DNA polymerases, thus enhancing the frequency of transcription–replication conflicts (Macheret & Halazonetis, 2018). In this context, the DNA replication checkpoint cascade, and particularly its upstream kinase Mec1^ATR^, is of paramount importance to resolve transcription–replication conflicts by removing RNA polymerases from chromatin (Im *et al*, 2014; Poli *et al*, 2016; Landsverk *et al*, 2019). It has been unclear which signals activate Mec1 kinase's control over transcription nor was it known whether the attenuation of transcription occurs during an unchallenged S phase.

**Figure 9:**
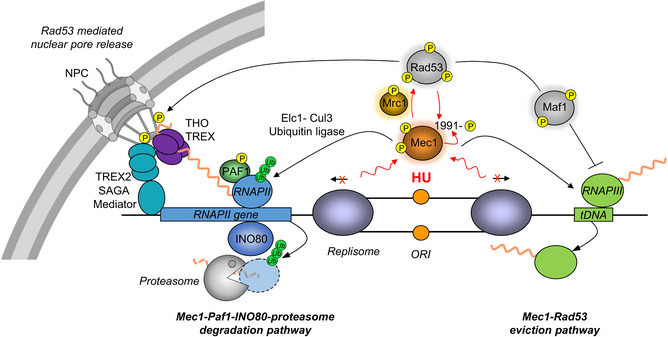
Mec1‐S1991 phosphorylation limits transcription during replication stress Upon HU‐induced replication stress, Mec1^ATR^ and Rad53 are activated through phosphorylation, including phosphorylation at Mec1‐S1991. Mec1‐S1991 phosphorylation is not required to activate the downstream checkpoint effector kinase Rad53, yet it promotes the attenuation of RNAPII‐ and RNAPIII‐mediated transcriptions during replication stress, acting on a variety of transcription controlling factors. Proteasome‐mediated RNAPII degradation during replication stress requires a functional Cul3‐Elc1 ubiquitin ligase. RNAPIII is evicted in a Mec1‐dependent manner but not degraded. Mec1‐induced RNAPII removal from chromatin allows the release of highly transcribed genes from nuclear pore under HU stress, while Rad53 is thought to act directly on the nuclear pore complex (NPC).

Here, we show that the phosphorylation of Ser1991 in the Mec1 kinase promotes the rapid degradation of RNAPII and eviction of transcription machineries in response to HU‐induced replication stress (see model in Fig 9). Loss of this phosphoacceptor site increased sensitivity to oxidizing agents and HU, despite normal Rad53 activation. The inability of the *mec1‐S1991A* mutant to shutdown transcription led to the persistent tethering of active genes at the nuclear pore complex even on HU (Fig 7), and the destabilization of chromatin‐bound RNAPII rescued *mec1‐S1991A* replication and growth defects on HU (Fig 6). This leads us to propose that the main function of Mec1‐S1991 phosphorylation is to reduce the level of engaged RNAPII transcription during replication stress.

We implicate Mec1‐S1991 phosphorylation both in RNAPII degradation and in RNAPIII eviction from chromatin in cells acutely exposed to HU (Fig 9). Highly transcribed tRNAs were among the first loci identified as polar blocks to DNA replication. Upon replication stress, the Mec1‐Rad53 checkpoint transiently reduces tRNA transcription by inhibiting RNAPIII transcription (Nguyen *et al*, 2010). We found that *mec1‐S1991A* partially mimics *mec1* or *rad53* null mutants with respect to RNAPIII, as it fails to decrease the transcription of 26% of yeast tRNAs during HU‐induced replication stress. A recent study showed that tRNA‐mediated fork stalling is linked to the presence of the RNAPIII initiation factor TFIIIB, rather than RNAPIII transcription machinery itself (Yeung & Smith, 2020). In agreement, we found that Mec1‐S1991 phosphorylation was implicated in the eviction of both the RNAPIII subunit Rpc82 and the TFIIIB subunit Brf1. Moreover, Rpc82 is shown to be a HU‐induced phosphotarget on chromatin (Fig 1).

Although the DNA replication checkpoint was shown to decrease tRNA transcription levels by activating the RNAPIII repressor Maf1 (Nguyen *et al*, 2010), the *mec1‐S1991A* mutant is proficient for HU‐induced Maf1 activation, and thus, it acts on another pathway, presumably in parallel to Rad53. Interestingly, a recent study proposed that the RNAPII elongation complex PAF1 also associates with RNAPIII and facilitates its removal from chromatin during replication stress (Bhalla *et al*, 2019). Since the PAF1 complex is a direct target of Mec1 and is necessary for checkpoint‐induced RNAPII degradation from chromatin during replication stress, Mec1 and S1991 phosphorylation may also contribute to RNAPIII eviction by targeting this complex (Poli *et al*, 2016; Landsverk *et al*, 2019). Consistently, we showed that *mec1‐S1991A* alters the phosphorylation of the PAF1 subunit Rtf1 (Fig EV4C). Our data are thus consistent with a model in which the PAF1 complex promotes both RNAPII and RNAPIII removal from chromatin in response to Mec1 signaling during replication stress.

The Mec1^ATR^‐dependent degradation of the large RNAPII subunit Rpb1 on HU is reminiscent of the UV‐induced degradation of stalled RNAPII (García‐Muse & Aguilera, 2016; Poli *et al*, 2016; Landsverk *et al*, 2020). In this latter pathway, Def1 promotes Rpb1 poly‐ubiquitination by the E3 ligase Cul3‐Elc1‐Ela1, leading to Cdc48‐assisted proteasomal degradation (Verma *et al*, 2011; Wilson *et al*, 2013). During replication stress, Rpb1 is also poly‐ubiquitinylated and degraded in a Cdc48‐dependent manner (Lafon *et al*, 2015). Our results indicate that the inactivation of the Cul3‐Elc1 ubiquitin ligase sensitized cells to constitutive HU‐induced replication stress and prevented Rpb1 degradation on acute HU treatment. In addition, we found that *mec1‐S1991A* was epistatic with *cul3Δ* or *elc1Δ*. That is, single and double mutants had identical survival rates in HU. Moreover, in human cells Cul3‐Elc1 interacts with the PAF1 complex and travels with the RNAPII transcription machinery (preprint: Sanchez *et al*, 2020). These data suggest that Mec1 likely acts through Cul3‐Elc1 to induce RNAPII degradation during replication stress.

We have shown that Mec1‐S1991 phosphorylation contributes to RNAPII degradation and RNAPIII removal from chromatin during HU‐induced stress and reduces RNA:DNA hybrid accumulation at tRNA loci and RNAPII genes. However, it was unclear whether or not RNA:DNA hybrids are responsible for the growth and replication defects in cells exposed to HU, or rather result from the failure to evict or degrade RNA polymerases. To determine whether Mec1‐S1991 phosphorylation specifically acts on these structures, we tried to reduce R‐loop accumulation in the *mec1‐S1991A* strain by overexpressing RNaseH genes. An analysis of the growth defect in the presence of HU in strains with either increased (*mec1‐S1991A* + pGAL::RNH1 or pGAL::RNH201) or decreased (*mec1‐S1991A rnh1Δrnh201Δ*) RNaseH activity did not substantially alter growth on HU (Appendix Fig S4A,B). While the absence of an effect is inconclusive, we favor the model that RNA:DNA hybrids likely result from a failure to shutdown transcription on HU and that this is the ultimate cause of the HU sensitivity of the *mec1‐S1991A* mutant. We note that by decreasing the occupancy of chromatin‐bound RNAPII through a Rpb3 fusion (*RPB3‐Tap*), we were able to restore normal growth and fork progression under HU‐induced replicative stress in the *mec1‐S1991A* mutant. The destabilized RNAPII also compensated for the loss of the E3 ligase Cul3, which targets RNAPII. Thus, our data argue that a failure to remove the RNAPII transcription machinery constitutes the major impediment for replication in the absence of Mec1‐S1991 phosphorylation.

Studies of the mammalian Mec1 homologue ATR have described an autophosphorylation site at a very similar location, ATR‐T1989, which serves as an internal activator of the kinase (Liu *et al,* 2011; Nam *et al,* 2011). Like Mec1‐S1991, ATR‐T1989 is located upstream of the kinase domain and stimulates its activity, facilitating survival of UV and/or replicative stress (Liu *et al*, 2011). Although ATR‐1989 is a *bona fide* ATR autophosphorylation site, Mec1‐S1991 phosphorylation requires the presence of both Mec1 and Rad53 kinases (Hustedt *et al*, 2015). This does not rule out autophosphorylation, yet it suggests a more complicated pathway of control. Unfortunately, we were unable to reconstitute Mec1 phosphorylation activity *in vitro*, to modify S1991 on recombinant protein. Since both Mec1 and ATR promote RNAPII removal from chromatin during replication stress (Im *et al*, 2014; Poli *et al*, 2016), it will be intriguing to assess the functional conservation of mammalian ATR‐T1989 phosphorylation with respect to Mec1‐S1991 in controlling transcription levels during replication stress. We note that in mammalian cells, the cleavage of R‐loop‐stalled forks also facilitates RNAPII passage (Chappidi *et al*, 2020).

The comparison of chromatin landscape at a single locus in cells with or without HU showed a drastic rewiring of proteins associated with chromatin, marked by a strong decrease in the transcription machinery (Korthout *et al*, 2018). Our chromatome results confirmed and extended these results genome‐wide. In addition to RNAPII and RNAPIII subunits, we found several subunits of the Mediator complex as well as key components of the inner basket of the nuclear pore depleted from chromatin on HU, which correlated with the detection of Mec1‐S1991‐dependent HU‐induced phosphopeptides on nucleoporins and chromatin remodelers (Fig 7C). We propose that the phosphorylation of these complexes contributes to the global transcriptional shutdown suggested by our chromatome analysis. However, we suggest that the activated Mec1‐S1991p enzyme acts on a variety of chromatin remodelers and/or transcriptional modifiers, and not on a single pathway of RNAPII or RNAPIII control. Previous studies support the notion that Mec1 controls chromatin accessibility at stalled replication forks in yeast by acting through multiple remodeler activities (Shimada *et al*, 2008; Rodriguez & Tsukiyama, 2013). Evidence for physical interactions with remodelers or the degradation machinery requires additional study.

Finally, we found that the large RNAPII subunit Rpb1 is also transiently and rapidly degraded in a Mec1‐dependent manner when cells enter an unperturbed S phase. This is consistent with previous quantitative mass spectrometry observations showing that Mec1 is functional during unperturbed S phase (BastosdeOliveira *et al*, 2015; Lanz *et al*, 2018). We find that a decrease in the level of chromatin‐bound RNAPII also improved tolerance/growth of wild‐type cells to HU‐induced replicative stress (Fig EV3F). Other studies have proposed that during replication, RNAPII occupancy is reduced on all genes to ensure that mRNA levels do not double when a transcribed gene is replicated (Voichek *et al*, 2018; Bar‐Ziv *et al*, 2020). However, RNAPII downregulation in their studies was not Mec1‐dependent. The Mec1‐dependent decrease in RNAPII described here appears to stem from global degradation or reduction in RNAPII levels (Fig 8), yet it is possible that there is also a Mec1‐independent pathway that works in parallel to ensure reduced RNAPII occupancy in S phase. Our observations and those of Voichek *et al* (2018) are therefore neither contradictory nor mutually exclusive, but reinforce the notion that it is crucial to tightly regulate transcription in S phase cells (Fig 9). Checkpoint kinase‐mediated transcriptional control is likely to be conserved in human cells undergoing replication, given recent evidence that basal activity of the ATR kinase is required to engage an immediate response following stalling of RNAPII machinery by CDK9 inhibition (Shao *et al*, 2020).

## Materials and Methods

### Yeast strains, culture conditions, drop assay, and flow cytometry

All strains used are listed in Table EV1. For liquid cultures, synthetic complete medium was supplemented with 2% glucose unless otherwise stated. *MAT*a cells were synchronized in G_1_ by adding α‐factor (5 μg/ml, Biotem, No.2968) for 170 min at 25°C unless otherwise stated. Arrest without buds was monitored by phase microscopy. G_1_‐blocked cells were released into S phase by washing or by the addition of 75 μg/ml Pronase and were treated or not with 0.2 M HU (US Biologicals, H9120). Flow‐cytometry samples were prepared as previously described (Poli *et al*, 2016). Data were acquired on a FACSCalibur (Becton Dickinson) and analyzed with FlowJo. Drop assays were done with exponentially growing cells adjusted to 1·10^7^ cells/ml. 10‐ or 5‐fold serial dilutions, as indicated, were spotted on YPAD or SC plates +/− the indicated drug. To quantify cell growth from drop tests, the round box tool from ImageJ (Fiji) software was used to select the five drops corresponding to each strain and determined the mean gray intensity value. When background was similar, raw data values were directly used to generate histograms; otherwise, values were normalized on background levels.

### Protein extracts, chromatin fractionation, and Western blotting

Total protein extracts and chromatin fractionation were performed as previously described (Poli *et al*, 2016). Proteins were resolved by SDS–PAGE and transferred with a Trans‐Blot (Bio‐Rad). After blocking, proteins were either probed with anti‐RNAPII CTD (Abcam 8WG16, ab817), anti‐Rpb1‐S5P (Clone 3E8, Merck, 04‐1572), anti‐Rpb1‐S2P (Abcam, ab5095), anti‐PK for Maf1‐3PK strains (Novus Biologicals, NB600‐381), anti‐Rad53 (clone 11G3G6, custom made by GenScript), anti‐Mcm2 (N‐19, Santa Cruz, sc‐9839), anti‐tubulin (Thermo Fisher Scientific, MA1‐80017), or anti‐actin (clone C4, Sigma‐Aldrich, MAB1501). Blots were scanned with an ImageQuant LAS4000 mini (GE Healthcare), and semi‐quantitative determination of protein level was performed using the ImageJ (Fiji) software using tubulin, actin, or Mcm2 for normalization.

### RNAPII and RNAPIII chromatin immunoprecipitation

ChIP‐qPCR was performed as described in Poli *et al* (2016) using anti‐HA probe F‐7 (Santa Cruz, sc‐7392) and anti‐Rpb1‐CTD 8WG16 (Abcam, ab817) coupled to Dynabeads (Invitrogen, protein A and sheep anti‐mouse M280 IgG). For quantitative PCR, background controls were determined using uncoupled Dynabeads and enrichment was normalized to chromatin Input. Primers used for ChIP‐qPCR are listed in Table EV2.

### Genome‐wide replication timing analysis

Replication timing analysis was performed as previously described in Fang *et al* (2017). Genomic DNA was isolated using Qiagen genomic DNA extraction kit according to the manufacturer's instructions. DNA was fragmented using sonication (∼ 200‐ to 500‐base‐pair [bp] size range). Sequencing libraries were prepared using a Thru‐PLEX DNA‐seq kit (Rubicon Genomics) and sequenced on a HiSeq 4000 (Illumina). Single‐end reads of 50 bp were aligned to the *S*. *cerevisiae* genome (2011) with Bowtie, allowing only perfect matches. Relative copy number was determined as the ratio of normalized reads on HU and G_1_ cells.

### Pol2 (DNAPol ϵ) chromatin immunoprecipitation and sequencing

ChIP was performed as described by Gutin *et al* (2018), except that the on‐bead library preparation was substituted with on‐bead tagmentation as described by Schmidl *et al* (2015). Cells were collected 15, 60, 90, and 150 min after release in HU and cross‐linked with 1% formaldehyde. Chromatin was fragmented with a Bioruptor Plus (Diagenode) for 25 min (30‐s on, 30‐s off) at high intensity in a cooled water bath. Pol2‐HA was immunoprecipitated with 3 µg of HA antibody for 2.5 h at 4°C with gentle tumbling and recovered by 1‐h incubation with 20 µl protein G beads at 4°C. After washes, eluted chromatin was digested with 0.5 µg RNase A for 30 min at 37°C and 50 µg Proteinase K for 2 h at 37°C, and then de‐cross‐linked for 12–16 h at 65°C. DNA was isolated with 2.2X SPRI beads purification and amplified with KAPA Hifi Hotstart Ready Mix PCR (after pre‐activation at 98 for 3 min, 14 cycles) with barcoded Tn5 primers resulting in multiplexed libraries. Libraries were sequenced by an Illumina NovaSeq with 50 bp paired‐end sequencing.

### Pol2 (DNAPol ϵ) ChIP‐seq data processing

For ChIP‐Seq analysis, only unique read‐pairs were kept. Total coverage was normalized so that the mean coverage in non‐repeated regions of the genome was one, and all reads were subdivided into 200 bp bins. For Fig 3E, the median normalized bin occupancy for 30 kb around the 32 earliest ORIs (according to (Yabuki *et al*, 2002)) at each time point was plotted against the absolute distance, not distinguishing between up‐ and downstream sequences.

### RNA extraction, RT, and RNA‐seq

Total RNA was extracted using standard hot phenol procedure. RT–qPCR was performed from at least two independent biological replicates, starting with 3 µg of RNA. Strand‐specific total RNA‐seq libraries were prepared from rRNA‐depleted total RNA preparation with the TruSeq kit (Illumina) and sequenced by paired‐end 2 × 37 bp. tRNA expression analyses were done by allowing multiple mapped reads (100). Tag densities were normalized using RPKM. Primers used for RT‐qPCR are listed in Table EV2.

### DRIP‐qPCR

DRIP experiments were done as previously described (Lafuente‐Barquero *et al*, 2020) except for the following changes. After chloroform precipitation, DNA was recovered on a glass rod. DRIP was performed with 4.5 µg of DNA and 10 µl of S9.6 antibody (1 mg/ml, Antibodies Inc.) incubated overnight rotating at 4°. The DNA‐antibody mixture was incubated with Dynabeads M280 sheep anti‐mouse (Life Technologies) for 4 h at 4°C rotating. Beads were washed five times with binding buffer and DNA eluted in 120 μl elution buffer (50 mM Tris pH 8, 10 mM EDTA, 1% SDS) at 65°C for 10 min. Eluates were incubated 1 h with 10 μl proteinase K at 50°C and purified with the AccuPrep Clean‐up Purification Kit (Bioneer).

### DNA combing

DNA combing was performed as described (Bianco *et al*, 2012) using a mouse monoclonal anti‐ssDNA (Chemi‐Con, clones 16–19) and a rat monoclonal anti‐BrdU (Abcys, clone BU1/75). Images were recorded on a Zeiss Axio Imager Microscope equipped with a CoolSNAP HQ CCD camera (Roper Scientific) and were processed as described (Bianco *et al*, 2012).

### Fixed microscopy and image analysis

Fixed microscopy and image analysis including the calculation of foci in nuclear zones were done as in Horigome *et al* (2014) for at least 300 cells/condition.

### Live microscopy and image analysis

Live microscopy and image analysis were done as previously described (Poli *et al*, 2016) with the following changes. Yeasts were observed using a Zeiss Axio‐Observer Widefield Microscope equipped with a Hamamatsu ORCA Flash4 camera and a plan apochromat 63X NA = 1.4 oil objective and a XCite 120 LED fluorescence light source. Time‐lapse series (80 min in total) of 15 optical slices per stack of 0.4 µm were acquired every 10 min. After deconvolution, nuclei were detected and segmented using Imaris and a fixed threshold value. Each nucleus was tracked through the time series. The integrated nuclear intensity was then calculated for each cell nucleus.

### Mass spectrometric analysis of phosphopeptides

Using an exponentially growing culture (asynchronous) in SC medium, one half of the cells were arrested in G_1_ phase at 30°C using α factor (5 μg/ml, Biotem) and were washed once with SC, then released into SC medium containing 0.2 M HU for 60 min. Identification of phosphopeptides was done as described previously (Hustedt *et al*, 2015).

### Sucrose gradient enrichment for quantitative mass spectrometry analysis of chromatin

Two liters of *MATa* yeast culture were synchronized in G_1_ by adding α‐factor at 25°C. Arrest without buds was monitored by phase microscopy. G_1_ synchronized cells were released into S phase by washing and were treated or not with 0.2 M HU. Chromatin fractionation was performed as described previously (Challa *et al*, 2021).

### TMT‐mass spectrometry analysis

TMT‐mass spectrometry samples were prepared as described in Challa *et al* (2021) with minor modifications. Briefly, samples were prepared using PreOmics iST‐NHS kits (PreOmics, Martinsried, Germany) and TMTpro 16plex (tandem mass tag) reagents (Thermo Fisher Scientific) following a combination of both manufacturer's recommended protocols. The Orbitrap analyzer was used to record MS2 spectra at 50k resolution.

### Proteomic data analysis

Proteomic data analysis was done with Proteome Discoverer PD2.4 (Thermo Fisher Scientific) for every TMT multiplexing experiment individually before creating a multi‐consensus, according to Ginno *et al* (2018) with minor modification. See online Materials and Methods for details.

## Author contributions

VH, FJ, KC, NB, KS, SMG, and JP designed the experiments. VH, FJ, KC, KS, and JP performed the experiments. FJ and RF prepared the libraries, sequencing, and bioinformatic analyses. RS and JS acquired mass spectrometry data. RS, JS, and CDS analyzed the mass spec data. KS, SMG, and JP supervised and administrated the project. JP and VH wrote initial drafts of the manuscript. KS, SMG, and JP wrote, reviewed, and edited the manuscript.

## Conflict of interest

The authors declare that they have no conflict of interest.

## Supporting information



Appendix

Expanded View Figures PDF

Table EV1

Table EV2

Dataset EV1

Dataset EV2

Dataset EV3

Dataset EV4

Dataset EV5

Dataset EV6

Source Data for Expanded View

Source Data for Figure 2

Source Data for Figure 3

Source Data for Figure 5

Source Data for Figure 6

Source Data for Figure 8

## Data Availability

The datasets and computer code produced in this study are available in the following databases:
RNA‐seq data, replication forks progression, and replication timing data: Gene Expression Omnibus GSE180167 (https://www.ncbi.nlm.nih.gov/geo/query/acc.cgi?acc=GSE180167). The mass spectrometry proteomic data have been deposited to the ProteomeXchange Consortium via the PRIDE partner database with the accession code PXD027337 (Perez‐Riverol *et al*, 2019). (http://www.ebi.ac.uk/pride/archive/projects/PXD027337).
