# Supplementary material for: A regulatory phosphorylation site on Mec1 controls chromatin occupancy of RNA polymerases during replication stress
Source: EMBO J. 2021 Sep 27;40(21):e108439. doi: 10.15252/embj.2021108439 (PMC8561635; doi:10.15252/embj.2021108439)
Supplement: Supplementary file 1 — Appendix [file EMBJ-40-e108439-s010.pdf]

Appendix

Tables of contents:

Appendix Figures S1 to S4 and their legends

Appendix Methods

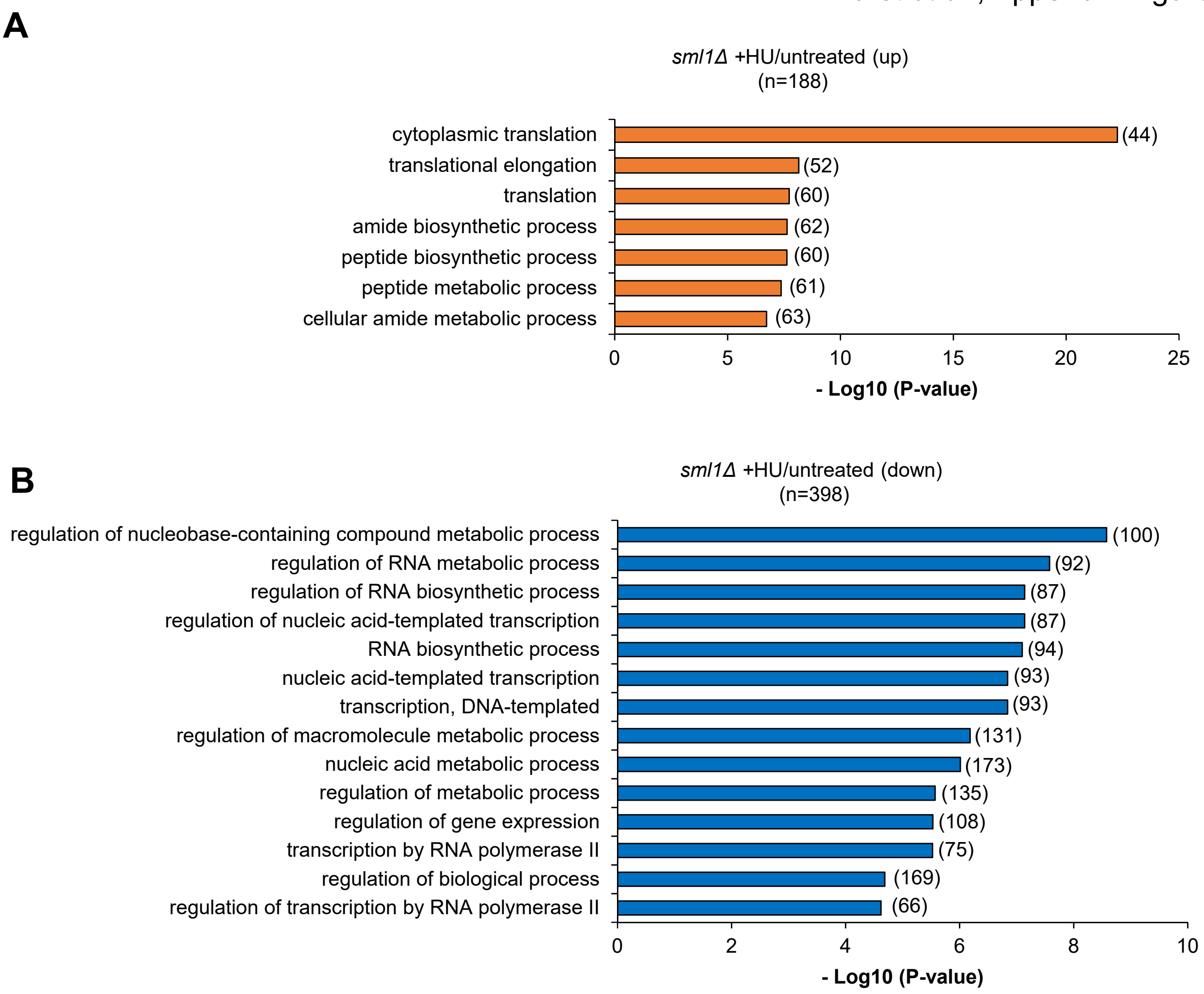

Appendix Figure S1: **Genome-wide changes in chromatin binding upon HU-induced replication stress**. (A-B) Gene ontology analysis on factors showing a significantly different chromatin binding score by comparing HU-treated/untreated in *sml1Δ* cells (p<0.1 and fold-change >|0.5|).

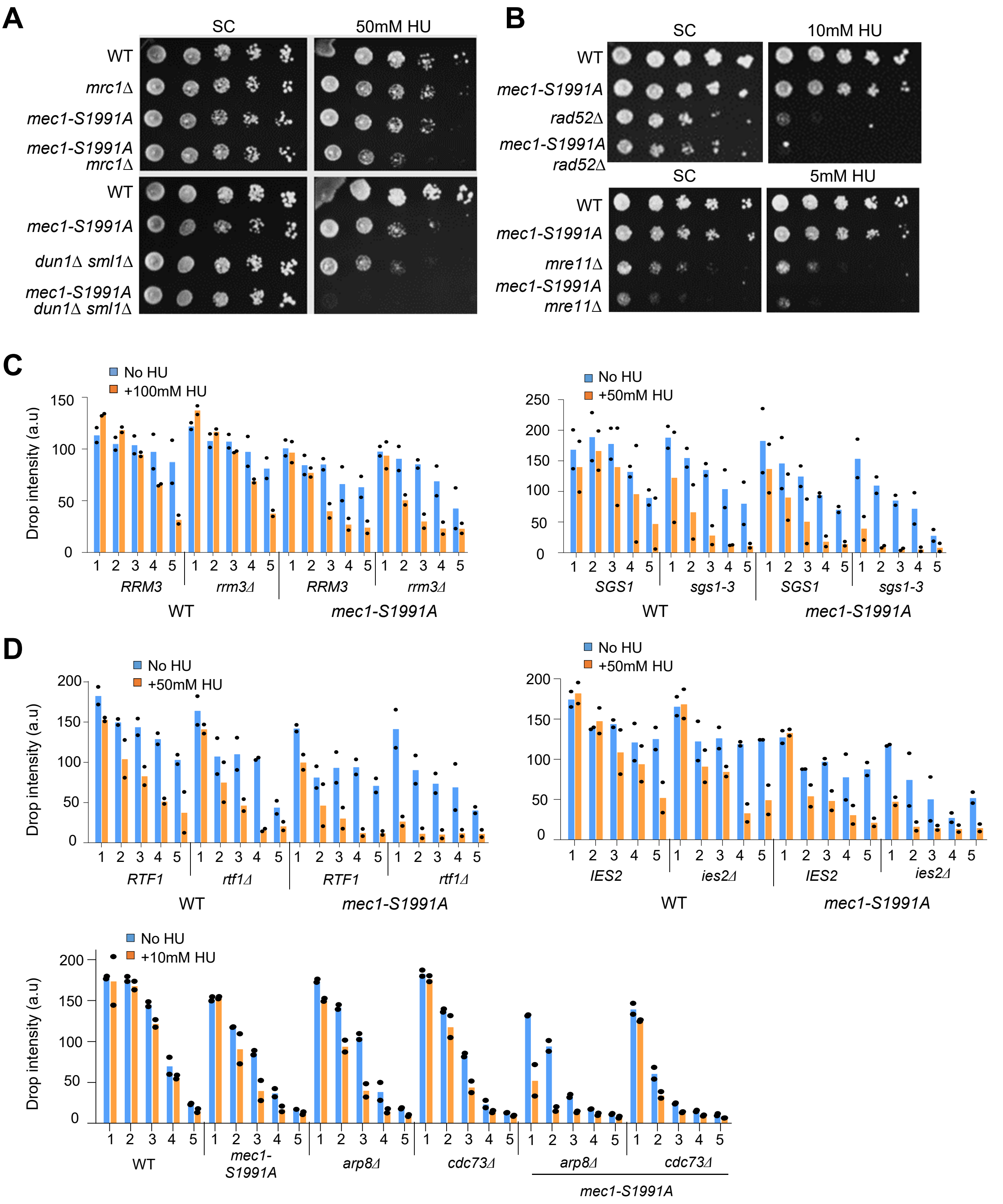

Appendix Figure S2: **Negative genetic interactions on HU in absence of Mec1-S1991 phosphorylation.** (A -B) 10-fold dilution series of cells from exponential SC cultures of the indicated strains spotted on SC +/- the indicated dose of HU. (C,D) Histograms present quantification of 2 independent HU sensitivity assays with mean and individual data point values which are indicated for each yeast dilution (1 to 5).

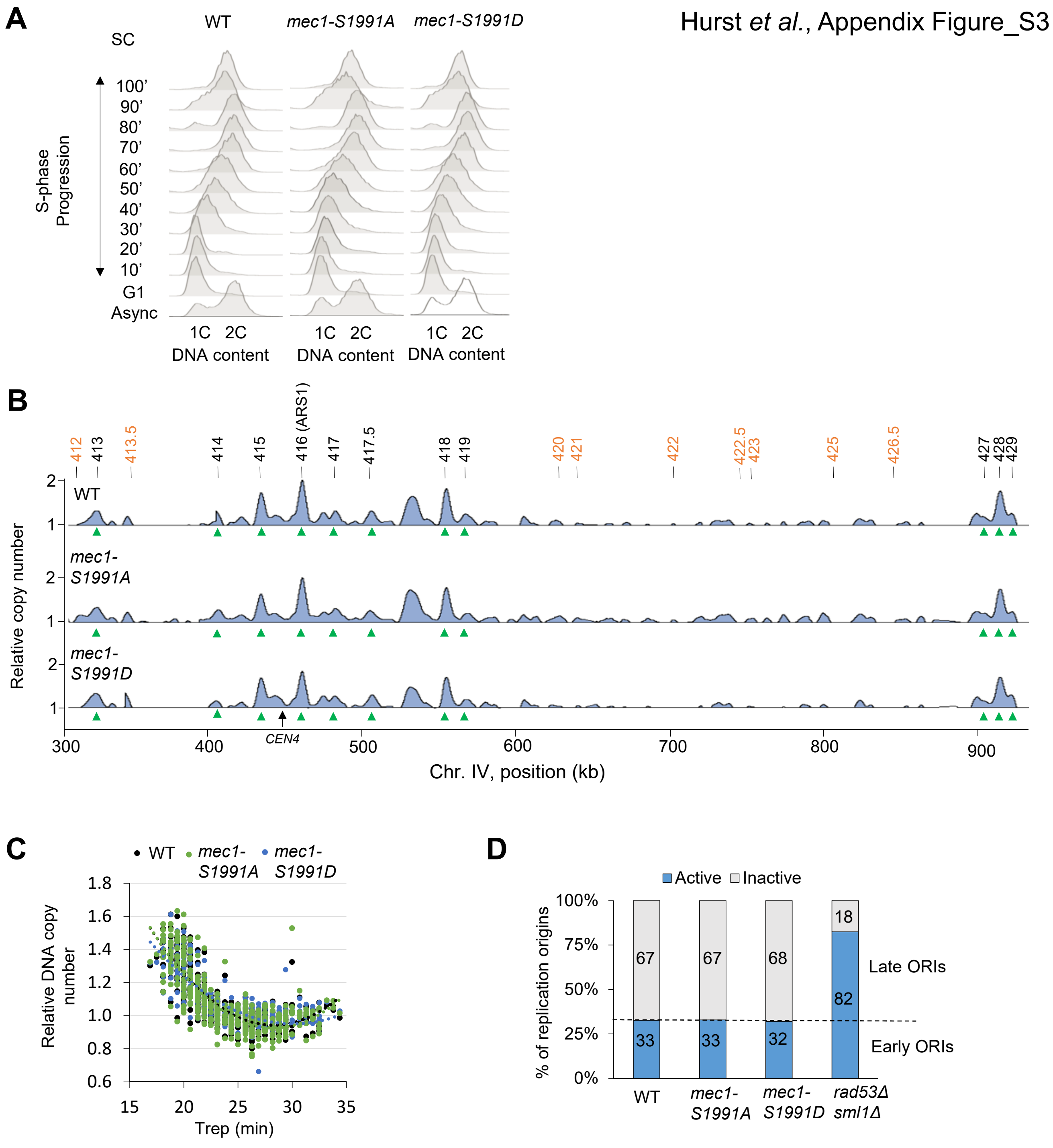

Appendix Figure S3: **G1-S transition and origin usage in *mec1-S1991* phosphomutants are intact.** (A) Flow-cytometry analysis of DNA content. Asynchronous cells were synchronized in G_1 with α-factor and released into S phase. The kinetics of G_1/S transition as well as progression through S phase are shown over time (0-100 min). (B) Illustration of the replication profile on chromosome IV in wild-type compared to *mec1-S1991A* and *mec1-S1991D* cells after α-factor synchronization and release in 0.2M HU for 60min. Positions of early (black) and late (orange) origins are indicated. Fired origins are indicated (green arrowhead). (C,D) Genome-wide analysis of origin usage on HU. In C, we show a scatter plot of relative DNA copy number in the indicated genotype versus the T_{rep} (Yabuki et al., 2002) for 386 origins. In D, the quantitation of active and inactive origins, using *rad53Δsml1Δ* strain as a positive control for aberrant late origin firing (data from Poli et al., 2012).

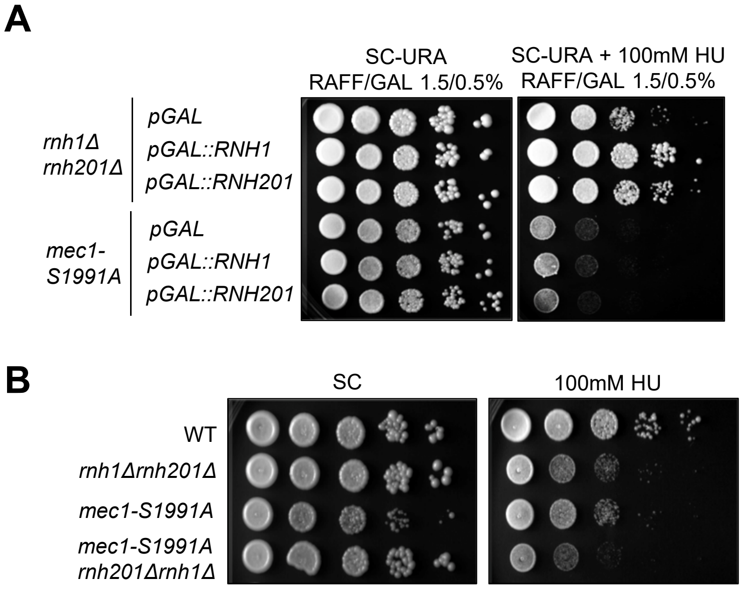

Appendix Figure S4: **RNase H overexpression does not rescue *mec1-S1991A* sensitivity to HU. (A,B)** Impact of galactose-induced RNase H overexpression on HU sensitivity. A 10-fold dilution series of cells from exponential SC-URA cultures of the indicated strains were spotted on SC-URA raffinose-galactose plates +/- 100mM HU (A) or SC glucose 2% +/- 100mM HU (B).

Appendix Methods:

Yeast strains construction:

Gene deletion and tagging were performed as described previously (Longtine et al., 1998). *BAR1* deletion and *mec1-S1991A* point mutation, were generated by CRISPR editing (Anand et al., 2017). For Epitope tagging (Pol2 tagged with HA and Mcm7 tagged with Myc), strains were generated by homologous recombination with the tagging plasmids (pYM17+ and pYM18+ (see Table EV1)).

Protein extracts, chromatin fractionation and Western blotting

TCA precipitated protein extracts were resolved by SDS-PAGE (Criterion TGX 4-15 %, Bio-Rad or with a self-made low cross linking gel for detection of the Maf1 shift, as described in Nguyen et al., 2010) and transferred to a PVDF membrane with a Bio-Rad Trans-Blot Turbo Transfer system (30 min standard mode). After blocking, proteins were either probed with 1/7500 anti-RNAPII CTD (Abcam 8WG16, ab817), 1/2000 anti-Rpb1-S5P (Clone 3E8, Merck, 04-1572), 1/7500 anti-Rpb1-S2P (Abcam, ab5095), anti-PK for Maf1-3PK strains (Novus Biologicals, NB600-381), 1/200 anti-Rad53 (clone 11G3G6, custom made by GenScript), 1/500 anti-Mcm2 (N-19, Santa Cruz, sc-9839), 1/5000 anti-tubulin (Thermo Fisher Scientific, MA1-80017) or 1/2000 anti-actin (clone C4, Sigma-Aldrich, MAB1501). Blots were scanned with an ImageQuant LAS4000 mini (GE healthcare) and semi-quantitative determination of protein level was performed using the Image J (Fiji) software using Tubulin, Actin or Mcm2 as normalizers. We note that Rpb1 degradation during unchallenged S phase was only visible when cells were grown in SC medium.

Drop assays

Exponentially growing cells were counted using a CASY (OLS system) and were diluted or concentrated to 1.10^7 cells/ml. 10-fold serial dilutions was spotted on YPAD or SC plate +/- the indicated drug. Where indicated 5-fold dilution series were used. Plates were incubated from 2 to 7 days at 25°C or 30°C. The *mec1-S1991A* deficiency on HU is often more visible on SC medium grown at 30°C for unknown reasons.

Genome-wide replication timing analysis

For genomic DNA extraction, 100 mL of yeast cells at 1 $\times 10^7$ cells per milliliter was shaken five times for 2 min in NIB buffer (17% [v/v] glycerol, 50 mM MOPS buffer, 150 mM potassium acetate, 2 mM $MgCl_2$, 500 µM spermidine, 150 µM spermine at pH 7.2) with zirconium beads on a Vibrax (VXR basic, Ika) at 4°C. DNA was isolated using Qiagen genomic DNA extraction kit according to the manufacturer's instructions. DNA was fragmented using sonication (~200- to 500-base-pair [bp] size range). Sequencing libraries were prepared using a Thru-PLEX DNA-seq kit (Rubicon Genomics). Next-generation sequencing was performed on a HiSeq 4000 (Illumina). Single-end reads of 50 bp were aligned to the *Saccharomyces cerevisiae* genome (2011) and rDNA sequence with Bowtie, allowing only perfect matches. Relative copy number was determined as the ratio of normalized reads on HU and G_1 cells. Replication profiles were smoothed with a sliding window of 1000 bp and displayed using Integrated Genome Browser version 8.2 (Nicol et al., 2009).

Pol2 (DNA Polymerase ε) chromatin immunoprecipitation and sequencing

Log phase cultures were arrested in G_1 phase at 25°C using α factor (2 µg/mL, GenePep) and then released and released into S phase by the addition of 75 µg/mL Pronase into medium containing 0.2M HU. Cells were collected 15, 60, 90 and 150 minutes after release and immediately cross-linked with final 1% formaldehyde for ChIP. In parallel, additional cells were collected for FACS staining. After 20 min, cells were quenched with glycine (125 mM final concentration) for 5 minutes and then washed twice with ice-cold, double-distilled water, pellets flash-frozen in liquid nitrogen and stored in -80°C. ChIP was performed as described by (Gutin et al., 2018), except that the on-bead library preparationwas substituted with on-bead tagmentation as described by (Schmidl et al., 2015). In detail, cell pellets were thawed on ice, washed with 1M ice-cold Sorbitol and re-suspended in Buffer Z supplemented with 10 mM β-mercaptoethanol (5 µL buffer per 1 OD600 of cells). Cells were then treated with Zymolase 100T (0.5 units per 1 OD600 of cells) for 30 minutes at 30°C and spheroplasts were then pelleted for 5 minutes at 6500 g 4°C and re-suspended in lysis buffer with NP-40 (NP) buffer (supplemented with Protease inhibitors (PI), 500 µM spermidine and 1mM β-mercaptoethanol). After cell lysis, nuclei were pelleted at 16,000g for 10 minutes at 4°C and re-suspended in NP buffer again. Sonication followed for 25 minutes (30 sec on, 30 sec off) at high intensity in a Bioruptor plus (Diagenode) cooled water bath sonicator.

Resulting lysates were vortexed for 30 seconds, kept on ice for at least 30 minutes, vortexed again and centrifuged for 10 minutes at 16,000g at 4°C. Around 3 µg of HA antibody were added to each supernatant and samples were incubated for 2.5 hours at 4°C with gentle tumbling. 20 µl protein G beads suspended in Radioimmunoprecipitation assay (RIPA) buffer (supplemented with PI) were then added to each sample, and an additional 1-hour incubation in 4°C tumbling followed. Samples were then magnetized and washed 6 times with RIPA buffer, 3 times with RIPA 500 buffer, 3 times with Lithium Chloride (LiCl) wash buffer, 3 times with 10 mM Tris pH 7.5 and once with 10 mM Tris pH8 off the magnet (all wash buffers were supplemented with PI). Tagmentation reaction was carried out using commercial Tn5 (Illumina) with 7.5 uL 2x TD buffer and 0.25 µL Tn5 enzyme per sample (final reaction volume was 15 µL). Beads were re-suspended in the tagmentation mix and the reaction was incubated for 10 minutes at 37°C. The reaction was stopped on ice with RIPA buffer, and beads were washed 3 times with RIPA buffer. Samples were resuspended in chromatin elution buffer, treated with 0.5 µg RNase A for 30 min at 37°C, 50ug Proteinase K for 2 hours at 37°C, and then de-cross-linked for 12-16 hours at 65°C. DNA was isolated with 2.2X SPRI beads purification and then amplified with KAPA Hifi hotstart ready mix PCR (after pre-activation at 98 for 3 minutes, 14 cycles) with barcoded Tn5 primers resulting in multiplexed libraries. Libraries were sequenced by an Illumina NextSeq 500 or NovaSeq kits with 50bp paired-end sequencing.

Pol2 (DNAPol ε) ChIP-seq data processing

ChIP-Seq DNA libraries were de-multiplexed using bcl2fastq (Illumina) and the paired-end data was subsequently aligned to the *S. cerevisiae* genome R64-1-1 using Bowtie 2 with the options "--end-to-end --trim-to 40 --very-sensitive". Duplicates were subsequently determined with customized Picard and genome-wide coverage for concordantly aligned, unique read-pairs calculated with genomeCoverage from BEDTools (Quinlan and Hall, 2010) using the parameters "-d -pc". All further processing was done using MATLAB. First, the total coverage was normalized so that the mean coverage in the unique regions of the genome (e.g. not telomeres, rRNA genes or transposons) was one, and subdivided into 200bp bins. For Figures 3E, the median normalized bin occupancy 30 kb around the 32 earliest ORIs (according to (Yabuki et al., 2002)) at each time points was plotted against the absolute distance, not distinguishing between up- and down-stream sequences.

DRIP-qPCR

100 mL of asynchronous log yeast cultures at 1 ×10^7 cells/ml were collected and washed twice with cold water. Cell pellets were resuspended in 2.4 ml of spheroplasting buffer (1 M sorbitol, 2mM Tris-HCl pH 8, 100 mM EDTA pH 8, 0,01% β-mercaptoethanol and 2 mg/ml Zymolyase 20T) and incubated for 30 minutes at 30°C. Pellets were washed with distilled water, resuspended with 1 ml G2 buffer (800 mM guanidine HCl, 30 mM Tris-HCl pH 8, 30 mM EDTA pH 8, 0.5% Tween-20, 0.5% Triton X-100) and incubated 1h with 10µl RNaseA (Sigma, 30mg/ml) followed by 2h at 37°C with 75 µl proteinase K (20 mg/ml) at 50°C. DNA was extracted with chloroform-isoamyl alcohol 24:1 (Sigma), precipitated with isopropanol, and recovered on a glass rod. Then, DNA was resuspended in TE 1X and digested at 37°C overnight with a restriction enzyme cocktail containing *EcoRI, XbaI, HindIII, BrsGI* and *SspI* (New England Biolabs). DNA fragments were purified on a Sephadex column (GE healthcare). Half of the DNA was treated with 25U of RNaseH (New England Biolabs) for 3h at 37°C. DRIP was performed with 5 µg of DNA and 10 µl of S9.6 antibody (1mg/ml, Antibodies inc.) incubated overnight rotating at 4°C in binding buffer (10 mM NaPO4 pH 7, 140 mM NaCl, 0.05% Triton X-100 in TE). The DNA-antibody mixture was incubated with Dynabeads M280 sheep anti-mouse (Life Technologies) for 4h at 4°C rotating at low speed in a final volume of 500 µl of binding buffer. Beads were washed five times with binding buffer and DNA was eluted in 120 µl elution buffer (50 mM Tris pH 8, 10 mM EDTA, 1% SDS) at 65°C for 10 minutes. Eluates were incubated 1h with 10 µl proteinase K at 50°C and purified with the AccuPrep clean-up purification kit (Bioneer).

Fixed Microscopy and image analysis

Cells were fixed in fresh paraformaldehyde (PFA) 4% w/v for 1 min, washed 3 times in PBS and then attached to a #1.5H (0.17 mm) glass coverslip using Concanavalin A. They were imaged by taking 50 z slices of 200 nm thickness. A Zeiss Axioimager Z2 was used to acquire images equipped with a Hamamatsu ORCA Flash 4 LT+ camera, Marzhauser XY motorized stage, Prior Nano scan Z piezo, and a Plan apochromat 100x NA=1.46 oil objective was used. GFP was excited using a LED XCite 120 LED. Fluorescence images were deconvolved using Huygens professional and the classic maximum likelihood estimate algorithm with a signal/noise ratio of 1, automatic background estimation and 40 iterations. Calculation of foci into nuclear zones was done as in (Horigome et al., 2014) for at least 300 cells/condition.

Live Microscopy and image analysis

Live yeasts were observed using a Zeiss Axio-Observer widefield microscope equipped with a Hamamatsu ORCA Flash4 camera and a plan apochromat 63X NA= 1.4 oil objective and a XCite 120 LED fluorescence light source. Rpb1-GFP was image using a Carl Zeiss FS38 filterset (Ex. BP 470/40 Dichroic FT495 & Em. BP 525/50). Imaging was performed in sterile-filtered SC medium using an Onix CellAsic microfluidic chamber for haploid yeast (Merck, Y04C-02-5PK) to regulate cell synchronization in α-factor and the release into S phase. Focus drift was avoided in between timepoints using a Carl Zeiss hardware autofocus loop (Definite Focus2). Time-lapse series (80 min in total) of 15 optical slices per stack of 0.4 µm were acquired every 10 minutes. The sCMOS binning mode was set to 2x2. In these conditions, bleaching did not occur until 3h. Although the dataset was undersampled, images were deconvolved using the Huygens professional deconvolution suite, theoretical PSF and the classic maximum likelihood estimation algorithm. The main deconvolution parameters were 40 iterations (and a quality factor change threshold of 0.01). Before deconvolution, noise was corrected using a signal/noise ratio of 40 and background automatically remove (using a background radius of 0.7). To avoid any interference of this correction, bleaching correction was not used. Nuclei were detected and segmented using Imaris and a fixed threshold value. Each nucleus was tracked through the time series. The integrated nuclear intensity was then calculated for each cell nucleus."

Proteomic data analysis

Proteomic data analysis was done with Proteome Discoverer PD2.4 (ThermoFischer Scientic) according to (Challa *et al*, 2021) with minor modification. Briefly, MS raw data were loaded as fractions, re-calibrated and searched against the Uniprot Yeast database (downloaded from Uniport on April 10 2019 with the query: organism: "*Saccharomyces cerevisiae* (strain ATCC 204508 / S288c) (Baker's yeast) [559292]" AND proteome:up000002311) and commonly observed contaminants. The search parameters were set to fully tryptic digestion, minimum peptide length of six amino acids, maximum of two missed cleavages, static modifications of TMTpro 16plex (+304 Da) at lysine and peptide N-termini, PreOmics NHS-iST Cys alkylation (+113 Da). Protein N-termini were allowed to be dynamically modified with TMTpro 16plex or acetyl (42 Da), without, or with initiator methionines removed (+173 Da for Met-loss+TMT16plex or -89 Da for Met-loss+Acetyl, respectively). The peptide and protein

identification false discovery rate was set to 0.01 based on the target-decoy (concatenated) search strategy (Elias and Gygi, 2010). Protein abundances were calculated based on the summed signal-to-noise values of all unique and razor peptide reporter ion signals above 10. Peptide abundances with more than 50% isolation interference (Co-isolation threshold) were not considered. Protein abundances were normalized based on the total peptide amount, resulting in equal sums of abundances for all samples. Filtered tables were exported for further analysis in RStudio (version 1.2.5033) and R (version 4.0.0). In brief, a detection in all four replicates (2 biological replicates divided each in two technical replicates) of at least one condition with at least two peptides was required to quantify the abundance of those proteins with an Entrez Gene ID. To determine differential abundances, abundance values were log2 transformed after addition of pseudocounts, and then subjected to differential analysis using linear models as implemented in the R package limma (3.44.3). Significance of differential abundance was defined by conjunct minimal threshold criteria for FDR-adjusted p values (empirical Bayes method) and for fold-changes (adj.P-Val < 0.1 and logFC > 0.5).
