# Supplementary material for: A regulatory phosphorylation site on Mec1 controls chromatin occupancy of RNA polymerases during replication stress
Source: EMBO J. 2021 Sep 27;40(21):e108439. doi: 10.15252/embj.2021108439 (PMC8561635; doi:10.15252/embj.2021108439)
Supplement: Supplementary file 2 — Expanded View Figures PDF [file EMBJ-40-e108439-s011.pdf]

Expanded View Figures

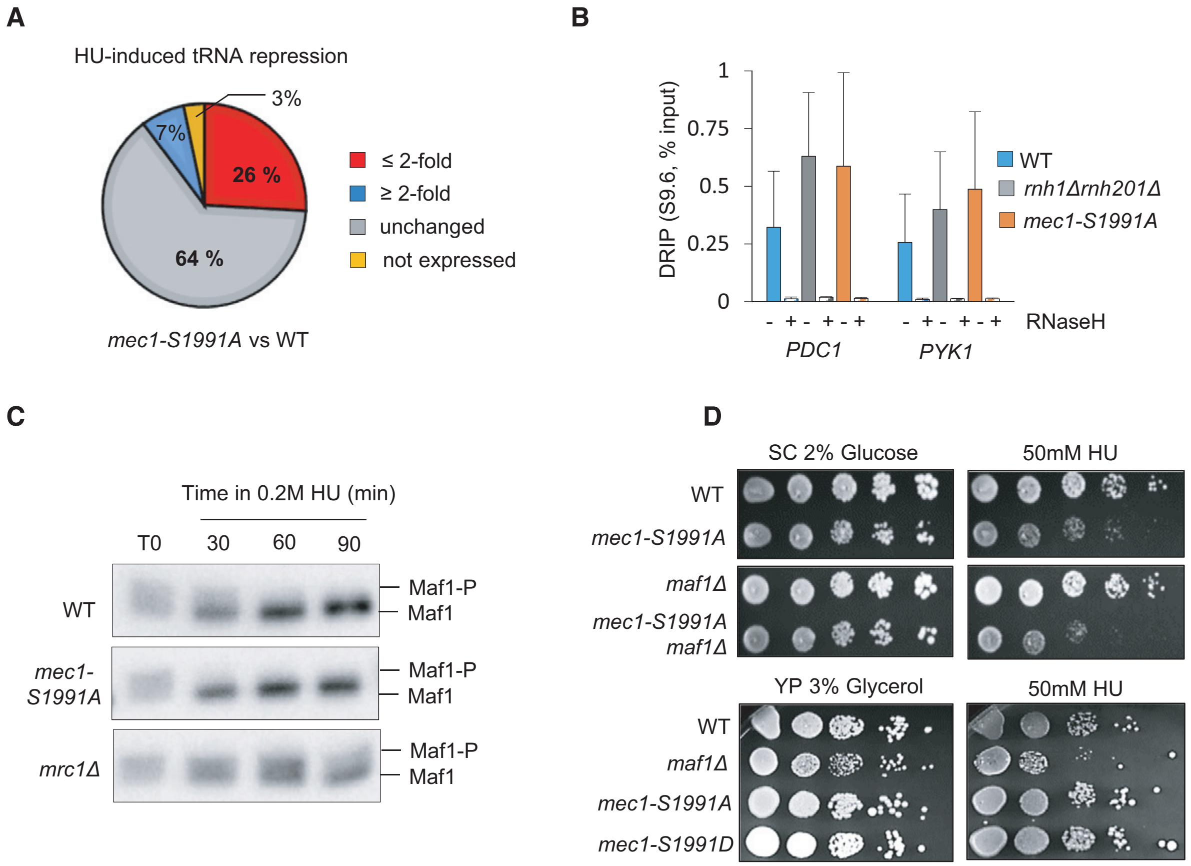

Figure EV1. Mec1-S1991 phosphorylation limits RNAPIII transcription during HU-induced replication stress.

A RNA-seq signals obtained from asynchronous culture and after 90 min of HU treatment allowed to determine the fold change of HU-induced tRNA repression (HU/as) in wild-type and *mec1-S1991A* cells. Pie charts illustrate the proportion of tRNA genes that are either not expressed (yellow), unchanged in steady-state level +/− HU treatment (unchanged, gray) and differentially repressed upon HU treatment in the *mec1-S1991A* mutant compared with wild-type cells (decreased repression in the mutant over wild-type ≤ 2-fold in red, increased ≥ 2-fold in blue).

B DNA–RNA hybrid level measured by DRIP-qPCR at *PDC1* and *PYK1* in asynchronous cells of the indicated genotype. Where indicated, samples were treated with RNase H as a technical control. SEM (n = 3 biological replicates) is indicated.

C Asynchronous cells of the indicated genotype (+Maf1-3PK) were treated with 0.2 M HU for 30, 60, and 90 min. Total protein extracts were subjected to SDS–PAGE with a low cross-linked gel for sufficient separation. Differentially phosphorylated forms of Maf1 were detected with a PK antibody.

D Drop assay showing a 10-fold dilution series of cells from exponential SC cultures of the indicated strains that were spotted on SC supplemented either with 2% glucose or 3% glycerol +/− 50 mM HU.

Source data are available online for this figure.

© 2021 The Authors

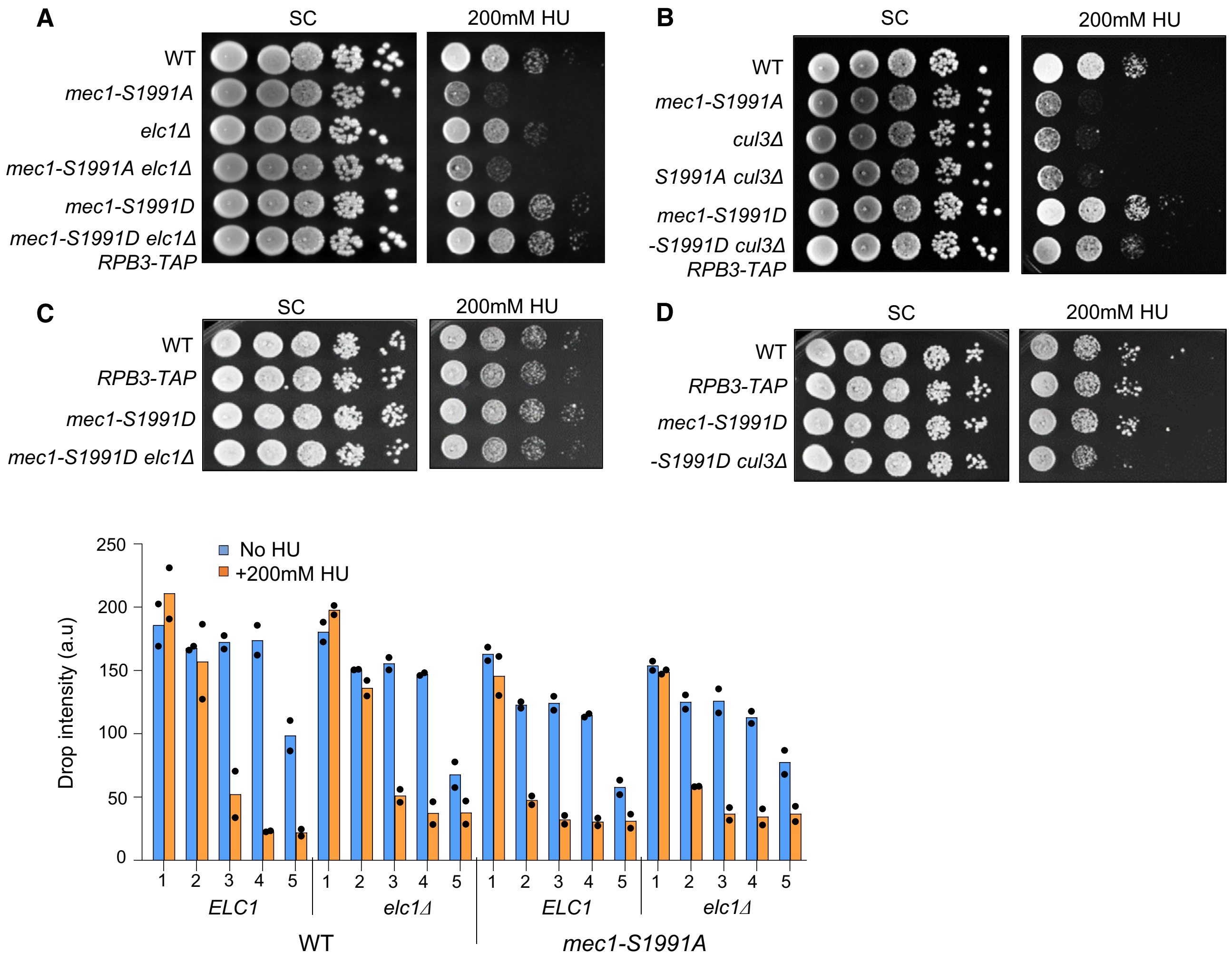

Figure EV2. Comparison of *mec1-S1991A* and *elc1Δ-cul3Δ* growth defects on HU.

A–D Drop assays showing a 10-fold dilution series of cells from exponential SC cultures of the indicated strains that were spotted on SC +/– 200 mM HU. Histogram presents quantification of two independent HU sensitivity assays with mean and individual data point values indicated for each yeast dilution.

Figure EV3. Rescue of *mec1-S1991A* growth defect on HU by RBP3-TAP requires the PAF1 complex subunit Rtf1.

A Analysis of inter-origin distances at the single-molecule level by DNA combing in the indicated strains. Graph depicting the distribution of inter-origin distances determined by DNA combing after 90 min in S phase + 0.2 M HU. Box, 25–75 percentile range. Whiskers, 10–90 percentiles range. Median is indicated in kb. ns (*P*-value > 0.01), by Mann–Whitney rank-sum test. WT (n = 173), *RPB3-TAP* (n = 89), and *mec1-S1991A RPB3-TAP* (n = 118). The WT data set is from Fig 3 and is included for comparison.

B–F Drop assay showing a 10-fold dilution series of cells from exponential SC cultures of the indicated strains that were spotted on SC +/– the indicated drug. *RTF1* encodes an essential subunit of the PAF1 complex. (E) In addition to rescuing *mec1-S1991A* phenotype, expression of Rpb3-TAP slightly improves WT resistance to high doses of HU on YPD.

Source data are available online for this figure.

 © 2021 The Authors

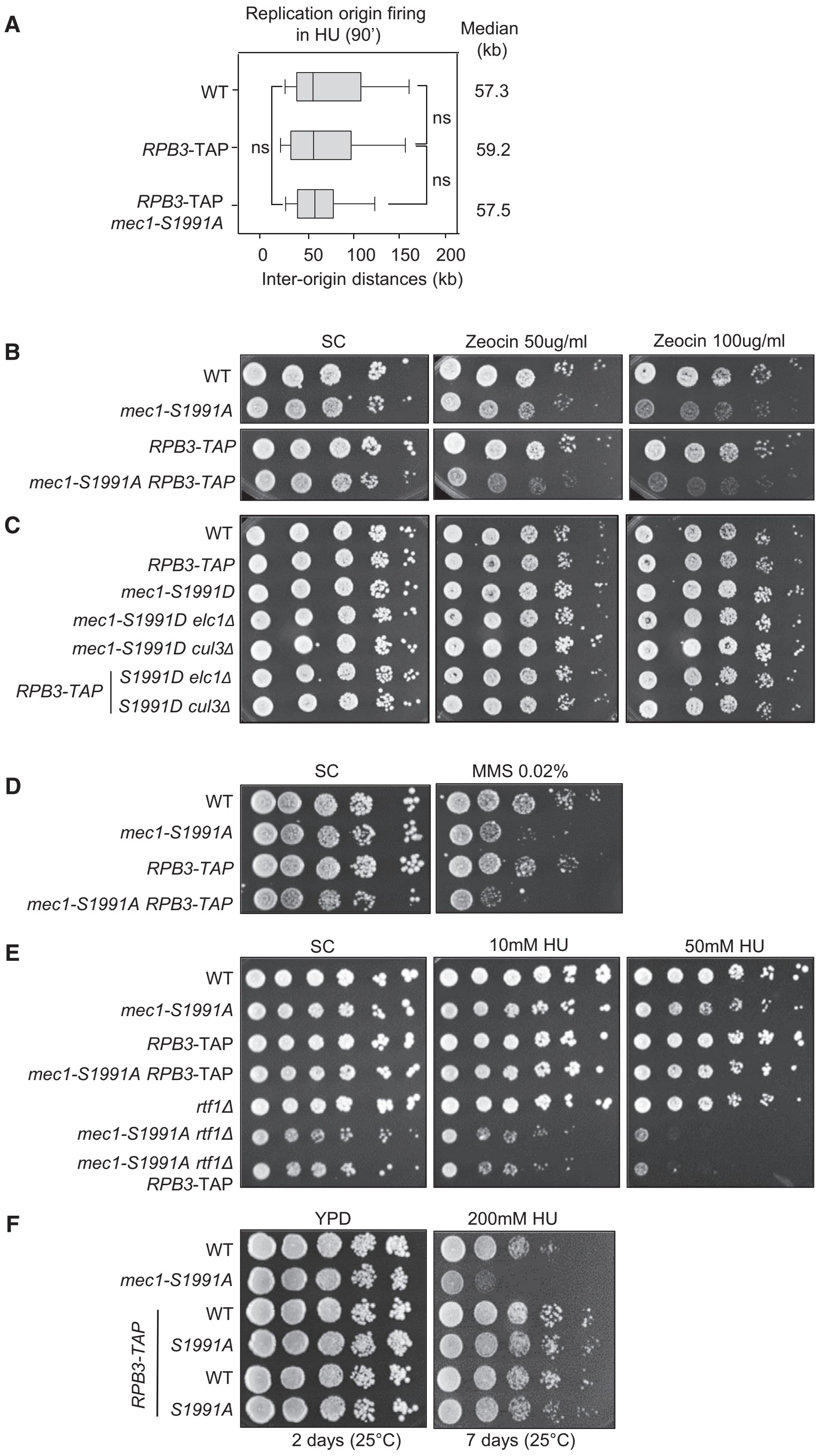
A
Replication origin firing in HU (90')
Median (kb)
WT
RPB3-TAP
RPB3-TAP mec1-S1991A
ns
ns
ns
57.3
59.2
57.5
0 50 100 150 200
Inter-origin distances (kb)
B
SC
Zeocin 50ug/ml
Zeocin 100ug/ml
WT
mec1-S1991A
RPB3-TAP
mec1-S1991A RPB3-TAP
C
WT
RPB3-TAP
mec1-S1991D
mec1-S1991D elc1Δ
mec1-S1991D cul3Δ
RPB3-TAP
S1991D elc1Δ
S1991D cul3Δ
D
SC
MMS 0.02%
WT
mec1-S1991A
RPB3-TAP
mec1-S1991A RPB3-TAP
E
SC
10mM HU
50mM HU
WT
mec1-S1991A
RPB3-TAP
mec1-S1991A RPB3-TAP
rtf1Δ
mec1-S1991A rtf1Δ
mec1-S1991A rtf1Δ RPB3-TAP
F
YPD
200mM HU
WT
mec1-S1991A
RPB3-TAP
WT
S1991A
WT
S1991A
2 days (25°C)
7 days (25°C)

Figure EV3.

Figure EV4. The *mec1-S1991A* allele alters the kinome response to HU-induced replication stress.

A Phosphopeptide abundances (log_2 ratio [WT/*mec1-S1991A*]) in asynchronous cells. Colored dots are factors with significantly different phosphopeptide scores (WT/*mec1-S1991A*) > 4 (blue) or < 4 (red) and *P*-value < 0.05 (Student's paired *t*-test, biological replicates *n* = 3; significant phosphopeptides *n* = 97 and *n* = 26, respectively). Full list in Dataset EV4.

B Venn diagram showing the overlap of *mec1-S1991A*-dependent phosphotargets between asynchronous (*n* = 75) and HU-treated (*n* = 90) cells.

C Mec1-S1991-dependent phosphotargets in asynchronous, HU-treated cells or common between both conditions.

D, E Drop assay showing a 10-fold dilution series of cells from exponential SC cultures of the indicated strains that were spotted on SC +/− 50 mM HU.

F *mec1-S1991A*-dependent phosphotargets (*n* = 90) Gene Ontology on HU-treated cells.

G LacO repeats were inserted upstream of *GAL1* in order to visualize the locus in the presence of LacI-GFP. Three zones of equal volume were defined based on erosion from the nuclear pore-tagged ring. Zone 1 corresponds to the nuclear periphery and zone 3 to the center of the nucleus.

H Drop assay showing a 10-fold dilution series of cells from exponential SC cultures of the indicated strains that were spotted on SC +/− 0.2 M HU. *MLP1* and *MLP2* encode subunits of the inner nuclear pore basket that are implicated in the binding of highly transcribed genes.

Source data are available online for this figure.

© 2021 The Authors

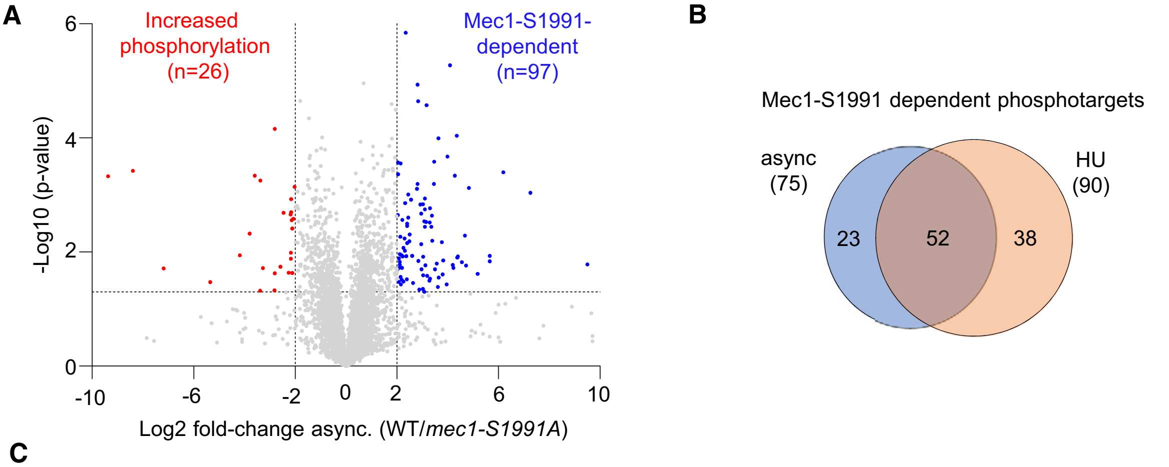

C

Condition	ORF name
Common HU & async (n=52)	BNI4 EIS1 SBH1 GIP2 TAT1 BLM10 AMS1 **ADR1** SLA2 GIS1 **ASG1** **CYC8** HSP12 GCS1 **BCY1** TPS3 **VTS1** GPH1 YKR018C HXT5 **CIN5** NOC2 FYV8 **DOT6** UBP3 MDS3 MSC3 SMY2 **ISW1** GSY2 CUE4 QRI1 UIP4 YER079W INP53 ARO4 **FUN30** **RNR1** TSL1 **TOP2** **GSY1** SUI3 MPS2 **HOS4** **MLP1** **KNS1** DCS2 CDC60 NVJ1 CAB3 IGD1 RPI1
Async only (n=23)	TDA11 SSE2 RET2 YPT7 YAK1 SDS24 YMR144W BNA7 PFK2 SSD1 GPD1 SLD3 **NUP53** PLM2 **TAF2** SEC16 **RTF1** TPM1 PCT1 **MMS4** DCS1 YLR177W BOI2
HU only (n=39)	YDR098C-B HBT1 YLR049C SHS1 PTK2 CAF120 COX20 **POB3** YPR091C ATG26 TOM71 **NUP2** POL12 PSD1 GLO3 **NOT5** **ROX1** PIG2 SPC110 SEC31 ABP1 **YOX1** SFB2 **MRE11** YNL115C **SWI5** TSR4 YNL011C **REG1** MSN4 LSP1 **SNF2** EDE1 YBL100W-B **HXK2** HSP26 **SAS4** YPK9

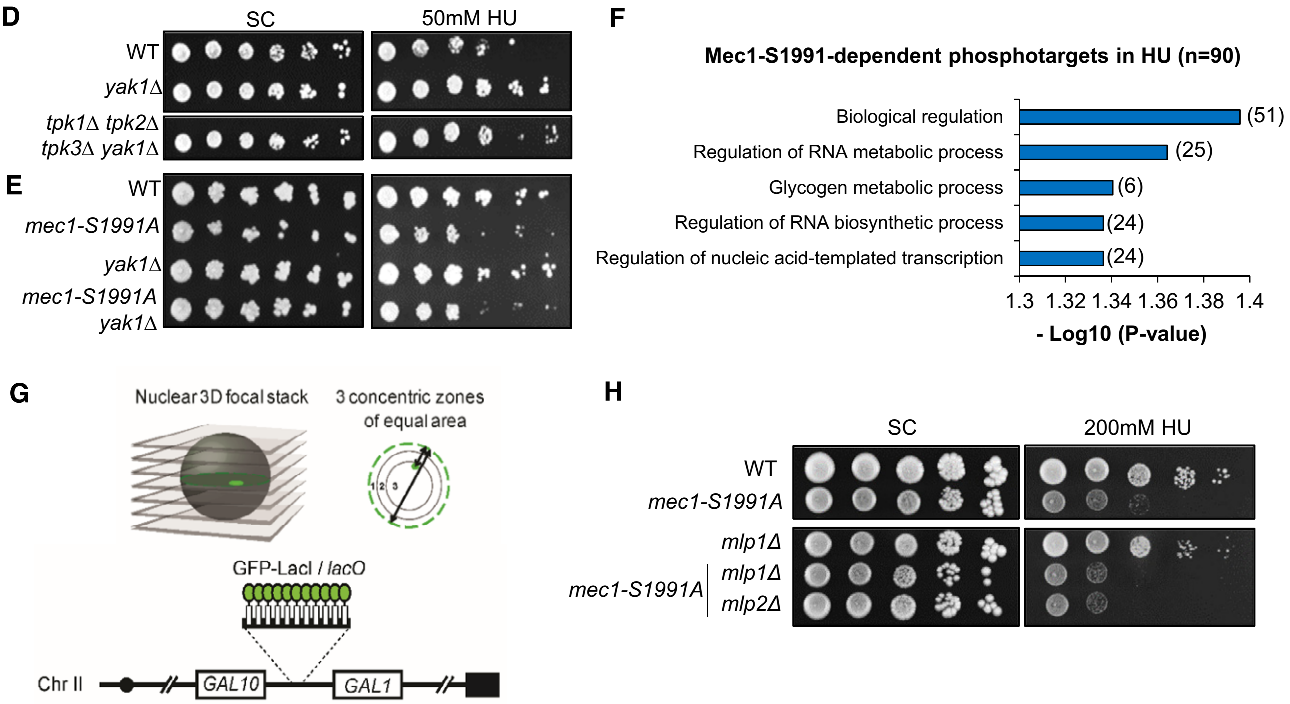

Figure EV4.

Figure EV5. Unchallenged DNA replication transiently reduces RNAPII chromatin binding in a Mec1-dependent manner.

A–C RNAPII retention on chromatin in normal S phase was assessed by ChIP-qPCR by measuring the level of Rpb1 both in G_1 and after 70 min in S phase at 16°C in wild-type and *mec1Δsml1Δ* cells. Rpb1 enrichment was quantified at loci known to generate transcription-replication conflicts: *PDC1* (A), *PYK1* (B), and *YEF3* (C). The mean percentage of Rpb1 kept on chromatin in S phase is indicated as a ratio of the S phase/G_1 level. Data are expressed as percentage of input. SEM (n = 3 biological replicates) is indicated.

D RNAPII level on chromatin was assessed by ChIP-qPCR by measuring the level of Rpb1 both in G_1 and after 70 min in S phase at 16°C in wild-type and *mec1Δsml1Δ* cells. Data are expressed as percentage of input. SEM (n = 3 biological replicates) is indicated.

E mRNA level measured by RT–qPCR in G_1 phase and after 70 min in S phase for several loci in wild-type cells. Expression levels are normalized to *ACT1*. Mean and individual data point values (n = 2 biological replicates) are indicated.

F, G Flow-cytometry analysis of DNA content. Asynchronous cells were synchronized in G_1 with α-factor and released into S phase at 25°C (F) or 16°C (G) in YPAD. The kinetics of G_1/S transition as well as progression through S phase are shown over time.

© 2021 The Authors

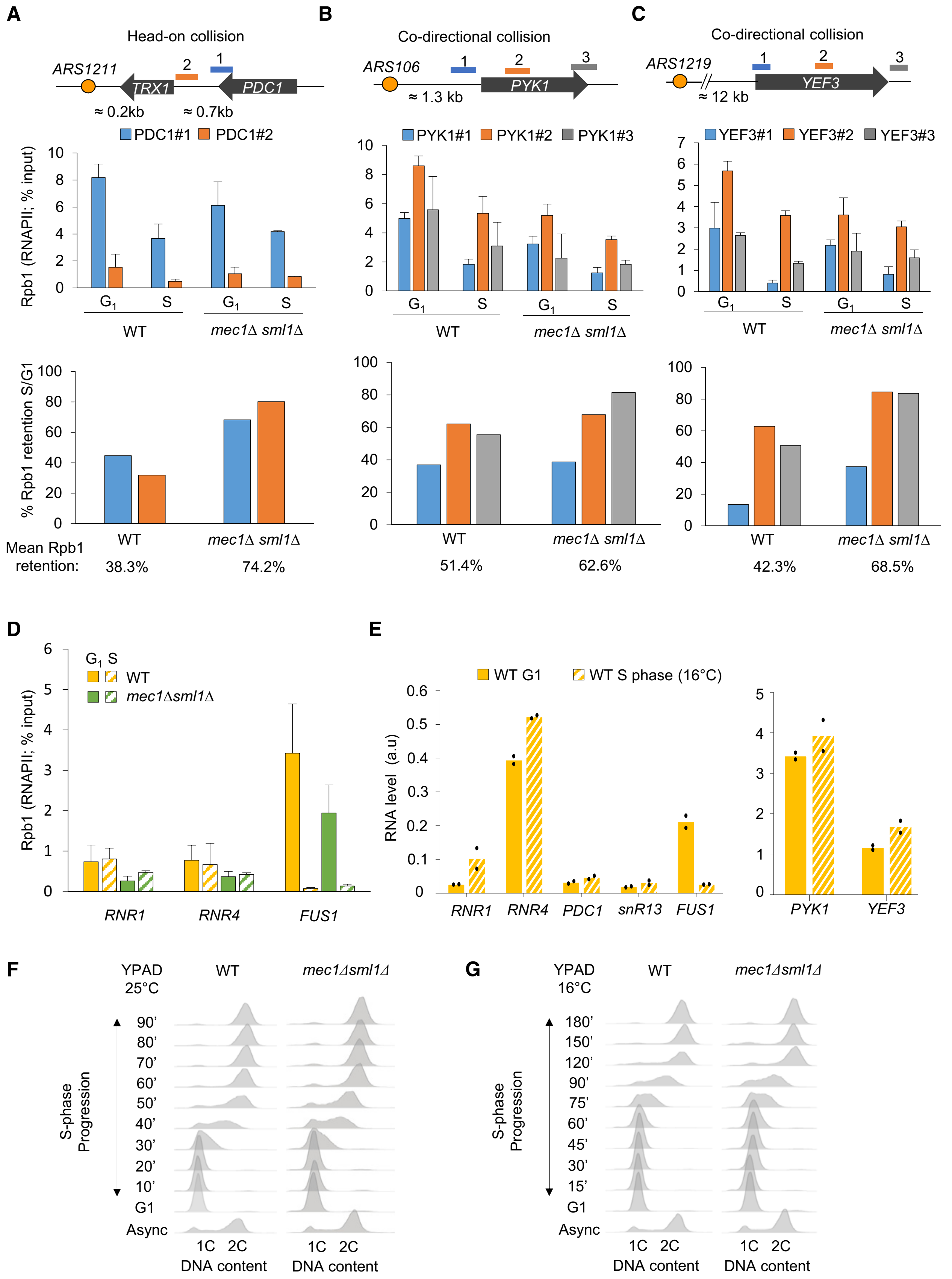
A
Head-on collision
ARS1211
TRX1
PDC1
≈ 0.2kb
≈ 0.7kb
PDC1#1 PDC1#2
Rpb1 (RNAPII; % input)
WT
mec1Δ sml1Δ
% Rpb1 retention S/G1
Mean Rpb1 retention:
38.3%
74.2%
B
Co-directional collision
ARS106
PYK1
≈ 1.3 kb
PYK1#1 PYK1#2 PYK1#3
51.4%
62.6%
C
Co-directional collision
ARS1219
YEF3
≈ 12 kb
YEF3#1 YEF3#2 YEF3#3
42.3%
68.5%
D
G1 S
WT
mec1Δsml1Δ
RNR1
RNR4
FUS1
E
WT G1
WT S phase (16°C)
RNA level (a.u)
RNR1 RNR4 PDC1 snR13 FUS1
PYK1 YEF3
F
YPAD 25°C
WT
mec1Δsml1Δ
S-phase Progression
90’ 80’ 70’ 60’ 50’ 40’ 30’ 20’ 10’ G1 Async
1C 2C
DNA content
G
YPAD 16°C
180’ 150’ 120’ 90’ 75’ 60’ 45’ 30’ 15’ G1 Async

Figure EV5.
