# Supplementary figures and images for: A regulatory phosphorylation site on Mec1 controls chromatin occupancy of RNA polymerases during replication stress
Source: EMBO J. 2021 Sep 27;40(21):e108439. doi: 10.15252/embj.2021108439 (PMC8561635; doi:10.15252/embj.2021108439)

## Drop assay Source Data for Figure\_EV1D

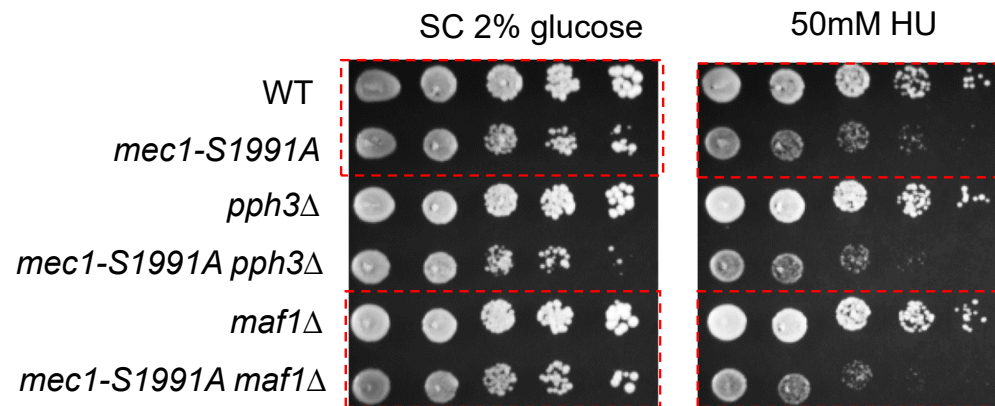

Supplement: Supplementary file 11 — Source Data for Expanded View [file EMBJ-40-e108439-s017.zip › EMBOJ-2021-108439R_Hurst_et_al_Source_Data_FigureEV1D.pdf]

## Drop assay Source Data for Figure\_EV3B

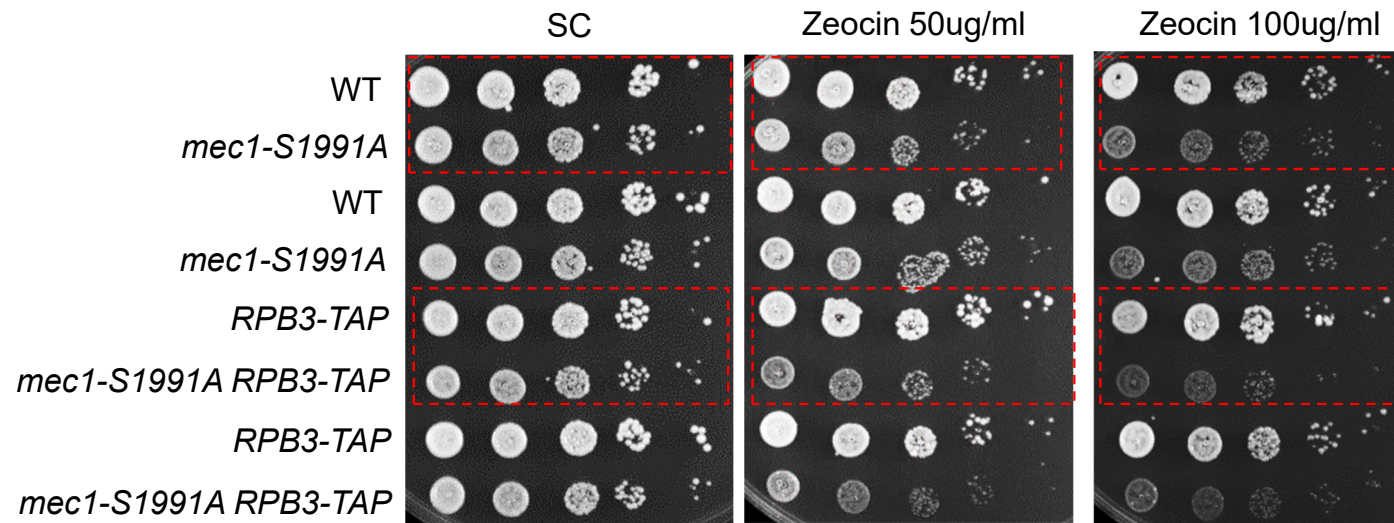

Supplement: Supplementary file 11 — Source Data for Expanded View [file EMBJ-40-e108439-s017.zip › EMBOJ-2021-108439R_Hurst_et_al_Source_Data_FigureEV3B.pdf]

## Drop assay Source Data for Figure\_EV4D

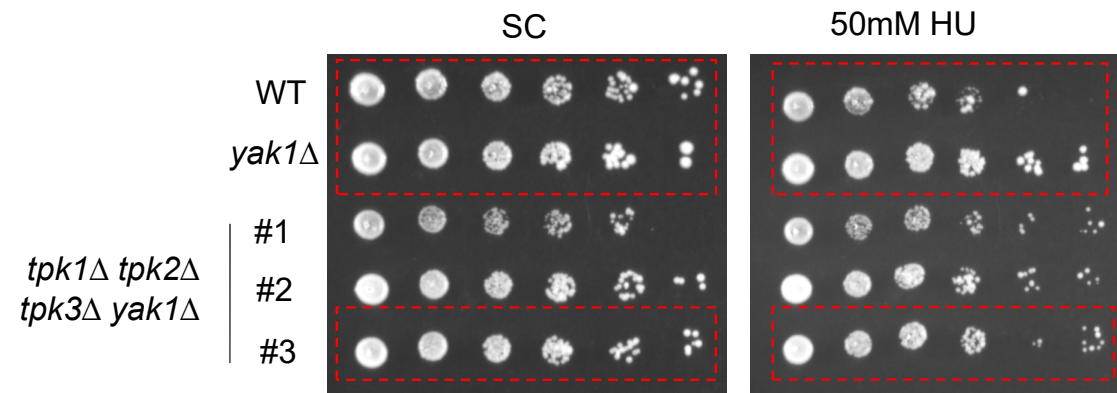

Supplement: Supplementary file 11 — Source Data for Expanded View [file EMBJ-40-e108439-s017.zip › EMBOJ-2021-108439R_Hurst_et_al_Source_Data_FigureEV4D.pdf]

## Drop assay Source Data for Figure\_EV4G

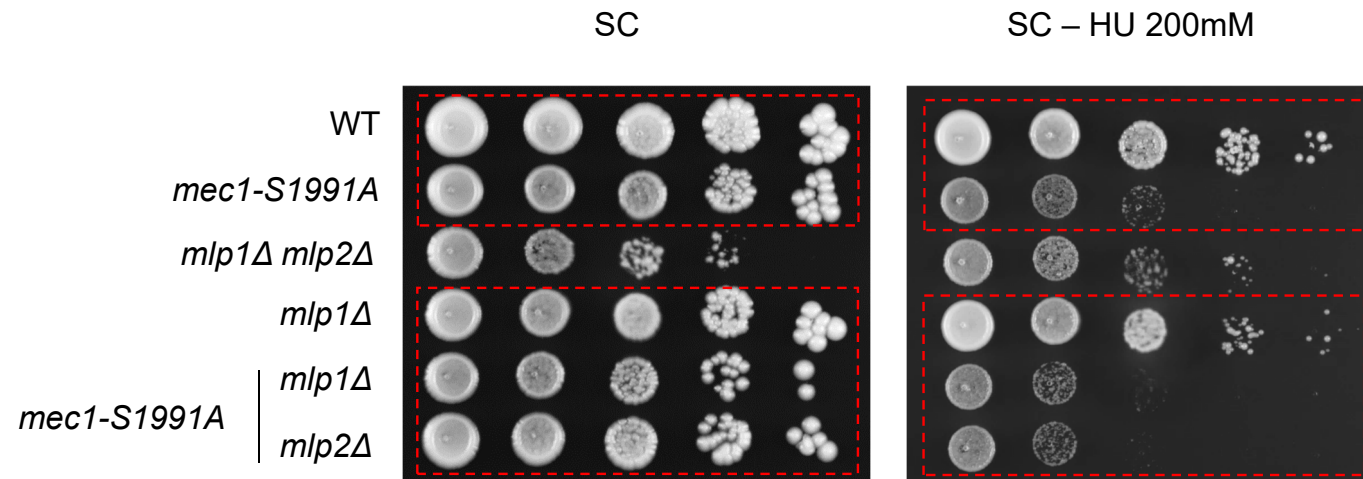

Supplement: Supplementary file 11 — Source Data for Expanded View [file EMBJ-40-e108439-s017.zip › EMBOJ-2021-108439R_Hurst_et_al_Source_Data_FigureEV4G.pdf]

Western Blot Source Data for Figure\_2C

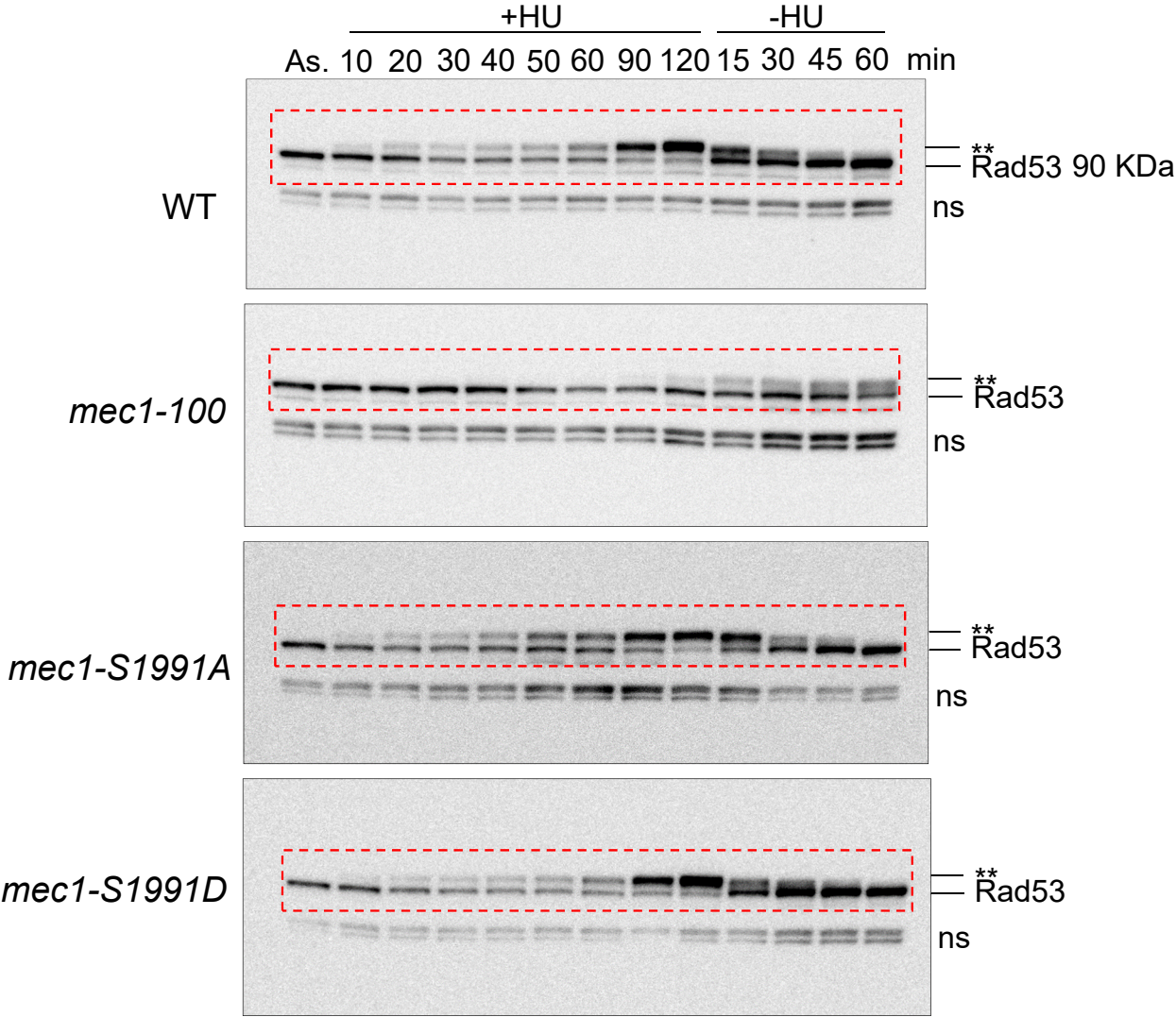

Supplement: Supplementary file 12 — Source Data for Figure 2 [file EMBJ-40-e108439-s016.zip › EMBOJ-2021-108439R_Hurst_et_al_Source_Data_Figure2C.pdf]

Drop assay Source Data for Figure\_2D

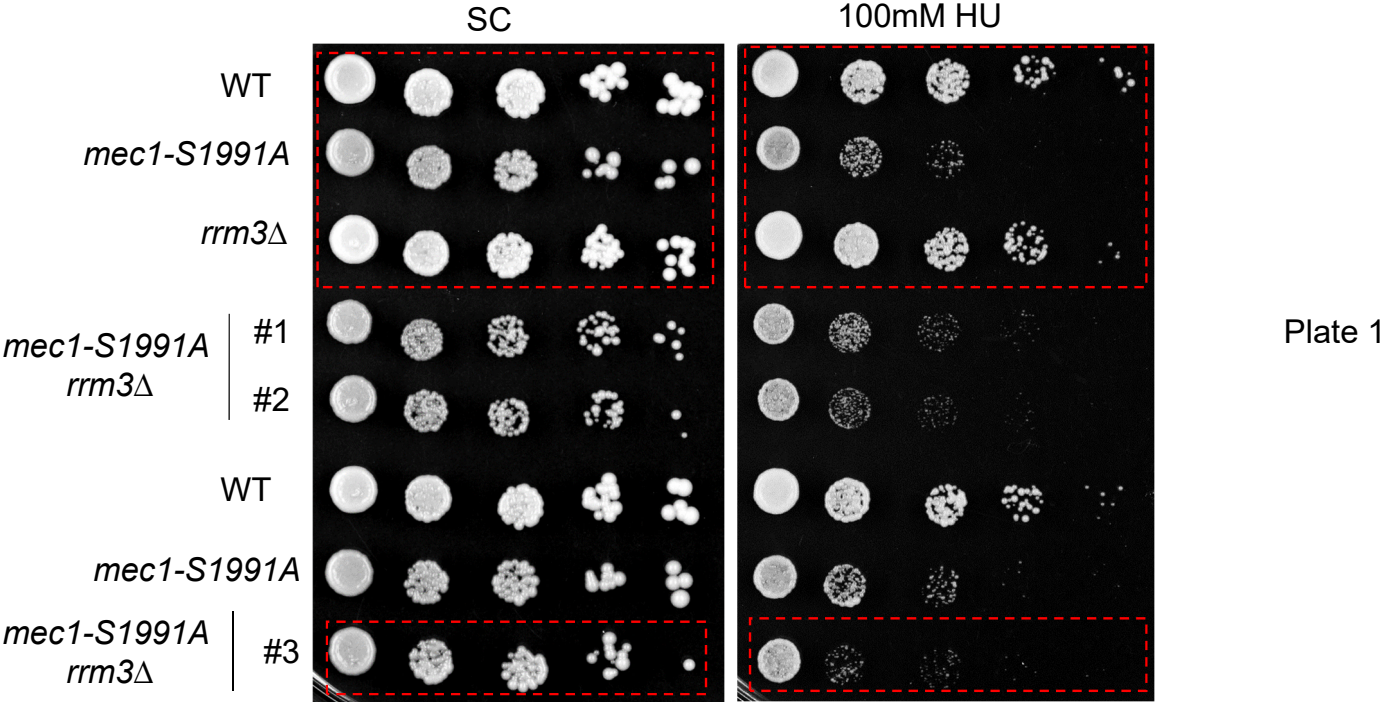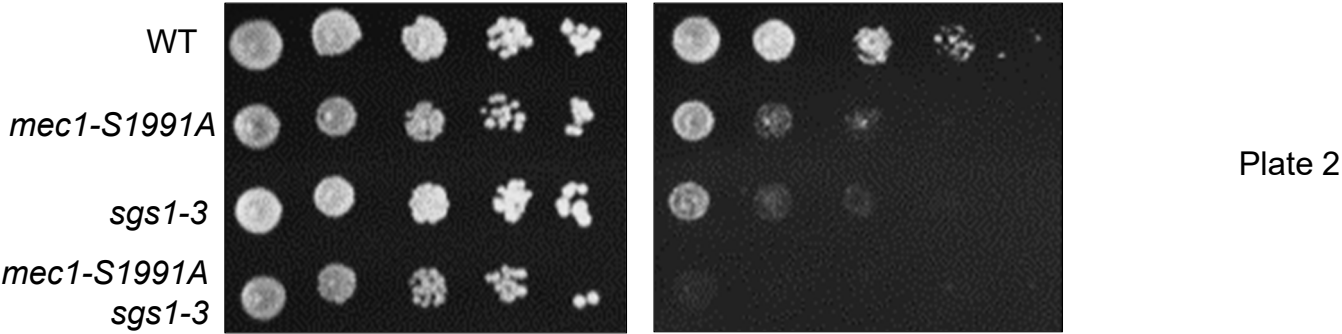

Supplement: Supplementary file 12 — Source Data for Figure 2 [file EMBJ-40-e108439-s016.zip › EMBOJ-2021-108439R_Hurst_et_al_Source_Data_Figure2D.pdf]

# Drop assay Source Data for Figure\_2F

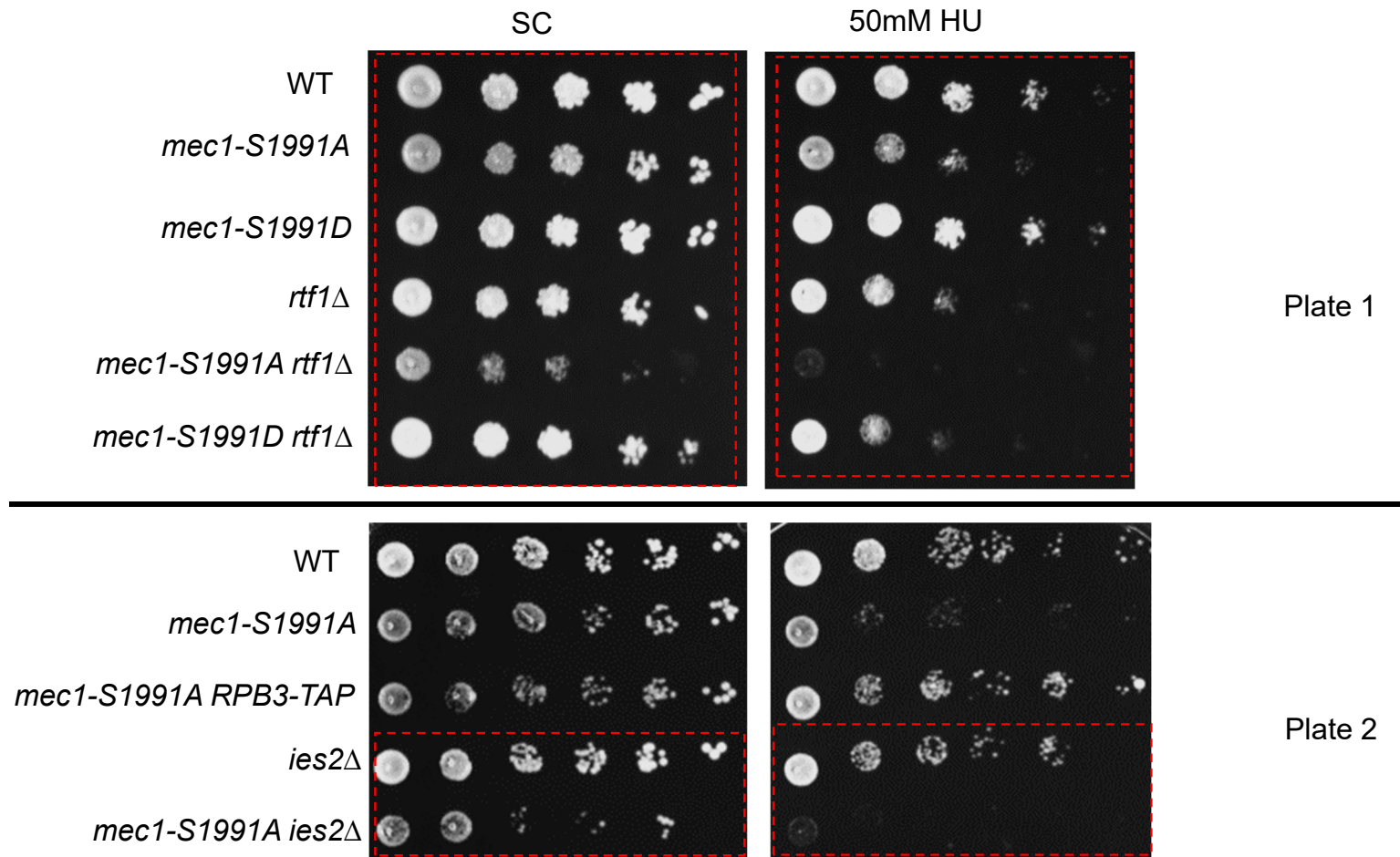

Supplement: Supplementary file 12 — Source Data for Figure 2 [file EMBJ-40-e108439-s016.zip › EMBOJ-2021-108439R_Hurst_et_al_Source_Data_Figure2F.pdf]

### Western Blot Source Data for Figure\_5A

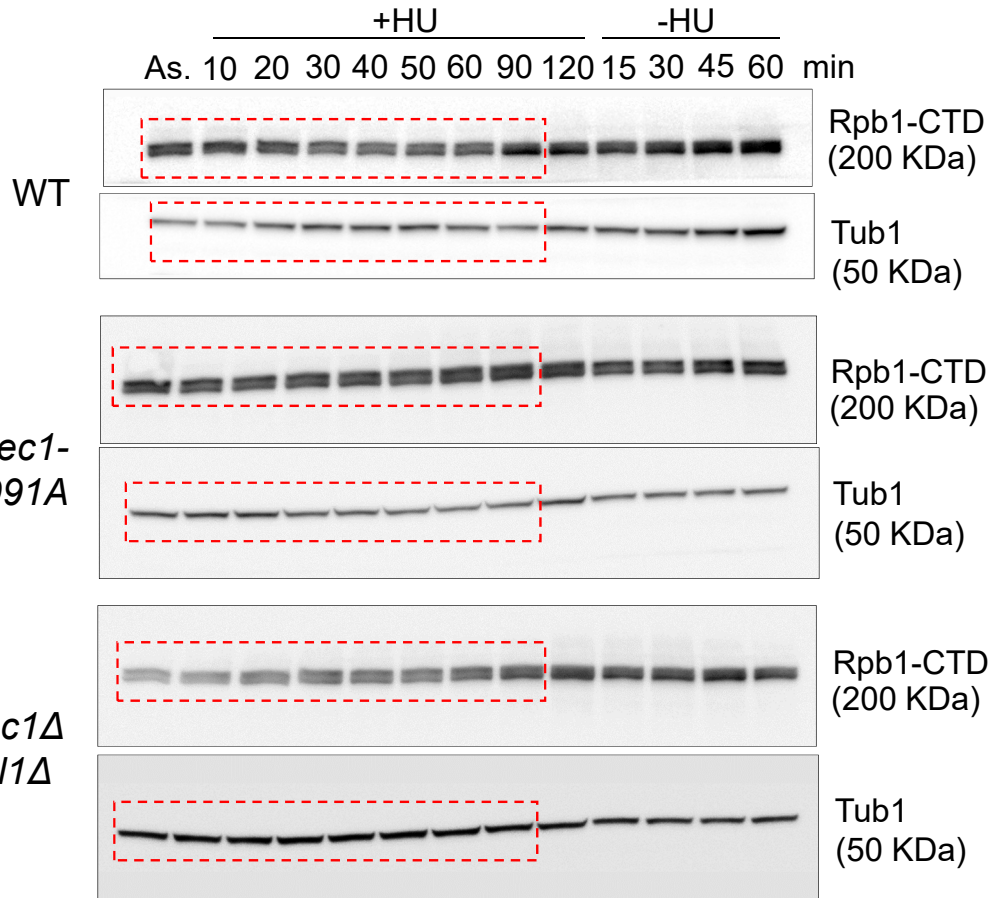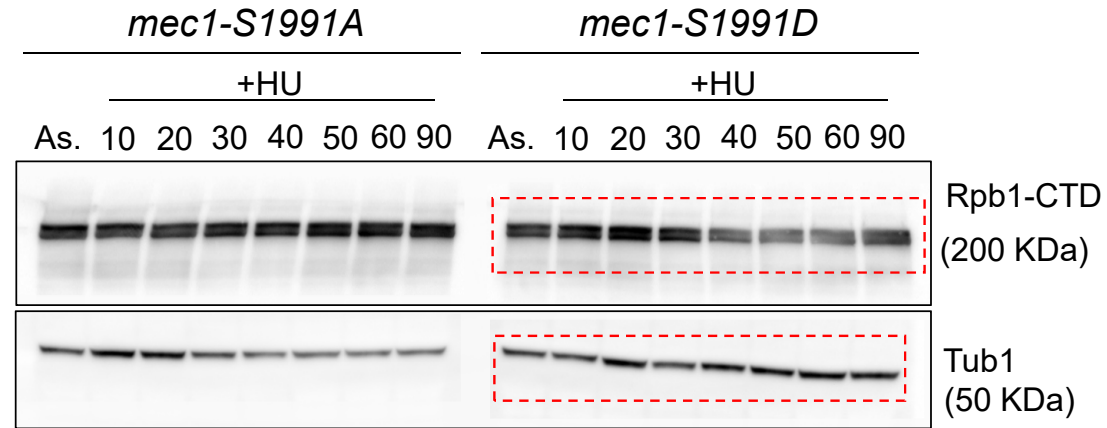

Supplement: Supplementary file 14 — Source Data for Figure 5 [file EMBJ-40-e108439-s002.zip › EMBOJ-2021-108439R_Hurst_et_al_Source_Data_Figure5A.pdf]

## Western Blot Source Data for Figure\_5C

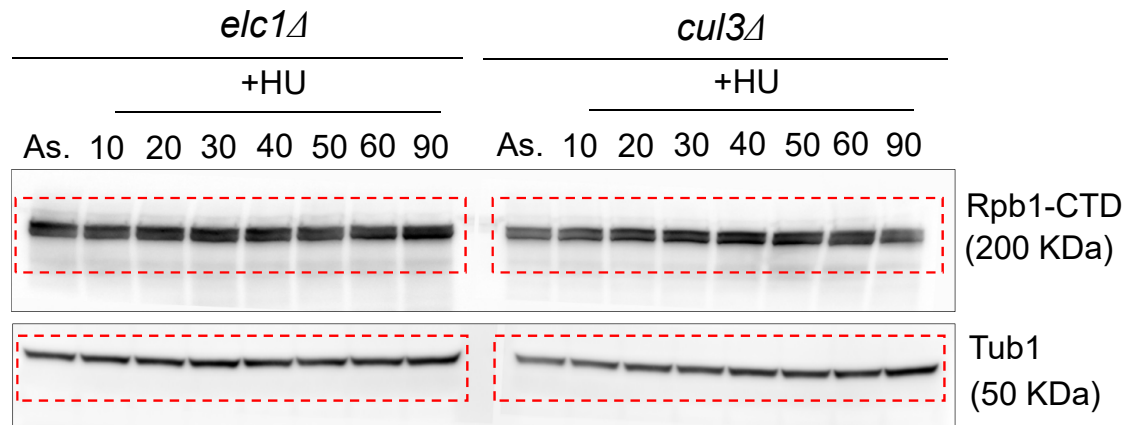

Supplement: Supplementary file 14 — Source Data for Figure 5 [file EMBJ-40-e108439-s002.zip › EMBOJ-2021-108439R_Hurst_et_al_Source_Data_Figure5C.pdf]

## Western Blot Source Data for Figure\_6A

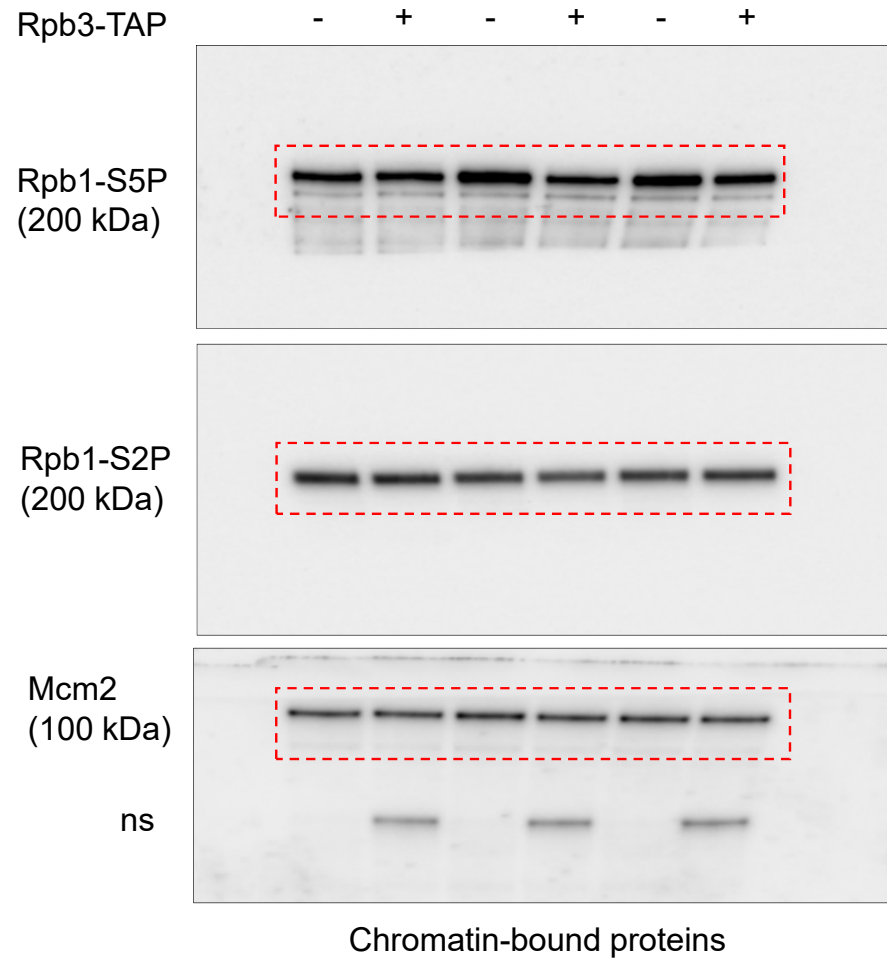

Supplement: Supplementary file 15 — Source Data for Figure 6 [file EMBJ-40-e108439-s014.zip › EMBOJ-2021-108439R_Hurst_et_al_Source_Data_Figure6A.pdf]

# Drop assay Source Data for Figure\_6F

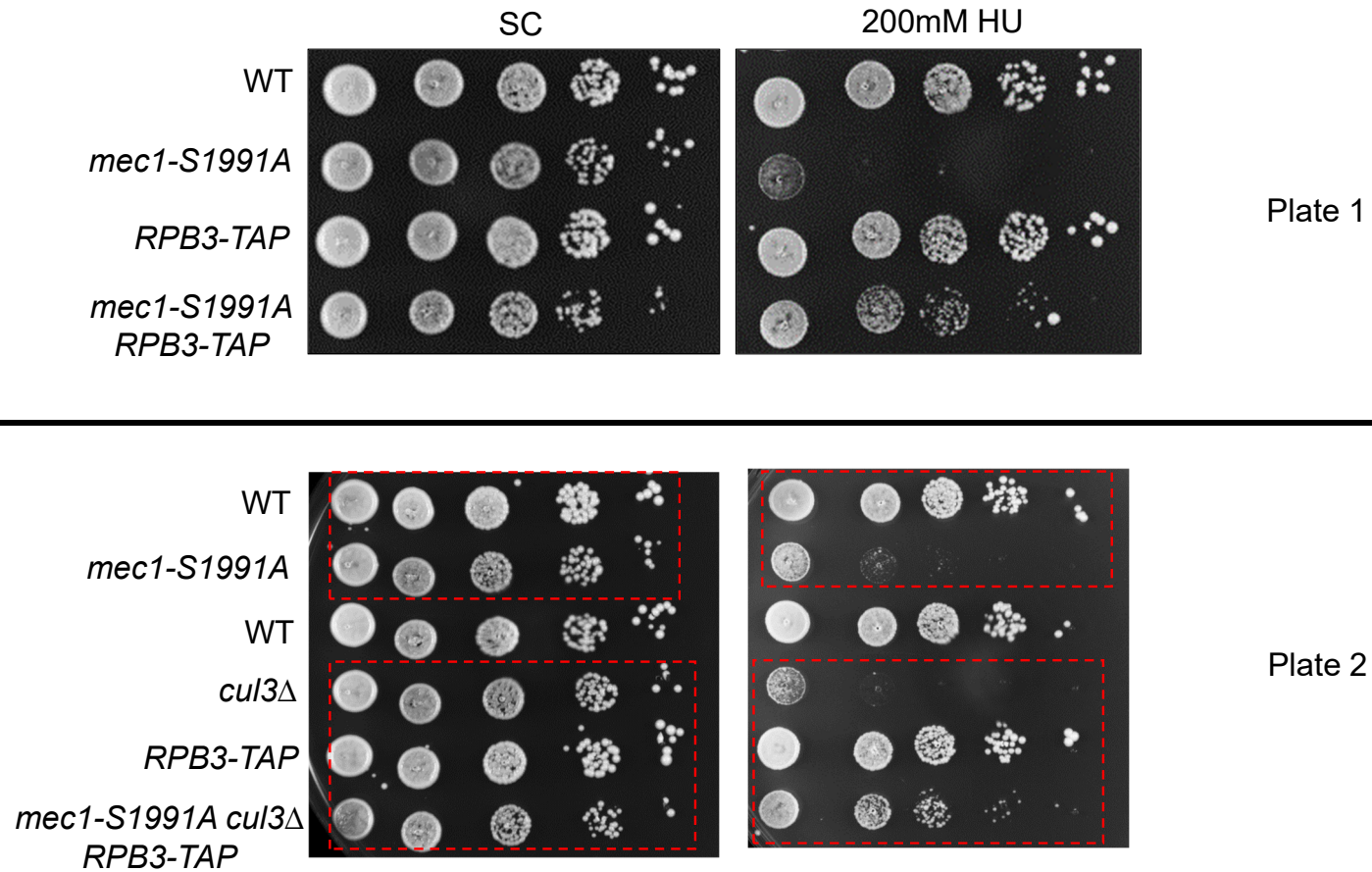

Supplement: Supplementary file 15 — Source Data for Figure 6 [file EMBJ-40-e108439-s014.zip › EMBOJ-2021-108439R_Hurst_et_al_Source_Data_Figure6F.pdf]

Western Blot Source Data for Figure\_8C

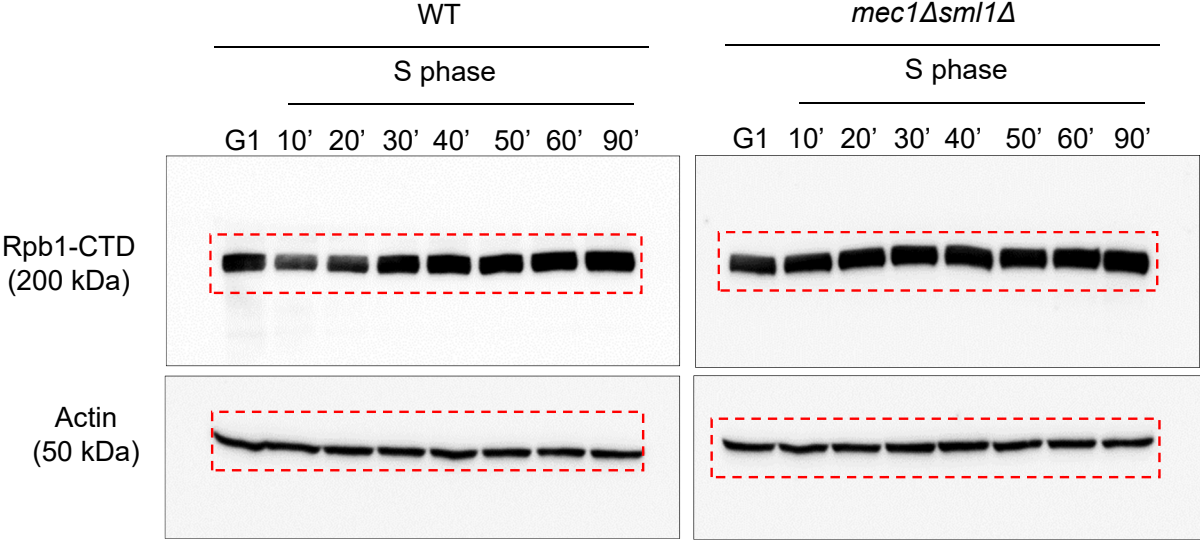

Supplement: Supplementary file 16 — Source Data for Figure 8 [file EMBJ-40-e108439-s001.pdf]
