# Supplementary material for: A regulatory phosphorylation site on Mec1 controls chromatin occupancy of RNA polymerases during replication stress
Source: EMBO J. 2021 Sep 27;40(21):e108439. doi: 10.15252/embj.2021108439 (PMC8561635; doi:10.15252/embj.2021108439)

DNA combing Source Data for Figure_3C

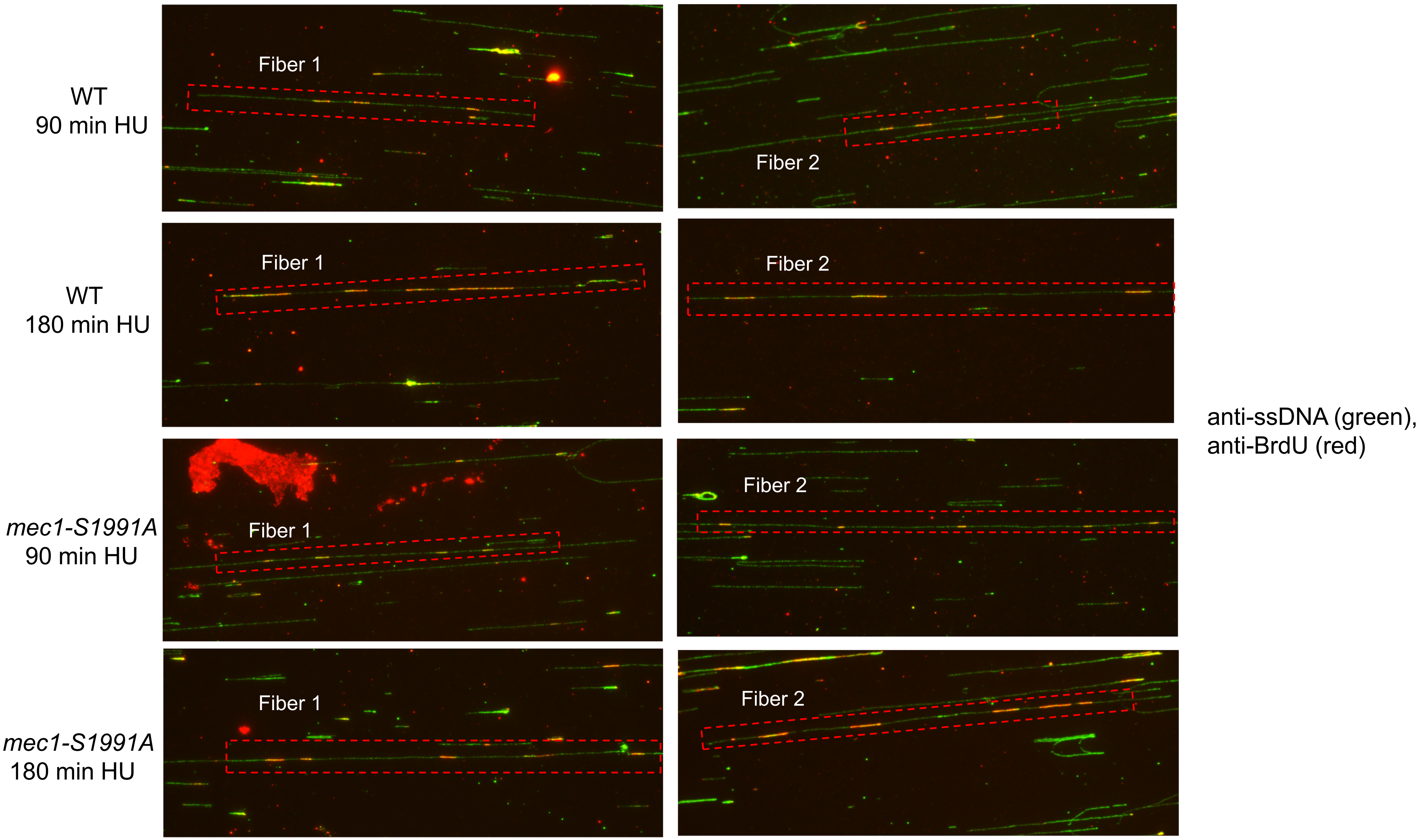

Supplement: Supplementary file 13 — Source Data for Figure 3 [file EMBJ-40-e108439-s008.pdf]
