# Supplementary material for: A regulatory phosphorylation site on Mec1 controls chromatin occupancy of RNA polymerases during replication stress
Source: EMBO J. 2021 Sep 27;40(21):e108439. doi: 10.15252/embj.2021108439 (PMC8561635; doi:10.15252/embj.2021108439)
Supplement: Supplementary file 17 [file EMBJ-40-e108439-s003.pdf]

THE EMBO JOURNAL

A regulatory phosphorylation site on Mec1 controls chromatin occupancy of RNA polymerases during replication stress

Verena Hurst, Kiran Challa, Felix Jonas, Romain Forey, Ragna Sack, Jan Seebacher, Christoph Schmid, Naama Barkai, Kenji Shimada, Susan Gasser, and Jérôme Poli
DOI: 10.15252/embj.2021108439

Corresponding author(s): Jérôme Poli (jerome.poli@igh.cnrs.fr) , Susan Gasser (susan.gasser@fmi.ch), Kenji Shimada (kenji.shimada@fmi.ch)

Review Timeline:

Submission Date:	8th Apr 21
Editorial Decision:	10th May 21
Revision Received:	2nd Aug 21
Editorial Decision:	31st Aug 21
Revision Received:	8th Sep 21
Accepted:	10th Sep 21

Editor: Hartmut Vodermaier

Transaction Report:

(Note: With the exception of the correction of typographical or spelling errors that could be a source of ambiguity, letters and reports are not edited. Depending on transfer agreements, referee reports obtained elsewhere may or may not be included in this compilation. Referee reports are anonymous unless the Referee chooses to sign their reports.)

1st Editorial Decision 10th May 2021

Thank you again for submitting your manuscript on Mec1 phosphorylation controlling RNA polymerases during replication stress for our editorial consideration. It has now been reviewed by three expert referees, whose comments are copied below. As you will see, the referees acknowledge the potential interest of your findings, but also raise a number of concerns that would in our view need to be addressed prior to EMBO Journal publication. I will not repeat them in detail here, since they are well laid-out in the reports, but would like to point out that in addition to answering the various specific issues, it would be important to also deepen the insight into the molecular role of S1991 phosphorylation to some degree, as requested by referee 3.

Pending satisfactory revisions of these points, we would be interested in pursuing a revised manuscript further for EMBO Journal publication. Please note that it is our policy to allow only a single round of major revision, making it important to carefully answer to all referee points at the time of resubmission. Should you require extra time for this in light of the present pandemic situation, or have any particular questions regarding the referees' comments and how to best address them, please do not hesitate to contact me for further discussion already during the early stages of your revision. Our scooping protection (meaning that competing work appearing elsewhere in the meantime will not affect our considerations of your study) remains of course valid also during an extended revision period.

REFEREE REPORTS

Referee #1:

DNA replication and RNA transcription collide on DNA and destabilize chromosome structure. The DNA replication checkpoint resolves this problem by reducing transcription. The authors of this manuscript have shown that RNAPII is removed from chromatin upon checkpoint activated and yeast Mec1 kinase participates in this process. They further extend it in this manuscript and

revealed that a hydroxyurea-induced (S-phase specific) phosphorylation site at Mec1-S1991 works for removal of RNAPII and RNAPIII by HU treatment. The non-phosphorylatable mec1-S1991A mutant, but not phosphomimetic S1991D, is defective in the removal of RNAPs and reduces replication fork progression, leading to HU-sensitive. Moreover, this defect is partially restored by a Rpb3-TAP fusion destabilizing chromatin-bound RNAPII. Although molecular mechanism has not been depicted, they also found that several components controlling RNAPII and RNAPIII transcription are phosphorylated in a Mec1-dependent manner. The authors clearly showed that Mec1 S1991 phosphorylation promotes the removal of RNAP from chromatin, which is important for the DNA replication checkpoint to limit conflict of replication and transcription. This is interesting finding in the field of theDNA replication checkpoint research and I do not have strong reservation for publication. I rather recommend some modifications for improvement, especially #1 and #7 described below.

(Specific points)
1. The authors previously described that mec1-S199A confers sensitivity only to Zeocin. In this manuscript, they found HU-sensitivity using synthetic medium or higher concentration. They thus employed HU-sensitivity. However, we do not know exact reason why mec1-S1991A mutant showed Zeocin sensitivity. To distinguish HU and Zeocin, the authors have better show and discuss the result of Zeocin-sensitivity when Rpb3-TAP is introduced.
2. Fig. 1C, Is cell cycle progression of wild-type and mec1del same? If not, should compare multiple time points.
3. p.7, 2nd paragraph, "To discriminate between a cis effect mediated by direct collision with the replication machinery", I cannot find author's conclusion to this question in the text. The audience will appreciate if the authors discuss it clearly in the text.
4. P10, 2nd line, Figure 2D should read Figure 2E.
5. Figure S4E, Is Maf1 deletion resistant to HU? In Nguyen et al., 2010, maf1del is sensitive to HU in glycerol-medium.
6. p.13, 2nd paragraph, 4th line from the bottom, "the sensitivity of the mec1-S1991A allele for growth on HU was epistatic to cul3Δ and elc1Δ," To me, the sensitivity of the mec1-S1991A allele was epistatic to elc1Δ but not evident to cul3Δ in Figure S4H. The sensitivity of the mec1-S1991A allele for growth on HU was similar to cul3Δ. This result is still consistent with the suggestion that Mec1-S1991 phosphorylation acts through the Elc1-Cul3 ubiquitin ligase complex to promote Rpb1 degradation in the presence of HU. Modification of this explanation will be appreciated.
7. p.13, 2nd paragraph, 7th line, the authors describe that Rpb1 is degraded in mec1-S1991D slightly later (Figure 4F and Figure S4G). Although there is no description, mec1-S1991D suppresses or is epistatic to cul3Δ and elc1Δ. Thus, S1991D seems not to be a simple phosphomimetic mutation but rather bypass the requirement of the Elc1-Cul3 ubiquitin ligase. Does Rpb1 degradation still occur in mec1-S1991D cul3del or elc1del? How do you explain the phenotype of S1991D?

Referee #2:

Conflicts between the replisome and the transcription machinery are a major source of genome

instability. Consequently, many mechanisms have been identified that attempt to alleviate these problems. The Mec1/ATR checkpoint kinase is a key player in the checkpoint response to several types of fork-associated events, including collisions with the transcription machinery. Previous work from the Poli and Gasser groups found that Mec1 functions in concert with the INO80C remodeler to promote the eviction and degradation of RNAPII during replication stress (HU, hydroxyurea). In this manuscript, the authors perform a comprehensive analysis of the role of Mec1 and Mec1 phosphorylation for replication-transcription conflicts during both a normal, unperturbed S phase and during stress induced by HU. Starting with a global proteomic approach, they find that many proteins are lost from chromatin during replication stress, and furthermore, the authors find that RNAPII is lost from chromatin in a Mec1-dependent mechanism even during a normal S phase. They characterize in detail a phosphorylation site within Mec1 (S1991), and they find that a mec1-S1991A allele is sensitive to replication stress conditions and shows genetic interactions with many factors known to influence replication-transcription conflicts. DNA combing studies indicate that Mec1-S1991 phosphorylation is key for normal fork progression and ChIP studies show that it is required for removal of the RNAPII and RNAPIII transcription machinery during S phase. A global phosphoproteome analysis also shows that Mec1-S1991 phosphorylation is also key for modification of many components of the transcription machinery, consistent with a role in resolving replication-transcription conflicts.

In general, this is an outstanding, comprehensive study that illustrates key roles for Mec1 in resolving transcription-replication conflicts. The combination of proteomics and genomic approaches is impressive, and the conclusions are balanced and described in a clear and logical manner. This is an impressive piece of work.

There are only a few issues that should be addressed to further strengthen this work:

(1). I was struck by the ~50% decrease in RNAPII levels at the few target genes analyzed during a normal S phase. The authors should discuss their data as it pertains to the phenomenon of transcriptional buffering - the 2-fold repression of newly replicated genes in response to gene dosage. Are the data distinct or the same? What does RNAPII levels looks like at early replicating genes that are not subject to head-on collisions? Do they observe decreased RNAPII levels? The authors observe a global loss of RNAPII by western blot in a normal S phase - does this impact interpretation of gene dosage buffering studies? These points deserve significant discussion.

(2) Is the loss of RNAPII during an unperturbed S phase observed by western blot (Figure 1E) alleviated in mec1 sml1? This would seem to be a key result, given the previous work on Mec1 and INO80C.

(3) The authors should check the referencing. At least one is missing from the list (Anand et al., 2017).

(4) Figure S2. "co-directional" is mis-spelled in panel headers

(5) Figure S2. It appears that the mec1 sml1 strain has a lower level of RNAPII at genes in G1. The reviewer agrees that the ratio of G1/S is distinct from WT, but the authors should comment.

(6) Table S7. A few yeast strains are lacking GA numbers

Referee #3:

"A regulatory phosphosite on Mec1 controls RNAPII and RNAPIII occupancy during replication stress" by Hurst et al.

This manuscript follows up on an earlier study from the Gasser lab, which showed the existence of a degradative mechanism that targets RNA Pol II after replication stress induced by Hydroxyurea and thereby avoids replication-transcription conflicts. Already the previous manuscript showed that the replication checkpoint kinase Mec1 has a key function in activation of this degradation. The current manuscript now looks at a Mec1 autophosphorylation site (S1991) and its role in activating the removal of RNA Pol II as well as RNA Pol III and extends the findings to an action during an unperturbed S phase.

Overall, this manuscript clearly builds on its predecessor. It advances the previous model and the data is general of high quality and the observed effects are clear, even though at times small. My main concerns are (i) the lack of mechanistic insight into how (auto-)phosphorylation of Mec1 mediates this role in polymerase degradation and (ii) the in some part fragmented presentation of the data, which to some extend obscures the big picture. Therefore, I think the following points should be addressed prior to publication.

Major Points:

1 - a major focus of this manuscript is the S1991 phosphorylation, but the mechanism remains elusive. While I sympathize with the authors' argument that this is because of difficult biochemistry, I would wish for some effort. For example: interactors of WT Mec1-Ddc2 and phosphor-mutant could be determined.

2 - Fig. 1 A and B - I am lacking an explanation how the "chromatome" was determined. In my eyes the experiment would also require a proteome measurement to determine which of the observed changes are due to changes in protein abundance and which to specific interaction with chromatin.

3 - Fig. 1C - Here and elsewhere the authors use antibodies against the C-terminal tail auf Rpb1. Can they be sure that phosphorylation of this CTD does not change detection by the antibody? What antibodies do they used in the first place.

4 - I like that the authors extend their findings with HU to an unperturbed S phase. However, the authors may want to reconsider the presentation. This data is sandwiched between HU experiments and it is not always easy to compare. It is especially tedious (and perhaps not even suitable?) to compare with some data that is part of the 2016 paper. Perhaps, one could consider putting the "unperturbed S phase" data at the end of the MS and find a better way for the comparison.

5 - Figure 2 - Most of the data comes in the form of drop-test that have not been quantified. From these the authors cannot discriminate whether a genetic interaction is additive or synergistic. This is particularly true, given that certain mutants (e.g. arp8delta) have growth phenotypes without DNA damage.

6 ¬- Figure 2C - are the authors confident about faster Rad53 activation and slower inactivation in the S1991A mutant?

7 ¬- Figure 3 - Overall, this figure is quite convincing, but the authors do not comment on the apparent cell-to-cell variation in the recovery from the HU arrest in the population of cells.

8 - Figure 5 - A major part of this figure (and the paper) hinges on the RPB3-TAB "mutant". A clearer demonstration of its destabilizing effect (or a clear reference as to where this has been shown) is lacking

We thank the Referees for their constructive comments and overall support for the publication of our findings. Detailed responses to the referee's suggestions are provided below.

Referee #1:

DNA replication and RNA transcription collide on DNA and destabilize chromosome structure. The DNA replication checkpoint resolves this problem by reducing transcription. The authors of this manuscript have shown that RNAPII is removed from chromatin upon checkpoint activated and yeast Mec1 kinase participates in this process. They further extend it in this manuscript and revealed that a hydroxyurea-induced (S-phase specific) phosphorylation site at Mec1-S1991 works for removal of RNAPII and RNAPIII by HU treatment. The non-phosphorylatable mec1-S1991A mutant, but not phosphomimetic S1991D, is defective in the removal of RNAPs and reduces replication fork progression, leading to HU-sensitive. Moreover, this defect is partially restored by a Rpb3-TAP fusion destabilizing chromatin-bound RNAPII. Although molecular mechanism has not been depicted, they also found that several components controlling RNAPII and RNAPIII transcription are phosphorylated in a Mec1-dependent manner. The authors clearly showed that Mec1 S1991 phosphorylation promotes the removal of RNAP from chromatin, which is important for the DNA replication checkpoint to limit conflict of replication and transcription. This is interesting finding in the field of theDNA replication checkpoint research and I do not have strong reservation for publication. I rather recommend some modifications for improvement, especially #1 and #7 described below.

(Specific points)

1. The authors previously described that mec1-S199A confers sensitivity only to Zeocin. In this manuscript, they found HU-sensitivity using synthetic medium or higher concentration. They thus employed HU-sensitivity. However, we do not know exact reason why mec1-S1991A mutant showed Zeocin sensitivity. To distinguish HU and Zeocin, the authors have better show and discuss the result of Zeocin-sensitivity when Rpb3-TAP is introduced.

This is indeed an interesting point, although it is really a side issue, given the main point of the paper (RNAP removal in S phase and during replication stress). As we documented, the mutation of the Mec1-S1991 phosphorylation site changes the phosphorylation of a large number or proteins in the nucleus, thus it is unlikely that it affects Zeocin resistance through the same pathway of RNAPII removal. This is especially true given that Zeocin sensitivity is not an S phase phenomenon; rather, Zeocin induced lesions are a 9:1 mixture of single to double strand breaks that are repaired mostly by BER or by HR pathways (see Povirk, 1996; and Shimada et al., BioRXIV 2020). Nonetheless, we performed drop assays of *mec1-S1991A +/- RPB3-TAP* on SC + 50 and 100ug/ml of Zeocin and found that the presence of RPB3-TAP did not improve *mec1-S1991A* growth in the presence of Zeocin. These data are now included in **Figure EV3B**. Rpb3-TAP also did not suppress *mec1-S1991A* sensitivity to MMS (**Figure EV3C**).

2. Fig. 1C, Is cell cycle progression of wild-type and mec1del same? If not, should compare multiple time points.

This is an important point given that progression through S phase does alter the level of RNAPII even in the absence of damage. We have performed a G1/α-factor synchronization and release into normal S phase at 25°C and 16°C by FACS to show that cell cycle without stress is not affected in *mec1Δsml1Δ* vs WT. These data are now included in **Figures EV5F and G**.

3. p.7, 2nd paragraph, "To discriminate between a cis effect mediated by direct collision with the replication machinery", I cannot find author's conclusion to this question in the text. The audience will appreciate if the authors discuss it clearly in the text.

We apologize for this oversight. This point is now thoroughly handled in the Results and Discussion sections. For instance we state: "We conclude that RNAPII levels drop in S phase not only at origin-proximal loci upon exposure to HU, but also during an unchallenged S phase, albeit to a lower extent. Lastly, the moderate reduction of RNAPII occupancy at replication sites is distinct from the total transcriptional shutdown of cell-cycle regulated genes and occurs at genes that are located in close proximity to replication forks."

4. P10, 2nd line, Figure 2D should read Figure 2E.

Thanks for pointing this mistake. Figure references is now corrected.

5. Figure S4E, Is Maf1 deletion resistant to HU? In Nguyen et al., 2010, maf1del is sensitive to HU in glycerol-medium.
Thanks for pointing this out. We could confirm that *maf1Δ* cells are sensitive to HU when cells are grown in a medium containing glycerol. At a similar dose of HU when cells are grown in a medium containing glucose, we do not observe differences in sensitivity between *maf1*Δ and WT cells. These drop assay are now included in **Figure EV1D**, and the media-dependence for sensitivity is mentioned.

6. p.13, 2nd paragraph, 4th line from the bottom, "the sensitivity of the mec1-S1991A allele for growth on HU was epistatic to cul3Δ and elc1Δ," To me, the sensitivity of the mec1-S1991A allele was epistatic to elc1Δ but not evident to cul3Δ in Figure S4H. The sensitivity of the mec1-S1991A allele for growth on HU was similar to cul3Δ. This result is still consistent with the suggestion that Mec1-S1991 phosphorylation acts through the Elc1-Cul3 ubiquitin ligase complex to promote Rpb1 degradation in the presence of HU. Modification of this explanation will be appreciated.

We understand that epistasis is difficult to determine when both mutants are defective. In most of the double mutants with *mec1-S1991A* we see additive or synergistic effects, which are now quantified and presented graphically. In combination with *cul3Δ* or *elc1Δ*, *mec1-S1991A* sensitivity to HU is not aggravated. We now include quantitation of multiple drop assays showing that this is the case. Our text now states: "Moreover, the sensitivity of the *mec1-S1991A* allele for growth on HU was similar to that of *cul3Δ*, and combining the *mec1-S1991A* allele with either *cul3Δ* or *elc1Δ* mutation was epistatic, that is the double mutant showed no aggravated lethality on HU (**Figure 5E,F,** and **Figure EV2A,B**). We note that the *mec1-S1991D* allele slightly enhanced *cul3Δ* growth on HU, although not that of *elc1Δ* (**Figure EV2A,B**). Taken together, these results suggest that Mec1-S1991 phosphorylation acts through the Elc1-Cul3 ubiquitin ligase complex to promote Rpb1 degradation in the presence of HU."

7. p.13, 2nd paragraph, 7th line, the authors describe that Rpb1 is degraded in mec1-S1991D slightly later (Figure 4F and Figure S4G). Although there is no description, mec1-S1991D suppresses or is epistatic to cul3Δ and elc1Δ. Thus, S1991D seems not to be a simple phosphomimetic mutation but rather bypass the requirement of the Elc1-Cul3 ubiquitin ligase. Does Rpb1 degradation still occur in mec1-S1991D cul3del or elc1del? How do you explain the phenotype of S1991D?

This is a good point, as the aspartic acid substitution does not exactly phenocopy a phosphorylated S1991. We thank the referee for pointing this out. In repeating these experiments we found that the *mec1-S1991D cul3Δ* and *mec1-S1991D elc1Δ* strains presented in previous **Figures S5B and C** also carried the *RPB3-TAP* allele. Removing the *RPB3-TAP* allele alleviated the observed suppression by *mec1-S1991D* of *cul3Δ* and *elc1Δ* defects on HU. We now include double mutants with *mec1-S1991D* with and without *RPB3-TAP* (**Figure EV2A,B**), and find that the destabilization of RNAPII through Rpb3-TAP does suppress the HU sensitivity of *cul3Δ* or *elc1Δ,* but *mec1-S1991D* does not. The fact that the aspartic acid does not replace a phosphorylation event is common. It can mean either that the turnover of phosphorylation-dephosphorylation is important or that the phospho-S is an important interaction site (e.g. for a set of targets). We cannot rule out either hypothesis. We note that Hustedt et al., 2015 showed that the turnover of phosphor-S1991 is mediated by PPH4, which is a key S phase regulator of Mec1. As for the *S1991D* mutant: we consider this a hypomorph and now state this in the text. We already noticed that the *mec1-S1991D* is a bit more resistant to other genotoxic agents on plates (especially Zeocin on rich media, see drop assay below), suggesting that the hypomorphic phenotypes extend beyond RNAPII sensitivity on HU. We have corrected the text to reflect this and include relevant data in **Figure EV2A and B.**

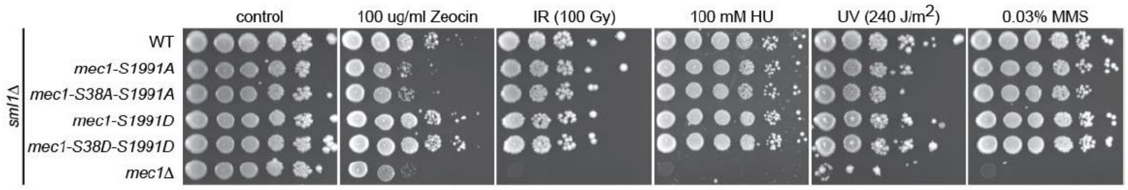

Here we show on YPAD that *mec1-S1991D* is resistant to Zeo but under other damaging conditions it grows like wild-type (see Hustedt et al., Mol Cell, 2015).

Referee #2:

Conflicts between the replisome and the transcription machinery are a major source of genome instability. Consequently, many mechanisms have been identified that attempt to alleviate these problems. The Mec1/ATR checkpoint kinase is a key player in the checkpoint response to several types of fork-associated events, including collisions with the transcription machinery. Previous work from the Poli and Gasser groups found that Mec1 functions in concert with the INO80C remodeler to promote the eviction and degradation of RNAPII during replication stress (HU, hydroxyurea). In this manuscript, the authors perform a comprehensive analysis of the role of Mec1 and Mec1 phosphorylation for replication-transcription conflicts during both a normal, unperturbed S phase and during stress induced by HU. Starting with a global proteomic approach, they find that many proteins are lost from chromatin during replication stress, and furthermore, the authors find that RNAPII is lost from chromatin in a Mec1-dependent mechanism even during a normal S phase. They characterize in detail a phosphorylation site within Mec1 (S1991), and they find that a mec1-S1991A allele is sensitive to replication stress conditions and shows genetic interactions with many factors known to influence replication-transcription conflicts. DNA combing studies indicate that Mec1-S1991 phosphorylation is key for normal fork progression and ChIP studies show that it is required for removal of the RNAPII and RNAPIII transcription machinery during S phase. A global phosphoproteome analysis also shows that Mec1-S1991 phosphorylation is also key for modification of many components of the transcription machinery, consistent with a role in resolving replication-transcription conflicts.

In general, this is an outstanding, comprehensive study that illustrates key roles for Mec1 in resolving transcription-replication conflicts. The combination of proteomics and genomic approaches is impressive, and the conclusions are balanced and described in a clear and logical manner. This is an impressive piece of work.

There are only a few issues that should be addressed to further strengthen this work:

(1). I was struck by the ~50% decrease in RNAPII levels at the few target genes analyzed during a normal S phase. The authors should discuss their data as it pertains to the phenomenon of transcriptional buffering - the 2-fold repression of newly replicated genes in response to gene dosage. Are the data distinct or the same? What does RNAPII levels looks like at early replicating genes that are not subject to head-on collisions? Do they observe decreased RNAPII levels? The authors observe a global loss of RNAPII by western blot in a normal S phase - does this impact interpretation of gene dosage buffering studies? These points deserve significant discussion.

Thank you for pointing out the literature on transcriptional buffering. We do think that the RNAPII removal/degradation we scored in normal S phase is similar to the data described in Bar-Ziv et al., Cell Rep, 2020 where a very transient reduction of RNAPII binding to chromatin was also observed rapidly after cell release into S phase. However, given that the drop in RNAPII in an unchallenged S phase was not dependent on Mec1 activation (Voichek et al., 2018, Molecular Cell 70, 1121–1133), we propose that there could be more than one mechanism at work. In our hands, the drop in RNAPII on HU, as well as the unchallenged S phase, were dependent on Mec1. In the buffering paper Mec1 was only needed for homeostasis (reduced levels after replication) on HU, and not in an unchallenged S phase (Voichek et al., 2018, Molecular Cell 70, 1121–1133). This argues for a “counting” mechanism and/or dilution of a limited RNAPII pool which could function in parallel to what we observe.

However, we note that the data reported earlier were "relative" levels of RNAPII and not absolute levels as we monitor here in our Western blot of total protein and live microscopy. The buffering could arise from multiple mechanisms, for example, there could be "limited loading on to replicated genes" or there could be an active "kicking off RNAPII from each copy partially" to reduce the amount of RNAPII engaged. We find the drop to be detected at the level of the S5 phosphorylated form of RNAPII which argues that levels of initiating polymerase drops. In brief, the homeostasis/buffering mechanism proposed by Barkai's lab and the Mec1-dependent drop that we monitor by ChIP, are compatible, yet may well reflect two parallel pathways.

We have now introduced a discussion of this point in the revised discussion. This concept of homeostasis does not weaken, but rather enriches our observation, and suggests that more than one mechanism may be functioning to turn-down transcription in S phase. We note that Voichek et al., 2018, showed that the delay in histone modifications after replication was limiting RNAPII loading. Since we are measuring the reduction rapidly after entry into S, this is unlikely to be the mechanism at work in this study.

As for gene orientation, we scored a ~ 50% reduction of RNAPII level when cells are in S phase, by ChIP-qPCR, at genes oriented co-directionally (PYK1 and YEF3, **Figure EV5B-C**) and in head-on configuration (snR13; **Fig 8A** and PDC1; **Figure EV5A**) with the replication forks. This is also the case in Bar-Ziv et al., Cell Rep, 2020: authors analyzed RNAPII level at all early replicated genes regardless of their orientation relative to the replication machinery. Neither study argues that the removal is a result of collision, but rather we argue that it occurs to prevent collision.

(2) Is the loss of RNAPII during an unperturbed S phase observed by western blot (Figure 1E) alleviated in mec1 sml1? This would seem to be a key result, given the previous work on Mec1 and INO80C.

Thank you for this question. Mec1 is definitely active even in an unperturbed S phase (Bastos de Oliveira et al., Mol Cell, 2015 and Forey et al., Mol Cell, 2021) but generally it is thought to target components of the replication machinery (e.g. MCM proteins) to ensure replication efficiency. However, the Smolka data also shows that Mec1 is modifying transcriptional machinery upon MMS treatment (Sanford et al., EMBO J., 2021). This is discussed in the revised paper. We have now performed a western blot of Rpb1 in *mec1Δsml1Δ* vs WT to determine whether RNAPII reduction in a normal S phase is also Mec1-dependent (**See Figure 8C-D**). We also include a live cell measurement of Rpb1-GFP intensity in the nucleoplasm when cells are released into unperturbed S phase which show the same Mec1-dependance (**Figure 8E**).

(3) The authors should check the referencing. At least one is missing from the list (Anand et al., 2017). Added

(4) Figure S2. "co-directional" is mis-spelled in panel headers All occurrences are spell-checked.

(5) Figure S2. It appears that the mec1 sml1 strain has a lower level of RNAPII at genes in G1. The reviewer agrees that the ratio of G1/S is distinct from WT, but the authors should comment. This is now mentioned.

(6) Table S7. A few yeast strains are lacking GA numbers. Corrected

Referee #3:

"A regulatory phosphosite on Mec1 controls RNAPII and RNAPIII occupancy during replication stress" by Hurst et al.

This manuscript follows up on an earlier study from the Gasser lab, which showed the existence of a degradative mechanism that targets RNA Pol II after replication stress induced by Hydroxyurea and thereby avoids replication-transcription conflicts. Already the previous manuscript showed that the replication checkpoint kinase Mec1 has a key function in activation of this degradation. The current manuscript now looks at a Mec1 autophosphorylation site (S1991) and its role in activating the removal of RNA Pol II as well as RNA Pol III and extends the findings to an action during an unperturbed S phase.

Overall, this manuscript clearly builds on its predecessor. It advances the previous model and the data is general of high quality and the observed effects are clear, even though at times small. My main concerns are (i) the lack of mechanistic insight into how (auto-)phosphorylation of Mec1 mediates this role in polymerase degradation and (ii) the in some part fragmented presentation of the data, which to some extend obscures the big picture. Therefore, I think the following points should be addressed prior to publication.

Major Points:

1 - a major focus of this manuscript is the S1991 phosphorylation, but the mechanism remains elusive. While I sympathize with the authors' argument that this is because of difficult biochemistry, I would wish for some effort. For example: interactors of WT Mec1-Ddc2 and phosphor-mutant could be determined.

In the manuscript, we determined Mec1 phosphotargets that are dependent on S1991 phosphorylation both during vegetative growth and upon HU-induced replicative stress which include several chromatin remodellers subunits. As *mec1-S1991A* mutant is unable to degrade Rpb1 during HU stress, we hypothesized that it might disrupt the interaction between INO80 and Cdc48-proteasome as this is important for Rpb1 degradation (Lafon et al., Mol Cell, 2015). However, by co-IP experiment, we were able to recover Cdc48 from an INO80-myc immunoprecipitation in either wild-type or *mec1-S1991A* cells, thus more detail on how Mec1-S1991 controls RNAPII degradation (apart from involving Cul3) will be the topic of future studies.

2 - Fig. 1 A and B - I am lacking an explanation how the "chromatome" was determined. In my eyes the experiment would also require a proteome measurement to determine which of the observed changes are due to changes in protein abundance and which to specific interaction with chromatin.
The protocol for the chromatome isolation and analysis is being submitted as a *STAR Protocol* to Cell Press. We attach the protocol for the reviewers' information. However, it is also quite extensively described in Challa et al., Molecular Cell, 2021. Total proteome was performed in parallel, but the values for enrichment or depletion are calculated based on internal normalization within the total proteome or the chromatome, with the two conditions (+ and – replication stress) analysed in parallel with multiple TMT tags (see experimental scheme in Challa et al. attached). On a total proteome level the changes in abundance of nuclear proteins are not detectable because those proteins that change are in low abundance overall when compared to cytoplasmic proteins.

3 - Fig. 1C - Here and elsewhere the authors use antibodies against the C-terminal tail of Rpb1. Can they be sure that phosphorylation of this CTD does not change detection by the antibody? What antibodies do they use in the first place.

This information is now included in detail in the ms. We used commercially available antibodies against the entire pool of Rpb1 clone 8WG16 (from Abcam, reference ab817 and from Biolegends, reference 664906). These antibodies recognize the heptade amino-acids repeats on the Carboxyl-terminal domain (CTD) of Rpb1, and especially unmodified S2 on Rpb1-CTD (Jones et al., JBC, 2004). Several other studies used anti-CTD antibodies (clone 8WG16 or other) to monitor Rpb1 degradation under various conditions including genotoxic agents such as UV (Somesh et al., Cell, 2005 and Heckmann et al., Scientific report, 2019 ; MMS (Lafon et al., Mol Cell, 2015), hydroxyurea (Lafon et al., Mol Cell, 2015, Poli et al., Genes Dev 2016) or to monitor RNAPII level by quantitative ChIP-seq during replication stress (Voichek et al. Science, 2016) or during regular S phase (Bar-Ziv et al., Cell rep, 2020). In Poli et al., Genes Dev 2016, we showed that Rpb1-S5P (Covance anti-Rpb1-CTD-H14 clone, which preferentially recognize Rpb1-S5P) but not Rpb1-Ser2-P (Abcam, Ab5095) is evicted from chromatin during HU-induced stress using chromatin fractionation and we confirmed total Rpb1 degradation by single-cell microscopy by adding a GFP tag at the C-terminal domain of Rpb1. We now added in **Figure 6B** a Western blot probed with an anti-Rpb1-S5P (Clone H14, Covance) and an anti-Rpb1-S2P (abcam, Ab5095) where we observe Rpb1-S5P, but not Rpb1-S2P, degradation in wild-type cells under HU treatment. We also include a living cells measure of Rpb1-GFP intensity in the nucleoplasm when cells are released into unperturbed S phase (**See Figure 8E**).

4 - I like that the authors extend their findings with HU to an unperturbed S phase. However, the authors may want to reconsider the presentation. This data is sandwiched between HU experiments and it is not always easy to compare. It is especially tedious (and perhaps not even suitable?) to compare with some data that is part of the 2016 paper. Perhaps, one could consider putting the "unperturbed S phase" data at the end of the MS and find a better way for the comparison.

We agree and thanks the referee for his suggestion. As proposed, we now first present the data covering the HU response (From Figure 1 to Figure 7) followed by the data obtained in unperturbed S phase at the end of the manuscript (**Figure 8 and EV5**).

5 - Figure 2 - Most of the data comes in the form of drop-test that have not been quantified. From these the authors cannot discriminate whether a genetic interaction is additive or synergistic. This is particularly true, given that certain mutants (e.g. arp8delta) have growth phenotypes without DNA damage.

To the extent that it is possible or helpful, we have quantified the drop assays for double mutants, to be able to justify either additive effects or epistasis. Since colony size is hard to quantify, yet reflects growth efficiency, drop assay quantitation is only partially conclusive. Nonetheless we have done our best (see Methods).

6 ¬- Figure 2C - are the authors confident about faster Rad53 activation and slower inactivation in the S1991A mutant?
We agree that the differences are minor but the tendency is reproducible and the *S1991A* and *1991D* mutants go in opposite directions. We now state that we do not know what the significance of this is.

7 ¬- Figure 3 - Overall, this figure is quite convincing, but the authors do not comment on the apparent cell-to-cell variation in the recovery from the HU arrest in the population of cells.

It is not clear if the reviewer refers to cell to cell variability in combing or FACS. Although cells are synchronized and release simultaneously into S phase, the DNA combing length measurements show a broad distribution because the data are a collection of measures performed on individual DNA molecules emanating from a different cell. In addition there is always locus heterogeneity (presence of natural replication blocks) and stochastic events at any given replication fork. Based on previous literature this degree of variability in fork progression rate is expected, nonetheless we see significant differences between the conditions assayed.

8 - Figure 5 - A major part of this figure (and the paper) hinges on the RPB3-TAB "mutant". A clearer demonstration of its destabilizing effect (or a clear reference as to where this has been shown) is lacking.

The Rpb3 subunit that bear the TAP tag has been shown by crystallography to bind at the interface where RNAPII contacts the PAF1 complex, which controls RNAPII engagement on DNA (Xu et al., Nat Comm, 2017). In the manuscript, we include in **Figure 6C** a ChIP-qPCR showing the impact of Rpb3-TAP on Rpb1 occupancy on chromatin in G1 cells and scored a robust reduced Rpb1 occupancy for several constituvely expressed gene when Rpb3 is Tap-tagged in a WT background. A similar effect was described for the TREX complex that links transcription and mRNA export which shows decreased on occupancy on chromatin when the Tho2 subunit is C-terminally TAP-tagged (Meinel et al., PloS Genet, 2013). We now include a chromatin fractionation in G1 cells showing similar levels of chromatin-bound elongating polymerase (Rpb1-S2P) but a decreased level of initiating polymerase (Rpb1-S5P) in the Rpb3-TAP (**Figure 6A).** Finally, we also correlate decreased RNAPII levels with decreased mRNA steady-state level by RT-qPCR (**See Figure 6D**).

1st Revision - Editorial Decision 31st Aug 2021

Thank you again for your patience during the re-review of your revised manuscript over the summer months. We have now heard back from all three original reviewers, and I am pleased to say that in light of their positive overall assessment, we shall be happy to publish the study in The EMBO Journal, following incorporation of a few remaining requests from the referees (see comments below), as well as the following editorial points:

REFEREE REPORTS

Referee #1:

The authors added several results and modified sentences, which improve the manuscript very well. However, the genetic term "epistatic" in the text is still confusing to me.

p.14, line 10 - 13, "Moreover, the sensitivity of the mec1-S1991A allele for growth on HU was similar to that of cul3Δ, and combining the mec1-S1991A allele with either cul3Δ or elc1Δ mutation was epistatic, that is the double mutant showed no aggravated lethality on HU."
Definition of "epistatic" in Genetics is "The allele that is doing the masking is epistatic to the gene that is being masked (From Genetics 5th ed. [Hartwell, Goldberg, Fischer, Hood & Aquadro eds])".
Thus, I recommend simple expression, "Moreover, the sensitivity of the mec1-S1991A allele for growth on HU was similar to that of cul3Δ, and epistatic to elc1Δ mutation."
The authors' result well supports their idea.

Referee #2:

The authors have fully addressed all of my previous concerns.

Referee #3:

"A regulatory phosphosite on Mec1 controls RNAPII and RNAPIII occupancy during replication stress" by Hurst et al.

The revised version of this manuscript by Poli and coworkers addressed all points of concern of the initial version and is overall greatly improved. As such I support the publication of this version and would just suggest that the authors consider the following points if they want to further improve their manuscript.

1 - In the response to my previous "major point 1" the authors mention co-IP data, but chose to not include this in the current manuscript. I think if the authors chose to not include mechanistic data on S1991, but rather keep this as a focus of a different paper, it should be made clear to the readers, who may expect exactly this type of data in the current manuscript.

2 - I think the logical flow of the paper is much better now. The logic of Figure 7, however, could still be improved, I am not sure the "kinome" and "gene release" parts fit together very well in the current arrangement of the manuscript.

3 - Some of the yeast growth assays appear to be assembled as cut outs from different agar plates. If this suspicion is true, please repeat on the same agar plate. If untrue clearly indicate those strains had grown on the same plate.

4 - typo p10 - "tDNA transcription"

- Please modify the text according to the suggestions of referee 1.
Done.

- Regarding referee 3: please make it clear in the manuscript text that mechanistic follow-up shall be the topic of future investigations (point 1),
Done in the discussion as follows on p21: "Evidence for physical interactions with remodelers or the degradation machinery requires additional study."

and importantly, address point 4 by either showing growth of directly compared yeast colonies on the same plate (e.g. by providing additional source data, as already shown for Fig EV4; or by repeating key assays to ensure conditions are identical); related to that, I note that Fig EV4G seems to lack a visible separation indicator between rows.
Additional source data is now provided for cropped drop assays from which colonies where compared on the same plate (Figure 2D, Figure 2F, Figure 6F, Figure EV1D, Figure EV3B). Drop assays in figures EV2, EV3 (panel B) and EV4 (panel D), which were not done on the same plate, are now clearly separated in different panels (EV2A-D, EV4D,E ; EV3B,C). For main figures (2D, 2F and 6F), a clear separation is added between plates in source data.

On the other hand, further changes in line of Ref 3 pt 2 would not seem needed in my view.

Accepted **10th Sep 2021**

Thank you for submitting your final revised manuscript for our consideration. I am pleased to inform you that we have now accepted it for publication in The EMBO Journal.

EMBO PRESS

YOU MUST COMPLETE ALL CELLS WITH A PINK BACKGROUND ➔

PLEASE NOTE THAT THIS CHECKLIST WILL BE PUBLISHED ALONGSIDE YOUR PAPER

Corresponding Author Name: Kenji Shimada, Susan Gasser, Jérôme Poli.
Journal Submitted to: EMBO J.
Manuscript Number: EMBOJ-2021-108439

Reporting Checklist For Life Sciences Articles (Rev. June 2017)

This checklist is used to ensure good reporting standards and to improve the reproducibility of published results. These guidelines are consistent with the Principles and Guidelines for Reporting Preclinical Research issued by the NIH in 2014. Please follow the journal's authorship guidelines in preparing your manuscript.

A- Figures
1. Data
The data shown in figures should satisfy the following conditions:

- the data were obtained and processed according to the field's best practice and are presented to reflect the results of the experiments in an accurate and unbiased manner.
- figure panels include only data points, measurements or observations that can be compared to each other in a scientifically meaningful way.
- graphs include clearly labeled error bars for independent experiments and sample sizes. Unless justified, error bars should not be shown for technical replicates.
- if n< 5, the individual data points from each experiment should be plotted and any statistical test employed should be justified
- Source Data should be included to report the data underlying graphs. Please follow the guidelines set out in the author ship guidelines on Data Presentation.

2. Captions
Each figure caption should contain the following information, for each panel where they are relevant:

- a specification of the experimental system investigated (eg cell line, species name).
- the assay(s) and method(s) used to carry out the reported observations and measurements
- an explicit mention of the biological and chemical entity(ies) that are being measured.
- an explicit mention of the biological and chemical entity(ies) that are altered/varied/perturbed in a controlled manner.
- the exact sample size (n) for each experimental group/condition, given as a number, not a range;
- a description of the sample collection allowing the reader to understand whether the samples represent technical or biological replicates (including how many animals, litters, cultures, etc.).
- a statement of how many times the experiment shown was independently replicated in the laboratory.
- definitions of statistical methods and measures:
  - common tests, such as t-test (please specify whether paired vs. unpaired), simple χ2 tests, Wilcoxon and Mann-Whitney tests, can be unambiguously identified by name only, but more complex techniques should be described in the methods section;
  - are tests one-sided or two-sided?
  - are there adjustments for multiple comparisons?
  - exact statistical test results, e.g., P values = x but not P values < x;
  - definition of 'center values' as median or average;
  - definition of error bars as s.d. or s.e.m.

Any descriptions too long for the figure legend should be included in the methods section and/or with the source data.

In the pink boxes below, please ensure that the answers to the following questions are reported in the manuscript itself. Every question should be answered. If the question is not relevant to your research, please write NA (non applicable). We encourage you to include a specific subsection in the methods section for statistics, reagents, animal models and human subjects.

USEFUL LINKS FOR COMPLETING THIS FORM

http://www.antibodypedia.com
http://1degreebio.org
http://www.equator-network.org/reporting-guidelines/improving-bioscience-research-repor
http://grants.nih.gov/grants/olaw/olaw.htm
http://www.mrc.ac.uk/Ourresearch/Ethicsresearchguidance/Useofanimals/index.htm
http://ClinicalTrials.gov
http://www.consort-statement.org
http://www.consort-statement.org/checklists/view/32-consort/66-title
http://www.equator-network.org/reporting-guidelines/reporting-recommendations-for-tumo
http://datadryad.org
http://figshare.com
http://www.ncbi.nlm.nih.gov/gap
http://www.ebi.ac.uk/ega
http://biomodels.net/
http://biomodels.net/miriam/
http://jjj.biochem.sun.ac.za
https://osp.od.nih.gov/biosafety-biosecurity-and-emerging-biotechnology/
http://www.selectagents.gov/

B- Statistics and general methods

Please fill out these boxes ➔ (Do not worry if you cannot see all your text once you press return)

1.a. How was the sample size chosen to ensure adequate power to detect a pre-specified effect size?	sample size is chosen based on past experience for a 5-10% significant deviation from random, for measuring fiber length (DNA combing) and subnuclear position (zoning assay). For drop assays serial dilutions were repeated usually as three biological replicates. Proteomics data was performed as biological triplicates, and phosphoproteomic mass spec was performed in duplicate. In general, for biological assays, three independent biological replicates were performed.
1.b. For animal studies, include a statement about sample size estimate even if no statistical methods were used.	NA
2. Describe inclusion/exclusion criteria if samples or animals were excluded from the analysis. Were the criteria pre-established?	NA
3. Were any steps taken to minimize the effects of subjective bias when allocating animals/samples to treatment (e.g. randomization procedure)? If yes, please describe.	Double blind scoring of imaging data is standard in the Gasser lab (two separate operators)
For animal studies, include a statement about randomization even if no randomization was used.	NA
4.a. Were any steps taken to minimize the effects of subjective bias during group allocation or/and when assessing results (e.g. blinding of the investigator)? If yes please describe.	Samples randomization procedure to count fibers lenght (DNA combing) and to determine GAL1-GAL10 position in the nucleoplasm (zoning assay).
4.b. For animal studies, include a statement about blinding even if no blinding was done	NA
5. For every figure, are statistical tests justified as appropriate?	Mann-Whitney rank sum test was used to compare DNA combing measurements. Two-Sided Fischer exact test was used to compare zoning assay frequencies. For enrichment or depletion of phosphopeptides in the proteomics studies Student's paired t-test, with biological replicates n=3 was used; To determine differential abundances in chromatin proteomics, abundance values were log2 transformed after addition of pseudocounts, and then subjected to differential analysis using linear models as implemented in the R package limma (3.44.3). Significance of differential abundance was defined by conjunct minimal threshold criteria for FDR-adjusted p values (empirical Bayes method) and for fold-changes (adj.P.Val < 0.1 and logFC > 0.5).
Do the data meet the assumptions of the tests (e.g., normal distribution)? Describe any methods used to assess it.	For DNA combing data, we observed a normal distribution of track lenght in every conditions measured. Normal distribution is assessed by plotting the frequency of tracks length grouped in bins. For proteomics see above.
Is there an estimate of variation within each group of data?	SEM or SD are presented in each group of data.

Is the variance similar between the groups that are being statistically compared?	Yes.

C- Reagents

6. To show that antibodies were profiled for use in the system under study (assay and species), provide a citation, catalog number and/or clone number, supplementary information or reference to an antibody validation profile. e.g., Antibodypedia (see link list at top right), 1DegreeBio (see link list at top right).	Rpb1-CTD antibody used for western blots and ChIP analysis corresponds to mouse monoclonal 8WG16 (ab817) ; To detect Rpb1-S2P and S5P by western blots, abcam ab5095 and covance clone H14 were used respectively. Rad53 protein was detected with a custom-made mouse monoclonal antibody (GenScript) to the FHA2 domain of Rad53 (Hauer et al., NSMB, 2017). Clone yN-19 from santacruz (sc6680) was used to detect Mcm2; for actin we used Millipore MAB1501 and for tubulin we used Thermofischer MA1-80017. For DNA combing, a purified mouse anti-BrdU clone B44 from BD biosciences (ref 347580) was used and a mouse monoclonal autoanti-ssDNA DSHB (AB_10805144). For Rpb1-CTD ChIP, ab817 was used. For HA-tagged protein ChIP, HA F7 clone from santacruz was used (sc7392).
7. Identify the source of cell lines and report if they were recently authenticated (e.g., by STR profiling) and tested for mycoplasma contamination.	NA

* for all hyperlinks, please see the table at the top right of the document

D- Animal Models

8. Report species, strain, gender, age of animals and genetic modification status where applicable. Please detail housing and husbandry conditions and the source of animals.	Saccharomyces cerevisiae (W303 background)
9. For experiments involving live vertebrates, include a statement of compliance with ethical regulations and identify the committee(s) approving the experiments.	NA
10. We recommend consulting the ARRIVE guidelines (see link list at top right) (PLoS Biol. 8(6), e1000412, 2010) to ensure that other relevant aspects of animal studies are adequately reported. See author guidelines, under 'Reporting Guidelines'. See also: NIH (see link list at top right) and MRC (see link list at top right) recommendations. Please confirm compliance.	NA

E- Human Subjects

11. Identify the committee(s) approving the study protocol.	NA
12. Include a statement confirming that informed consent was obtained from all subjects and that the experiments conformed to the principles set out in the WMA Declaration of Helsinki and the Department of Health and Human Services Belmont Report.	NA
13. For publication of patient photos, include a statement confirming that consent to publish was obtained.	NA
14. Report any restrictions on the availability (and/or on the use) of human data or samples.	NA
15. Report the clinical trial registration number (at ClinicalTrials.gov or equivalent), where applicable.	NA
16. For phase II and III randomized controlled trials, please refer to the CONSORT flow diagram (see link list at top right) and submit the CONSORT checklist (see link list at top right) with your submission. See author guidelines, under 'Reporting Guidelines'. Please confirm you have submitted this list.	NA
17. For tumor marker prognostic studies, we recommend that you follow the REMARK reporting guidelines (see link list at top right). See author guidelines, under 'Reporting Guidelines'. Please confirm you have followed these guidelines.	NA

F- Data Accessibility

18: Provide a "Data Availability" section at the end of the Materials & Methods, listing the accession codes for data generated in this study and deposited in a public database (e.g. RNA-Seq data: Gene Expression Omnibus GSE39462, Proteomics data: PRIDE PXD000208 etc.) Please refer to our author guidelines for 'Data Deposition'.<br><br>Data deposition in a public repository is mandatory for:<br>a. Protein, DNA and RNA sequences<br>b. Macromolecular structures<br>c. Crystallographic data for small molecules<br>d. Functional genomics data<br>e. Proteomics and molecular interactions	Yes, RNA-seq data are deposited on GEO (reference number: GSE180167) ; proteomics data are deposited on PRIDE (reference number PXD027337).
19. Deposition is strongly recommended for any datasets that are central and integral to the study; please consider the journal's data policy. If no structured public repository exists for a given data type, we encourage the provision of datasets in the manuscript as a Supplementary Document (see author guidelines under 'Expanded View' or in unstructured repositories such as Dryad (see link list at top right) or Figshare (see link list at top right).	Done in supplementary document and tables.
20. Access to human clinical and genomic datasets should be provided with as few restrictions as possible while respecting ethical obligations to the patients and relevant medical and legal issues. If practically possible and compatible with the individual consent agreement used in the study, such data should be deposited in one of the major public access-controlled repositories such as dbGAP (see link list at top right) or EGA (see link list at top right).	NA
21. Computational models that are central and integral to a study should be shared without restrictions and provided in a machine-readable form. The relevant accession numbers or links should be provided. When possible, standardized format (SBML, CellML) should be used instead of scripts (e.g. MATLAB). Authors are strongly encouraged to follow the MIRIAM guidelines (see link list at top right) and deposit their model in a public database such as Biomodels (see link list at top right) or JWS Online (see link list at top right). If computer source code is provided with the paper, it should be deposited in a public repository or included in supplementary information.	NA

G- Dual use research of concern

22. Could your study fall under dual use research restrictions? Please check biosecurity documents (see link list at top right) and list of select agents and toxins (APHIS/CDC) (see link list at top right). According to our biosecurity guidelines, provide a statement only if it could.	This research was entirely Biosafety level 1, and has approval of the Swiss National Science foundation, as it was properly registered with the swiss government in the name of Susan Gasser. Toxic chemicals such as Zeocin and proteomics reagents were handled according to biosecurity guidelines.
